# WGS to predict antibiotic MICs for *Neisseria gonorrhoeae*

**DOI:** 10.1093/jac/dkx067

**Published:** 2017-03-10

**Authors:** David W. Eyre, Dilrini De Silva, Kevin Cole, Joanna Peters, Michelle J. Cole, Yonatan H. Grad, Walter Demczuk, Irene Martin, Michael R. Mulvey, Derrick W. Crook, A. Sarah Walker, Tim E. A. Peto, John Paul

**Affiliations:** 1 Nuffield Department of Medicine, University of Oxford, Oxford, UK; 2 National Institute for Health Research Biomedical Research Centre, Oxford, UK; 3 Oxford National Institute for Health Research Health Protection Research Unit, Oxford, UK; 4 Brighton and Sussex University Hospitals NHS Trust, Brighton, UK; 5 National Infection Service, Public Health England, UK; 6 Antimicrobial Resistance and Healthcare Associated Infections (AMRHAI) Reference Unit, National Infection Service, London, UK; 7 Department of Immunology and Infectious Diseases, Harvard TH Chan School of Public Health, Boston, MA, USA; 8 Division of Infectious Diseases, Brigham and Women’s Hospital, Boston, MA, USA; 9 National Microbiology Laboratory, Public Health Agency of Canada, Winnipeg, Manitoba, Canada

## Abstract

**Background:**

Tracking the spread of antimicrobial-resistant *Neisseria gonorrhoeae* is a major priority for national surveillance programmes.

**Objectives:**

We investigate whether WGS and simultaneous analysis of multiple resistance determinants can be used to predict antimicrobial susceptibilities to the level of MICs in *N. gonorrhoeae*.

**Methods:**

WGS was used to identify previously reported potential resistance determinants in 681 *N. gonorrhoeae* isolates, from England, the USA and Canada, with phenotypes for cefixime, penicillin, azithromycin, ciprofloxacin and tetracycline determined as part of national surveillance programmes. Multivariate linear regression models were used to identify genetic predictors of MIC. Model performance was assessed using leave-one-out cross-validation.

**Results:**

Overall 1785/3380 (53%) MIC values were predicted to the nearest doubling dilution and 3147 (93%) within ±1 doubling dilution and 3314 (98%) within ±2 doubling dilutions. MIC prediction performance was similar across the five antimicrobials tested. Prediction models included the majority of previously reported resistance determinants. Applying EUCAST breakpoints to MIC predictions, the overall very major error (VME; phenotypically resistant, WGS-prediction susceptible) rate was 21/1577 (1.3%, 95% CI 0.8%–2.0%) and the major error (ME; phenotypically susceptible, WGS-prediction resistant) rate was 20/1186 (1.7%, 1.0%–2.6%). VME rates met regulatory thresholds for all antimicrobials except cefixime and ME rates for all antimicrobials except tetracycline. Country of testing was a strongly significant predictor of MIC for all five antimicrobials.

**Conclusions:**

We demonstrate a WGS-based MIC prediction approach that allows reliable MIC prediction for five gonorrhoea antimicrobials. Our approach should allow reasonably precise prediction of MICs for a range of bacterial species.

## Introduction

Antimicrobial-resistant *Neisseria gonorrhoeae* is a risk to public health, particularly as emerging resistance to available treatment such as azithromycin and ceftriaxone leaves few treatment options.^1^ Treatment and control strategies depend on reliable monitoring of trends in *N. gonorrhoeae* antimicrobial resistance, which are a major focus of national surveillance programmes, such as GRASP (England and Wales),^2^ Euro-GASP (Europe),^3^ GISP (USA)^4^ and the Canadian national programme.^5^

Several studies have demonstrated the potential of WGS to predict antimicrobial susceptibilities across a range of pathogens, including *N. gonorrhoeae*,^6^^,^^7^*Staphylococcus aureus*,^8^ Enterobacteriaceae^9^ and *Mycobacterium tuberculosis*.^10^*N. gonorrhoeae* antimicrobial resistance mechanisms are well described,^11^ allowing their identification using WGS from national surveillance collections,^7^ cohorts with cefixime^12^ and azithromycin resistance,^6^ and reference collections.^13^ However, the reported ability of WGS to predict antimicrobial susceptibilities in *N. gonorrhoeae* has been variable, e.g. single-resistance determinants accurately predict cefixime, but not azithromycin susceptibilities.^7^

Most WGS studies predict antimicrobial susceptibility categorically as ‘susceptible’ or ‘resistant’. While useful for managing individual patients, this provides less surveillance information than phenotypic MIC measurements, which allow trends over time, and the development and spread of resistance to be monitored with more precision. This is particularly important where prevalent strains have antibiotic MICs near clinical breakpoints.

WGS has been successfully used to predict β-lactam MICs in *Streptococcus pneumoniae*^14^^,^^15^ and azithromycin MICs in *N. gonorrhoeae.*^6^ Therefore, using sequence collections from England, the USA and Canada, we investigated whether WGS and simultaneous analysis of multiple resistance determinants using multivariate regression can be used to predict antimicrobial susceptibilities to the level of MIC for multiple drugs in *N. gonorrhoeae*.

## Methods

### Sample collections and antimicrobial susceptibility testing

Three collections, from Brighton, England,^16^ the USA^12^ and Canada^6^ with antimicrobial susceptibilities determined as part of national surveillance programmes, and WGS from previous studies, were studied. The Brighton samples were unselected consecutive clinical isolates from 3 months of each calendar year, the US samples were enriched for cefixime resistance and the Canadian samples for azithromycin resistance. MICs were determined by agar dilution, for Brighton using the GRASP method,^17^ and in the USA^18^ and Canada using CLSI methods.^6^ Quality control was performed using the 2008 WHO gonorrhoea reference strain panel^19^ and ATCC 49226, with MICs and sequences obtained for these strains in Canada used in the final analysis. The distribution of MICs of cefixime, penicillin, azithromycin, ciprofloxacin and tetracycline was sufficient to determine predictors of resistance. There were insufficient numbers of samples with ceftriaxone resistance, and therefore insufficient numbers with important resistance determinants, to include it in the analysis. EUCAST breakpoints were used to categorize samples as susceptible and resistant: azithromycin, ≤0.25 and >0.5; cefixime, ≤0.125 and >0.125; ciprofloxacin, ≤0.03 and >0.06; penicillin, ≤0.06 and >1; and tetracycline, ≤0.5 and >1 mg/L.^20^

### Genetic determinants of antimicrobial susceptibility

Previously reported genetic determinants conferring reduced antimicrobial susceptibility to the different agents are shown in Table 1. Using WGS, SNPs and resultant amino acid substitutions were determined following mapping to the NCCP11945 reference genome (NC_011035.1) and quality filtering as in De Silva *et al.*,^16^ with the exception of the *penA* and *penB* genes and *mtrR* promoter variants, which were more variable, and were therefore identified from Velvet^21^*de novo* assemblies using BLAST and subsequently aligned using MUSCLE.^22^ BLAST searches of *de novo* assemblies were used to determine the presence/absence of accessory genes. To identify rRNA variants, sequence reads were mapped against a single copy of the NCCP11945 23S RNA gene using BWA mem^23^ with default settings. Base counts were determined using SAMtools,^24^ enabling estimation of the proportion of gene copies with relevant mutations.

**Table 1:** Susceptibility-modifying genetic elements.^11^

Gene/element	Characteristic	Summary	Reference	AZM	CFX	CIP	PEN	TET
*penA*	allele	reduced β-lactam acetylation of PBP2	^11^^,^^32^^,^^33^		✓		✓	
	SNPs: A311V, I312M, V316T, V316P, T483S, A501V, N512Y, G545S, A501P, A501V, A501T, G542S, P551S, P551L	*penA* alleles were defined as described in the Methods section, and represent commonly occurring combinations of SNPs	^11^^,^^25^		✓			
	SNPs: D345a, F504L, A510V, A516G, H541N, P551S, P551L, P552V, K555Q, I556V, I566V, N573a, A574V, A311V, I312M, V316T, V316P, T483S, A501V, N512Y, G545S, A501P, A501V, A501T, G542S, P551S, P551L	additional contributions of individual SNPs were also investigated	^11^^,^^34^				✓	
*mtrR* promoter disruption	deletion of A in repeat (–35A)	overexpression of MtrCDE efflux pump	^35^^,^^36^	✓	✓		✓	✓
	A → C in repeat (–38)		^6^^,^^37^					
	2 bp insertion		^36^					
	mtr120	novel promoter for MtrCDE efflux pump expression	^38^	✓	✓		✓	✓
*mtrR*	A39T	overexpression of MtrCDE efflux pump	^39^	✓	✓		✓	✓
	G45D		^39^					
	truncation		^13^					
*penB* (porB1b)	G120K	reduced influx	^40^		✓		✓	✓
	A121D/N		^40^					
*ponA* (*ponA*1 allele)	L421P	reduced β-lactam acylation of PBP1	^41^		✓		✓	
*pilQ*	E666K	reduced influx via pore-forming secretin PilQ	^42^		✓		✓	
*bla*_TEM_	*bla*_TEM_-1/*bla*_TEM_-135-encoding plasmids	penicillinase	^43^^,^^44^				✓	
23S rRNA	C2611T	four copies of these genes present, increasing resistance with increased number of copies with SNPs via decreased binding to 50S ribosome	^45^	✓				
	A2059G		^46^					
*erm*(B), *erm*(C), *erm*(F)	presence	methylate 23S RNA to block binding	^47^	✓				
*macAB*	promoter mutation	efflux pump overexpression	^48^	✓				
*mef*	presence	efflux pump	^49^	✓				
*ere*(A), *ere*(B)	presence	macrolide esterase	^37^	✓				
*gyrA*	S91F	reduced quinolone binding to DNA gyrase	^13^^,^^50^			✓		
	D95N/G		^13^^,^^50^					
*parC*	D86N	reduced quinolone binding to topoisomerase IV	^13^			✓		
	S87R/I/W		^13^					
	S88P		^13^^,^^50^					
	E91K		^13^^,^^50^					
*norM*	promoter mutation	overexpression of efflux pump	^51^			✓		
*rpsJ*	V57M	reduced affinity of 30S ribosome for tetracycline	^52^					✓
*tetM* plasmid	Dutch/American plasmid	TetM resembles elongation factor G, binds 30S ribotype and prevents tetracycline binding	^53^^,^^54^					✓

AZM, azithromycin; CFX, cefixime; CIP, ciprofloxacin; PEN, penicillin; TET, tetracycline.

Ticked boxes indicate that the genetic determinant affects the indicated antimicrobial. The A → C nucleotide substitution 38 bases upstream of *mtrR* is found in WHO-P like and mosaic *Neisseria meningitidis*-like *mtrR* promoter sequences.^6^

### Statistical methods

Multivariate linear regression models were used to identify genetic predictors of MIC. Measured MIC values were converted to a log_2_ scale, such that a one-unit change is equivalent to a single doubling dilution, e.g. log_2_(MIC) = 3 represents MIC = 8 mg/L and log_2_(MIC) = 4 represents MIC = 16 mg/L. Log_2_(MIC) values were used as the (approximately normally distributed) outcome in multivariate linear regression models, and the Table 1 genetic determinants as potential predictors, such that the model coefficients associated with each genetic determinant can be used for MIC prediction. The models are additive on the log_2_ scale, i.e. the log_2_(MIC) value is predicted by adding the coefficients for each genetic determinant to the model constant term [which is equivalent to the WT log_2_(MIC)]. The predicted MIC is then 2 to the power log_2_(MIC). Predicted MICs are presented rounded to the nearest doubling dilution, rounding log_2_(MIC), prior to conversion back to the absolute scale.

Where phenotypic MIC values were observed to be below or above the quantification limits, e.g. <0.06 or >16 mg/L, the actual MIC was assumed to be the adjacent doubling dilution, e.g. 0.03 and 32 mg/L, respectively, for the purposes of model fitting. For genetic determinants with multiple non-WT alleles, where there were <10 observations for a given allele, the variable was collapsed to a binary WT/non-WT variable, identifying amino acid substitutions by the mutation site only. Otherwise, determinants were included as categorical variables with each non-WT allele specified separately. The only exception made to this was for a single isolate in the dataset with *penA* A501P,^25^ as this, combined with *penA* XXXIV, confers high-level cefixime resistance. A separate coefficient for *penA* A501P is presented in the results, but not used for model predictions, as only a single sample was available.

*penA* alleles were determined using a maximum likelihood phylogenetic tree of previously published *penA* alleles and those available in GenBank. The closest matching published *penA* allele was identified for each sample using BLAST. Where <10 samples matched a given allele, this allele was merged with the nearest neighbour on the tree, and the process repeated until all alleles considered had ≥10 samples (Figure S1, available as Supplementary data at *JAC* Online). Previously reported SNPs in *penA* were also considered as potential additional predictors, as individual SNPs may affect resistance, against a background of otherwise identical/similar *penA* alleles.^25^

Univariate regression coefficients were calculated for all antimicrobial-relevant/specific genetic determinants listed in Table 1. Given the large number of potential genetic determinants, to reduce model over-fitting, the final model was chosen using backwards selection, starting from the model including all genetic determinants, and then removing one determinant at a time, until the lowest possible Akaike information criterion (AIC) was obtained. AIC was used rather than more stringent *P* value thresholds (such as *P *>* *0.05) because *a priori* determinants were considered plausibly resistance associated. Alleles were considered as categorical variables, i.e. all alleles were included if the factor as a whole improved the AIC. The rRNA A2059G mutation was only present at 0 or 4 copies and was therefore treated as a categorical variable. The rRNA C2611T mutation was present at 0, 1, 2, 3 and 4 copies, and was modelled as a continuous variable, after ensuring model fit using the AIC was not improved by treating it as a categorical variable. Excluded determinants were added back one at a time to the final model to check this did not improve the AIC; any that did were retained in the final model. Interactions between genetic determinants were then tested, e.g. to detect a saturation effect, where further determinants in the same pathway do not increase the MIC beyond a threshold, or conversely for synergy between determinants. Pairwise interactions between all model terms were tested, initially one at a time; all interactions with a Wald *P* value <0.01 were added to the model, and backwards selection repeated, requiring multivariate Wald *P* values <0.01 to include an interaction in the final model. This pre-specified approach to interactions is deliberately more conservative than the AIC, which is used to identify main effects, to account for multiple potential interaction pairs tested and minimize over-fitting. Errors and model MIC predictions were estimated using leave-one-out cross-validation, where the final model (including interaction terms) is fitted using all samples except one, which is then used to predict the outcome for the excluded sample, repeated over all samples in the dataset. Analyses were performed using STATA version 14.1 (StataCorp, College Station, TX, USA).

## Results

**Figure 1:**
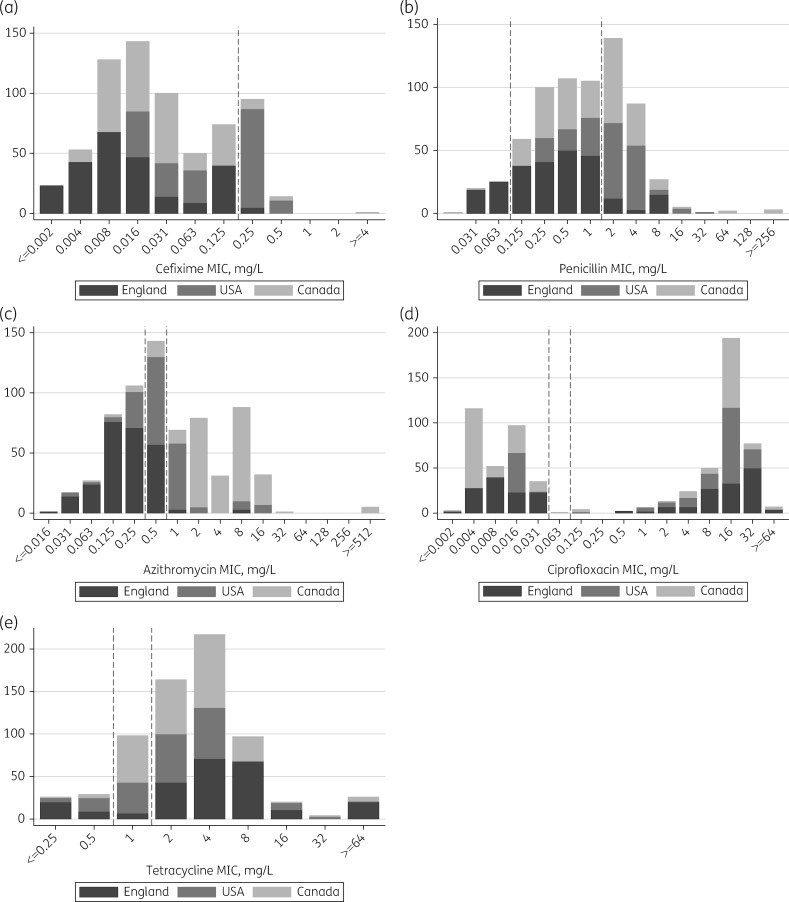
Distribution of measured MICs by country. EUCAST breakpoints for susceptibility and resistance (shown as dashed lines) are: azithromycin, ≤0.25 and >0.5; cefixime, ≤0.125 and >0.125; ciprofloxacin, ≤0.03 and >0.06; penicillin, ≤0.06 and >1; and tetracycline, ≤0.5 and >1 mg/L.^20^

Two hundred and forty-nine isolates from Brighton (July 2004–February 2014), 186 from the USA (January 2009–December 2010), 237 from Canada (January 1989–July 2014) and the 8 WHO 2008 and ATCC 49226 reference strains were included. The distribution of 681 measured MICs for cefixime, penicillin, azithromycin, ciprofloxacin and tetracycline is shown in Figure 1: 110 (16%) samples were resistant to cefixime, 264 (39%) to penicillin, 305 (45%) to azithromycin, 377 (55%) to ciprofloxacin and 528 (78%) to tetracycline based on EUCAST breakpoints. Only ciprofloxacin had a clear bimodal distribution in MICs divided by an MIC breakpoint.

Using WGS, all genetic determinants present in the 2008 WHO reference strain panel^13^^,^^19^ were recovered with 100% concordance. The univariate coefficients for each genetic determinant and the final multivariate model for each antibiotic are given in Table S1. The numbers of samples included in the final multivariate model with completely determined genotypes were 670 for cefixime, 672 for penicillin (2 ambiguous *penA* alleles; relevant *penA* SNPs not determined for 9 and 7 samples, respectively, due to incomplete *de novo* assemblies), 676 for ciprofloxacin (5 samples without *parC* variants determined) and 681 for azithromycin and tetracycline.

For cefixime, *penA* allele, and *mtrR*45, *penA*501/512, mtr120 and *penB* mutations were independent predictors of MIC, with *penA* XXXIV associated with the greatest MIC increase, 8.3-fold (multivariate regression coefficient 3.06, 95% CI 2.19–3.98, *P *<* *0.001; MIC fold increase calculated by 2 to the power of the regression coefficient, i.e. 2^3.06^). The single sample with A501P, showed a predicted MIC increased by a further 26.7-fold. *penA* XV had a negative coefficient (–0.44), indicating this lineage may represent WT MIC, rather than the closely related M32091 (Figure S1) chosen as the baseline allele. *penB*121 mutations also had a negative coefficient, but as this finding was based on three samples it may represent model over-fitting. All potential determinants of penicillin MIC were included in the final model with the exception of *penB*121; the *bla*_TEM_ β-lactamase increased MICs the most, 23.4-fold (coefficient 4.55, 3.80–5.31, *P *<* *0.0001). rRNA mutations were the strongest determinants of azithromycin MIC, along with *erm* genes (although these were only found in three isolates), and *mtrR* mutations and promoter disruption. The rRNA A2059G allele was present in either zero or four copies, four copies increased MICs 375-fold (coefficient 8.55, 7.69–9.40, *P *<* *0.001), and C2611T rRNA mutations present in between zero and four copies, increased MICs 2.9-fold (coefficient 1.52, 1.25–1.79, *P *<* *0.001) per copy. *gyrA* and *parC* mutations predicted ciprofloxacin MIC, with *gyrA*95 mutations having the greatest effect, 16.8-fold MIC increase (coefficient 4.07, 2.86–5.29, *P *<* *0.001). Finally, tetracycline MICs were most impacted by the presence of *tetM*, resulting in a 126-fold MIC increase (coefficient 6.98, 6.36–7.60, *P *<* *0.001), but also by *penB* and *rpsJ* mutations and *mtrR* mutations and promoter disruption.

In addition, the study country [a composite of the testing laboratory and method: CLSI versus GRASP, the isolate origin, and the sampling frame (US samples enriched for cefixime resistance, and Canadian samples for azithromycin resistance)] was independently predictive of MIC for all agents, and not just those enriched for in the sampling frame. Compared with England, cefixime, penicillin and azithromycin MICs were all higher for the USA and Canada (all *P *<* *0.001), ciprofloxacin MICs were lower for the USA (*P *<* *0.001) and tetracycline MICs were lower for the USA and Canada (*P *<* *0.001). For example, cefixime MICs were 2.6-fold (coefficient 1.36, 1.18–1.54, *P *<* *0.001) and 1.5-fold (coefficient 0.60, 0.42–0.78, *P *<* *0.001) higher for the USA and Canada, respectively, compared with England.

Several interaction terms had negative coefficients (Table S1), i.e. the combination of two genetic determinants produced a smaller increase in MIC than the multiple (sum on the log scale) of their effects when found alone. For example, for tetracycline with *rpsJ* 57 and *tetM*, the addition of *rpsJ* 57 to *tetM* does not further increase MIC [6.3-fold increase in MIC with *rpsJ* 57 alone (coefficient 2.66), 126-fold with *tetM* alone (coefficient 6.98) and 124-fold with both (coefficients 2.66 + 6.98 – 2.69)]. Other examples include *tetM* with *mtrR* 45 and *mtrR* promoter deletion, for ciprofloxacin *parC*87 and 91 mutations. Positive interaction coefficients indicate potential synergy, e.g. for tetracycline, the combination of *penB*120 and *mtrR* promoter deletion increases MIC an additional 1.5-fold (coefficient 0.55) compared with the multiple of their effects alone.

**Figure 2:**
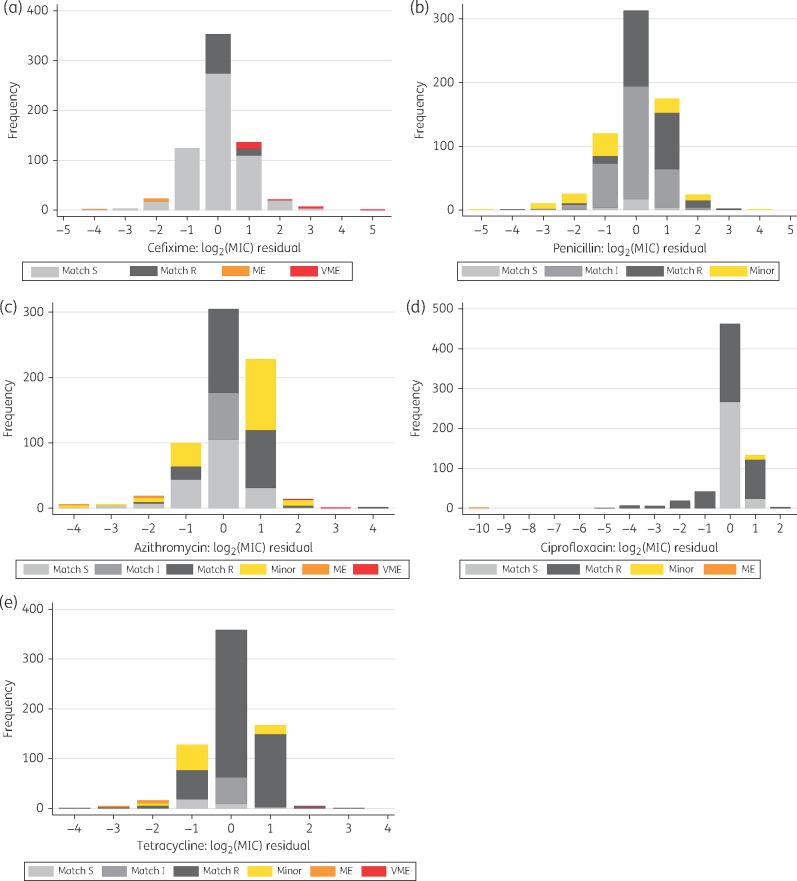
Accuracy of predicted MICs. The difference between the measured and predicted MICs is shown on a log_2_ scale, such that a difference of 1 represents a difference of 1 doubling dilution. A VME is where the predicted susceptibility is susceptible when the measured susceptibility is resistant; an ME is where the predicted susceptibility is resistant, but the measured susceptibility is susceptible; and minor errors denote where the phenotype is susceptible and the prediction intermediate, or the phenotype is intermediate and the prediction is resistant, or vice versa in both cases. S, susceptible; I, intermediate; R, resistant; ME, major error; VME, very major error.

The accuracy of predicted MICs, determined using the cross-validation, is shown in Figure 2 and summarized in Table 2. Overall 1785 (53%) of 3380 log_2_(MIC) values were predicted to the nearest whole number, 3147 (93%) within ±1 doubling dilution and 3314 (98%) within ±2. Exact matches between the predicted and observed MICs were obtained for 52%, 47%, 44%, 58% and 53% of cefixime, penicillin, azithromycin, ciprofloxacin and tetracycline tests, respectively, and matches within ±1 doubling dilution for 91%, 91%, 93%, 94% and 96%, respectively. The corresponding cross-validated root-mean-squared errors (the average difference between MIC predictions and measured phenotypic MICs) and pseudo-*R*^2^ values (the proportion of variation in MIC explained by the model) were 0.89, 0.97, 0.96, 0.96 and 0.73, and 0.81, 0.79, 0.85, 0.96 and 0.80, respectively.

**Table 2:** Summary of model predictions

		Phenotype			% MIC match						Cross-validation	
		
	*n*	S	I	R	0	%	±1	%	±2	%	RMSE	pseudo-*R*^2^
Cefixime	670	560		110	350	52	613	91	657	98	0.89	0.81
Penicillin	672	46	365	261	315	47	610	91	657	98	0.97	0.79
Azithromycin	681	233	143	305	303	44	633	93	666	98	0.96	0.85
Ciprofloxacin	676	293	11	372	459	68	638	94	660	98	0.96	0.96
Tetracycline	681	54	98	529	358	53	653	96	674	99	0.73	0.80
Overall	3380	1186	617	1577	1785	53	3147	93	3314	98		
	

	Genotype concordance													
	match S	% phenotypically S	match I	% phenotypically I	match R	% phenotypically R	minor error	% *n*	ME, {\it n}	ME rate, % phenotypically S	ME rate, 95% CI	VME, {\it n}	VME rate, % phenotypically R	VME rate, 95% CI

Cefixime	553	99			92	84			7	1.3	0.5, 2.6	18	16.4	10.0, 24.6
Penicillin	25	54	320	88	237	91	90	13	0	0.0	0.0, 7.7	0	0.0	0.0, 1.4
Azithromycin	193	83	72	50	245	80	165	24	4	1.7	0.5, 4.3	2	0.7	<0.1, 2.3
Ciprofloxacin	291	99	0	0	372	100	11	2	2	0.7	<0.1, 2.4	0	0.0	0.0, 1.0
Tetracycline	32	59	53	54	515	97	73	11	7	13.0	5.4, 24.9	1	0.2	<0.1, 1.0
Overall	1094	92	445	72	1461	93	339	17	20	1.7	1.0, 2.6	21	1.3	0.8, 2.0
	

	Genotype concordance, allowing phenotype variation ±1 doubling dilution													
	match S	% phenotypically S	match I	% phenotypically I	match R	% phenotypically R	minor error	% *n*	ME, {\it n}	ME rate, % phenotypically S	ME rate, 95% CI	VME, {\it n}	VME rate, % phenotypically R	VME rate, 95% CI

Cefixime	553	99			105	95			7	1.3	0.5, 2.6	5	4.5	1.5, 10.3
Penicillin	35	76	352	96	253	97	32	5	0	0.0	0.0, 7.7	0	0.0	0.0, 1.4
Azithromycin	218	94	135	94	301	99	21	3	4	1.7	0.5, 4.3	2	0.7	<0.1, 2.3
Ciprofloxacin	291	99	11	100	372	100	0	0	2	0.7	<0.1, 2.4	0	0.0	0.0, 1.0
Tetracycline	46	85	94	96	528	100	5	1	7	13.0	5.4, 24.9	1	0.2	<0.1, 1.0
Overall	1143	96	592	96	1559	99	58	3	20	1.7	1.0, 2.6	8	0.5	0.2, 1.0

S, susceptible; I, intermediate; R, resistant; RMSE, root-mean-square error.

The top panel summarizes the observed phenotype for each antimicrobial, and the number and percentage of isolates with a matching MIC prediction within 0, ±1 and ±2 doubling dilutions. Indicators of model fit are given based on leave-one-out cross-validation. The middle panel shows the concordance between the phenotypic susceptibility (susceptible, intermediate, resistant) and the genetic prediction based on the MIC prediction and EUCAST breakpoints.^20^ A VME is where the predicted susceptibility is susceptible when the measured susceptibility is resistant; an ME is where the predicted susceptibility is resistant, but the measured susceptibility is susceptible; and minor errors denote where the phenotype is susceptible and the prediction intermediate, or the phenotype is intermediate and the prediction is resistant, or vice versa in both cases. The bottom panel presents the same analysis as the middle panel, but allowing for the true phenotype to be ± 1 doubling dilution from the observed phenotype. *penA* genotypes could not be determined for two samples; these samples were excluded from the cefixime and penicillin models. A further nine and seven samples were excluded from the cefixime and penicillin models, respectively, as the *penA* SNPs could not be determined due to incomplete *de novo* assemblies. *parC* variants could not be determined for five samples; these samples were excluded from the ciprofloxacin model.

The overall very major error (VME; phenotypically resistant, WGS-prediction susceptible) rate was 21/1577 (1.3%, 95% CI 0.8%–2.0%) and the major error (ME; phenotypically susceptible, WGS-prediction resistant) rate was 20/1186 (1.7%, 1.0%–2.6%). VME rates for cefixime, penicillin, azithromycin, ciprofloxacin and tetracycline were 16.4% (95% CI 10.0%–24.6%), 0% (0.0%–1.4%), 0.7% (<0.1%–2.3%), 0.0% (0.0%–1.0%) and 0.2% (<0.1%–1.0%) respectively. Hence, for all antimicrobials, except cefixime, regulatory thresholds for antimicrobial susceptibility performance were met (target 95% CI ≤1.5%–7.5%).^26^ ME rates were 1.3% (0.5%–2.6%), 0% (0.0%–7.7%), 1.7% (0.5%–4.3%), 0.7% (<0.1%–2.4%) and 13.0% (5.4%–24.9%), respectively, meeting regulatory thresholds (≤3%) with the exception of tetracycline. Of the VMEs for cefixime, 13/18 (72%) fell near the breakpoint (Figure 2a); however, excluding samples where the discrepancy in predicted MIC was only a single doubling dilution, the VME rate was still 4.5% (1.5%–10.3%).

If epistasis, beyond simple pairwise interactions, involving multiple known determinants were to explain the majority of the variation not captured by the models, fixed combinations of known variants would be expected to produce fixed MICs. Therefore, for each antimicrobial, and for each combination of genetic determinants with ≥10 samples, we plotted observed MICs (Figures S2–S6), demonstrating similar variability within these fixed combinations to that seen in the model residual plots (Figure 2).

We also investigated if analysis of single determinants could better explain which isolates were resistant for cefixime. In keeping with previous reports^12^ and other data,^7^*penA* XXXIV was a sensitive predictor of resistance, present in 106/110 resistant strains [sensitivity 96%, VME rate 3.6% (95% CI 1.0%–9.0%)]. However, taken alone it lacks specificity when all included cohorts, including those not selected on the basis of their cefixime susceptibility, are considered, with 83/560 susceptible strains (42 Brighton, 34 Canada, 7 USA) also containing *penA* XXXIV [specificity 85%, ME rate 14.8% (12.0%–18.0%)]. Additionally, other resistance determinants contributed to the final predicted MICs; in phenotypically resistant isolates the median (IQR) [range] number of resistance determinants present as main effects in the final model (excluding country) other than *penA* allele was 4 (4–4) [2–5] compared with 2 (1–3) [0–5] in susceptible isolates. Similarly, with penicillin there was evidence of multiple factors contributing to resistance, with 4 (4–4) [2–6] determinants other than the *penA* allele in resistant isolates and 1 (0–2) [0–4] in susceptible isolates. A single mechanism may be sufficient to explain some azithromycin resistance, with multiple other mechanisms required to explain other resistance: 1 (1–2) [0–3] in resistant isolates and 1 (0–1) [0–2] in susceptible isolates. Of 305 resistant isolates, 165 (54%) had ≥1 rRNA mutation copy (either C2611T or A2059G) and 203 (67%) had *mtrR* promoter disruption compared with 1 (0.4%) and 95 (41%), respectively, in 233 susceptible isolates. In phenotypically ciprofloxacin-resistant isolates, the number of resistance determinants was 3 (3–3) [1–4] compared with 0 (0–0) [0–3] in susceptible isolates. Of the ciprofloxacin-resistant isolates, 319/372 (86%) had non-WT variants at *gyrA*91, 95 and *parC*87. Finally, tetracycline-resistant isolates had 3 (2–3) [1–5] resistance determinants compared with 0.5 (0–2) [0–3] in susceptible isolates.

## Discussion

We demonstrate a WGS-based MIC prediction approach and show that it allows reliable MIC prediction for five gonorrhoea antimicrobials. We predicted MIC within one doubling dilution for 93% of samples, and within two for 98%. This is comparable to routine phenotypic performance: acceptable variation in MIC measurement for type strains in national surveillance programmes is typically ±1 doubling dilution and sometimes greater.^27^

Combining MIC predictions and clinical breakpoints resulted in VME and ME rates within regulatory targets,^26^ with the exception of VME rates for cefixime and ME rates for tetracycline. The majority of cefixime errors arose from single doubling dilution discrepancies in MIC prediction near the breakpoint. Using an alternative approach,^7^^,^^12^ based on the presence/absence of *penA* XXXIV as a determinant of cefixime resistance, VMEs approached acceptable levels [3.6% (95% CI 1.0–9.0%)], but at the expense of increased MEs (14.8%). Almost identical VME/ME rates (3.6%/15.2%) were obtained using a breakpoint one doubling dilution lower than the EUCAST value with the predicted MICs. Some of the very marked phenotype/prediction differences may also be the result of laboratory labelling errors, but we were unable to re-phenotype and re-sequence all such isolates in this study.

We detected MIC variation based on the country of testing, despite phenotypes being determined using gold-standard methods in national reference laboratories. This variation might be partly explained by the sampling frames used, with US isolates enriched for cefixime resistance and Canadian isolates enriched for azithromycin resistance. These isolates might be more likely to contain otherwise unexplained resistance to cefixime and azithromycin, respectively, which is attributed to the country of testing. However, we observed variation across all antimicrobials investigated, and with by-country in MICs following different directions by antimicrobial MICs: higher MICs for cefixime, penicillin and azithromycin in North America, and lower MICs for ciprofloxacin and tetracycline. The differences could also be due to differences between the CLSI testing methods used in North America (coefficients for the USA and Canada tended to be similar), and the GRASP methods used in England and Wales. We chose to model the study country rather than the testing method to be transparent with respect to any possible biases introduced by the country-specific sampling frames. However, there were also differences between the USA and Canada despite the same testing method being used, which may be due to differences in unmeasured strain background in these countries, but may also represent inter-laboratory measurement variation. Determining the exact contribution of each mechanism to differences between countries is not possible with the current study; however, the differences highlight a potential benefit from greater standardization of phenotyping to facilitate evaluation of trends globally.

The genetic determinants with the greatest impact on MIC are broadly in keeping with previously published observations.^6^^,^^11^ It should be noted that the approach taken does not necessarily define the mechanistically most important resistant determinants, but simply those that are statistically most informative to predict MIC. Where multiple mechanisms typically coexist, this collinearity may lead to only one mechanism remaining in the final model, and its associated coefficient represents the overall effect of these multiple mechanisms.

Our data demonstrate that several different mechanisms can together contribute to determine MICs: a strength of the multivariate regression approach used is that these mechanisms can be accounted for simultaneously. We noted examples where the presence of the same mechanism can result in a MIC just above or below the breakpoint, either due to the presence/absence of other mechanisms as observed for azithromycin in this study, or potentially due to testing methods, e.g. for cefixime. While breakpoints are selected across all strains, based on the likelihood of clinical treatment success, it remains to be seen if some mechanisms are more important than others in determining efficacy of treatment. Wider deployment of WGS may facilitate determination of the extent to which mechanism or absolute MIC is more important in determining treatment success.

The pseudo-*R*^2^ values of 0.80–0.96 obtained suggest that our models explain most of the MIC variation. The residual, unexplained, variation probably relates to a combination of inherent phenotypic variability (which would not be addressed by a better model) and unidentified resistance determinants (or their interactions). Repeat testing of the same collection/subcollection of isolates would allow the former to be measured, and should ideally form part of future studies, to allow the latter to be estimated.

A limitation of the current modelling framework and sampling frames is that the available power allowed only determination of the effect of relatively common resistance determinants. For example, we did not have sufficient numbers of resistant isolates to formulate a similar model for ceftriaxone resistance. To avoid model over-fitting we selected the predictors in our final models based on the AIC; as such, it is possible that exclusion from the model of less common variants may be due to lack of power rather than lack of a true effect. An alternative approach would have been to retain all potential predictors identified *a priori* in the final model, albeit with an increased risk of model over-fitting.

Another related limitation is that the models are only as good as the resistant determinant catalogue on which they are based. Uncommon, but known, variants may have been excluded, as were likely multiple unknown resistance determinants. These unknown determinants have been referred to as ‘factor X’ in transformation experiments, where *penA*, *mtrR* and *penB* determinants increase resistance, but do confer donor levels of resistance to penicillin or extended-spectrum cephalosporins.^28^ Future WGS has the potential to identify novel resistance determinants through genome-wide association studies.^29^ The dataset used also restricts the resistance determinants for which coefficients can be determined. Several known resistance determinants were not present in our WGS dataset, and we were unable to determine their impact on MIC. One approach that might bridge this shortcoming would be to fit the model in a Bayesian framework where prior information from strains not previously subject to WGS, but with a known mechanism and MIC increment, e.g. from laboratory experiments with isogenic mutants, informs the final model as well as paired phenotypic and WGS data. There is also a need to derive models on more diverse datasets, e.g. many of the cefixime-resistant strains in this study are from a single clone dispersed throughout North America and England,^12^^,^^16^ and other mechanisms of cefixime resistance may be common elsewhere. Similarly, our approach of assigning each sequence to the most closely related *penA* allele grouping for cefixime and penicillin MIC prediction is only likely to be appropriate where similar alleles have been previously identified, and therefore sufficiently diverse derivation datasets are required to ensure the probability of encountering a very divergent novel allele is low.

The current study does not include an independent validation dataset, as we chose to include all available samples to maximize power. However, a further independent validation should be undertaken before applying our method to patient care.

WGS may become part of routine diagnostic microbiology workflows in the next 5–10 years.^30^ The approach taken in this study could be adopted, with sequencing following culture simultaneously providing information about antimicrobial susceptibilities and strain relatedness. Alternatively, if reliable inferences can be made from sequencing clinical samples directly this might offer further advantages, such as allowing culture-negative samples to be analysed and reducing turnaround times. However, further work will be required to develop methods to undertake WGS-based MIC prediction from clinical samples, including accounting for coexisting human and potentially similar commensal *Neisseria* species DNA.

In summary, WGS allows MIC prediction for a range of antimicrobials for *N. gonorrhoeae*. The approach taken here should allow reasonably precise prediction of MICs from genetic data for a range of bacterial species, at least to levels of variation considered acceptable for routine diagnostics. If WGS becomes a widely used diagnostic tool, large amounts of surveillance data may become available from routine clinical activity. WGS may therefore provide reproducible and readily exchangeable data on the spread of antimicrobial resistance, alongside providing informative data on the strains that are carrying it and how resistance is being transmitted.

## Data deposition

NCBI short-read archive accession numbers for sequences used in this publication and associated phenotypes and MIC predictions can be found in Table S2.

## Supplementary Material

Supplementary Data

## References

[1] Barbee LA. Preparing for an era of untreatable gonorrhoea. Curr Opin Infect Dis 2014; 27: 282–7. 24685549 10.1097/QCO.0000000000000058 PMC4097387

[2] PHE. *Surveillance of Antimicrobial Resistance in Neisseria gonorrhoeae: Key Findings from the ‘Gonococcal Resistance to Antimicrobials Surveillance Programme’ (GRASP) and Related Surveillance Data, 2014* 2015 https://www.gov.uk/government/uploads/system/uploads/attachment_data/file/476582/GRASP_2014_report_final_111115.pdf.

[3] ECDC. *Gonococcal Antimicrobial Susceptibility Surveillance in Europe 2014* 2016 http://ecdc.europa.eu/en/publications/Publications/gonococcal-antimicrobial-susceptibility-surveillance-Europe-2014.pdf.

[4] Kirkcaldy RD, Harvey A, Papp JR *Neisseria gonorrhoeae* Antimicrobial Susceptibility Surveillance—The Gonococcal Isolate Surveillance Project, 27 sites, United States, 2014. MMWR CDC Surveill Summ 2016; 65: 1–19. 10.15585/mmwr.ss6507a1 27414503

[5] Public Health Agency of Canada. *National Surveillance of Antimicrobial Susceptibilities of Neisseria gonorrhoeae Annual Summary 2014* 2015 http://healthycanadians.gc.ca/publications/drugs-products-medicaments-produits/2014-neisseria/alt/surveillance-gonorrhoeae-2014-eng.pdf.

[6] Demczuk W, Martin I, Peterson S Genomic epidemiology and molecular resistance mechanisms of azithromycin-resistant *Neisseria gonorrhoeae* in Canada from 1997 to 2014. J Clin Microbiol 2016; 54: 1304–13. 26935729 10.1128/JCM.03195-15 PMC4844716

[7] Grad YH, Harris SR, Kirkcaldy RD Genomic epidemiology of gonococcal resistance to extended spectrum cephalosporins, macrolides, and fluoroquinolones in the US, 2000-2013. J Infect Dis 2016; 214: 1579–87. 27638945 10.1093/infdis/jiw420 PMC5091375

[8] Gordon NC, Price JR, Cole K Prediction of *Staphylococcus aureus* antimicrobial resistance by whole-genome sequencing. J Clin Microbiol 2014; 52: 1182–91. 24501024 10.1128/JCM.03117-13 PMC3993491

[9] Stoesser N, Batty EM, Eyre DW Predicting antimicrobial susceptibilities for *Escherichia coli* and *Klebsiella pneumoniae* isolates using whole genomic sequence data. J Antimicrob Chemother 2013; 68: 2234–44. 23722448 10.1093/jac/dkt180 PMC3772739

[10] Walker TM, Kohl TA, Omar SV Whole-genome sequencing for prediction of *Mycobacterium tuberculosis* drug susceptibility and resistance: a retrospective cohort study. Lancet Infect Dis 2015; 15: 1193–202. 26116186 10.1016/S1473-3099(15)00062-6 PMC4579482

[11] Unemo M, Shafer WM. Antimicrobial resistance in *Neisseria gonorrhoeae* in the 21st century: past, evolution, and future. Clin Microbiol Rev 2014; 27: 587–613. 24982323 10.1128/CMR.00010-14 PMC4135894

[12] Grad YH, Kirkcaldy RD, Trees D Genomic epidemiology of *Neisseria gonorrhoeae* with reduced susceptibility to cefixime in the USA: a retrospective observational study. Lancet Infect Dis 2014; 14: 220–6. 24462211 10.1016/S1473-3099(13)70693-5 PMC4030102

[13] Unemo M, Golparian D, Sánchez-Busó L The novel 2016 WHO *Neisseria gonorrhoeae* reference strains for global quality assurance of laboratory investigations: phenotypic, genetic and reference genome characterization. J Antimicrob Chemother 2016; 71: 3096–108. 27432602 10.1093/jac/dkw288 PMC5079299

[14] Li Y, Metcalf BJ, Chochua S Penicillin-binding protein transpeptidase signatures for tracking and predicting β-lactam resistance levels in *Streptococcus pneumoniae*. MBio 2016; 7: e00756–16. 27302760 10.1128/mBio.00756-16 PMC4916381

[15] Metcalf BJ, Chochua S, Gertz RE Using whole genome sequencing to identify resistance determinants and predict antimicrobial resistance phenotypes for year 2015 invasive pneumococcal disease isolates recovered in the United States. Clin Microbiol Infect 2016; 22: 1002.e1–e8. 10.1016/j.cmi.2016.08.001 27542334

[16] De Silva D, Peters J, Cole K Whole-genome sequencing to determine transmission of *Neisseria gonorrhoeae*: an observational study. Lancet Infect Dis 2016; 16: 1295–303. 27427203 10.1016/S1473-3099(16)30157-8 PMC5086424

[17] Chisholm SA, Alexander S, Desouza-Thomas L Emergence of a *Neisseria gonorrhoeae* clone showing decreased susceptibility to cefixime in England and Wales. J Antimicrob Chemother 2011; 66: 2509–12. 21846672 10.1093/jac/dkr332

[18] CDC, US Department of Health and Human Services. *Gonococcal Isolate Surveillance Project (GISP) Protocol* http://www.cdc.gov/std/gisp/GISP-Protocol07-15-2010.pdf.

[19] Unemo M, Fasth O, Fredlund H Phenotypic and genetic characterization of the 2008 WHO *Neisseria gonorrhoeae* reference strain panel intended for global quality assurance and quality control of gonococcal antimicrobial resistance surveillance for public health purposes. J Antimicrob Chemother 2009; 63: 1142–51. 19318360 10.1093/jac/dkp098

[20] EUCAST. *Clinical Breakpoints—Bacteria (v 6.0)* 2016 http://www.eucast.org/fileadmin/src/media/PDFs/EUCAST_files/Breakpoint_tables/v_6.0_Breakpoint_table.pdf.

[21] Zerbino DR, Birney E. Velvet: algorithms for de novo short read assembly using de Bruijn graphs. Genome Res 2008; 18: 821–9. 18349386 10.1101/gr.074492.107 PMC2336801

[22] Edgar RC. MUSCLE: multiple sequence alignment with high accuracy and high throughput. Nucleic Acids Res 2004; 32: 1792–7. 15034147 10.1093/nar/gkh340 PMC390337

[23] Li H, Durbin R. Fast and accurate short read alignment with Burrows-Wheeler transform. Bioinformatics 2009; 25: 1754–60. 19451168 10.1093/bioinformatics/btp324 PMC2705234

[24] Li H, Handsaker B, Wysoker A The sequence Alignment/Map format and SAMtools. Bioinformatics 2009; 25: 2078–9. 19505943 10.1093/bioinformatics/btp352 PMC2723002

[25] Unemo M, Golparian D, Nicholas R High-level cefixime- and ceftriaxone-resistant *Neisseria gonorrhoeae* in France: novel *penA* mosaic allele in a successful international clone causes treatment failure. Antimicrob Agents Chemother 2012; 56: 1273–80. 22155830 10.1128/AAC.05760-11 PMC3294892

[26] US Department of Health and Human Services, FDA, Center for Devices and Radiological Health. *Class II Special Controls Guidance Document: Antimicrobial Susceptibility Test (AST) Systems* 2009 http://www.fda.gov/downloads/MedicalDevices/DeviceRegulationandGuidance/GuidanceDocuments/ucm071462.pdf.

[27] CDC, US Department of Health and Human Services. *Neisseria gonorrhoeae Reference Strains for Antimicrobial Susceptibility Testing* 2005 https://www.cdc.gov/std/gonorrhea/arg/b88-feb-2005.pdf.

[28] Unemo M, Nicholas RA. Emergence of multidrug-resistant, extensively drug-resistant and untreatable gonorrhea. Future Microbiol 2012; 7: 1401–22. 23231489 10.2217/fmb.12.117 PMC3629839

[29] Earle SG, Wu C-H, Charlesworth J Identifying lineage effects when controlling for population structure improves power in bacterial association studies. Nat Microbiol 2016; 1: 16041. 27572646 10.1038/nmicrobiol.2016.41 PMC5049680

[30] Didelot X, Bowden R, Wilson DJ Transforming clinical microbiology with bacterial genome sequencing. Nat Rev Genet 2012; 13: 601–12. 22868263 10.1038/nrg3226 PMC5049685

[31] Guindon S, Dufayard J-F, Lefort V New algorithms and methods to estimate maximum-likelihood phylogenies: assessing the performance of PhyML 3.0. Syst Biol 2010; 59: 307–21. 20525638 10.1093/sysbio/syq010

[32] Ohnishi M, Golparian D, Shimuta K Is *Neisseria gonorrhoeae* initiating a future era of untreatable gonorrhea?: Detailed characterization of the first strain with high-level resistance to ceftriaxone. Antimicrob Agents Chemother 2011; 55: 3538–45. 21576437 10.1128/AAC.00325-11 PMC3122416

[33] Bharat A, Demczuk W, Martin I Effect of variants of penicillin-binding protein 2 on cephalosporin and carbapenem susceptibilities in *Neisseria gonorrhoeae*. Antimicrob Agents Chemother 2015; 59: 5003–6. 25987627 10.1128/AAC.05143-14 PMC4505281

[34] Spratt BG. Hybrid penicillin-binding proteins in penicillin-resistant strains of *Neisseria gonorrhoeae*. Nature 1988; 332: 173–6. 3126399 10.1038/332173a0

[35] Cousin SL Jr, Whittington WLH, Roberts MC. Acquired macrolide resistance genes and the 1 bp deletion in the *mtrR* promoter in *Neisseria gonorrhoeae*. J Antimicrob Chemother 2003; 51: 131–3. 12493797 10.1093/jac/dkg040

[36] Zarantonelli L, Borthagaray G, Lee EH Decreased susceptibility to azithromycin and erythromycin mediated by a novel *mtr(R)* promoter mutation in *Neisseria gonorrhoeae*. J Antimicrob Chemother 2001; 47: 651–4. 11328778 10.1093/jac/47.5.651

[37] Jacobsson S, Golparian D, Cole M WGS analysis and molecular resistance mechanisms of azithromycin-resistant (MIC >2 mg/L) *Neisseria gonorrhoeae* isolates in Europe from 2009 to 2014. J Antimicrob Chemother 2016; 71: 3109–16. 27432597 10.1093/jac/dkw279

[38] Ohneck EA, Zalucki YM, Johnson PJT A novel mechanism of high-level, broad-spectrum antibiotic resistance caused by a single base pair change in *Neisseria gonorrhoeae*. MBio 2011; 2: e00187–11. 21933917 10.1128/mBio.00187-11 PMC3175627

[39] Warner DM, Shafer WM, Jerse AE. Clinically relevant mutations that cause derepression of the *Neisseria gonorrhoeae* MtrC‐MtrD‐MtrE efflux pump system confer different levels of antimicrobial resistance and in vivo fitness. Mol Microbiol 2008; 70: 462–78. 18761689 10.1111/j.1365-2958.2008.06424.x PMC2602950

[40] Olesky M, Hobbs M, Nicholas RA. Identification and analysis of amino acid mutations in porin IB that mediate intermediate-level resistance to penicillin and tetracycline in *Neisseria gonorrhoeae*. Antimicrob Agents Chemother 2002; 46: 2811–20. 12183233 10.1128/AAC.46.9.2811-2820.2002 PMC127413

[41] Zhao S, Duncan M, Tomberg J Genetics of chromosomally mediated intermediate resistance to ceftriaxone and cefixime in *Neisseria gonorrhoeae*. Antimicrob Agents Chemother 2009; 53: 3744–51. 19528266 10.1128/AAC.00304-09 PMC2737842

[42] Zhao S, Tobiason DM, Hu M The *penC* mutation conferring antibiotic resistance in *Neisseria gonorrhoeae* arises from a mutation in the PilQ secretin that interferes with multimer stability. Mol Microbiol 2005; 57: 1238–51. 16101998 10.1111/j.1365-2958.2005.04752.x PMC2673695

[43] Ohnishi M, Ono E, Shimuta K Identification of TEM-135 β-lactamase in penicillinase-producing *Neisseria gonorrhoeae* strains in Japan. Antimicrob Agents Chemother 2010; 54: 3021–3. 20421400 10.1128/AAC.00245-10 PMC2897271

[44] Palmer HM, Leeming JP, Turner A. A multiplex polymerase chain reaction to differentiate β-lactamase plasmids of *Neisseria gonorrhoeae*. J Antimicrob Chemother 2000; 45: 777–82. 10837429 10.1093/jac/45.6.777

[45] Chisholm SA, Dave J, Ison CA. High-level azithromycin resistance occurs in *Neisseria gonorrhoeae* as a result of a single point mutation in the 23S rRNA genes. Antimicrob Agents Chemother 2010; 54: 3812–6. 20585125 10.1128/AAC.00309-10 PMC2935028

[46] Ng L-K, Martin I, Liu G Mutation in 23S rRNA associated with macrolide resistance in *Neisseria gonorrhoeae*. Antimicrob Agents Chemother 2002; 46: 3020–5. 12183262 10.1128/AAC.46.9.3020-3025.2002 PMC127397

[47] Roberts MC, Chung WO, Roe D Erythromycin-resistant *Neisseria gonorrhoeae* and oral commensal *Neisseria* spp. carry known rRNA methylase genes. Antimicrob Agents Chemother 1999; 43: 1367–72. 10348754 10.1128/aac.43.6.1367 PMC89280

[48] Rouquette-Loughlin CE, Balthazar JT, Shafer WM. Characterization of the MacA-MacB efflux system in *Neisseria gonorrhoeae*. J Antimicrob Chemother 2005; 56: 856–60. 16162665 10.1093/jac/dki333

[49] Luna VA, Cousin S, Whittington WL Identification of the conjugative *mef* gene in clinical *Acinetobacter junii* and *Neisseria gonorrhoeae* isolates. Antimicrob Agents Chemother 2000; 44: 2503–6. 10952602 10.1128/aac.44.9.2503-2506.2000 PMC90092

[50] Belland RJ, Morrison SG, Ison C, Huang WM. *Neisseria gonorrhoeae* acquires mutations in analogous regions of *gyrA* and *parC* in fluoroquinolone-resistant isolates. Mol Microbiol 1994; 14: 371–80. 7830580 10.1111/j.1365-2958.1994.tb01297.x

[51] Rouquette-Loughlin C, Dunham SA, Kuhn M The NorM efflux pump of *Neisseria gonorrhoeae* and *Neisseria meningitidis* recognizes antimicrobial cationic compounds. J Bacteriol 2003; 185: 1101–6. 12533487 10.1128/JB.185.3.1101-1106.2003 PMC142806

[52] Hu M, Nandi S, Davies C High-level chromosomally mediated tetracycline resistance in *Neisseria gonorrhoeae* results from a point mutation in the *rpsJ* gene encoding ribosomal protein S10 in combination with the *mtrR* and *penB* resistance determinants. Antimicrob Agents Chemother 2005; 49: 4327–34. 16189114 10.1128/AAC.49.10.4327-4334.2005 PMC1251527

[53] Morse SA, Johnson SR, Biddle JW High-level tetracycline resistance in *Neisseria gonorrhoeae* is result of acquisition of streptococcal *tetM* determinant. Antimicrob Agents Chemother 1986; 30: 664–70. 3099640 10.1128/aac.30.5.664 PMC176510

[54] Gascoyne DM, Heritage J, Hawkey PM Molecular evolution of tetracycline-resistance plasmids carrying TetM found in *Neisseria gonorrhoeae* from different countries. J Antimicrob Chemother 1991; 28: 173–83. 1778850 10.1093/jac/28.2.173

