# Supplementary material for: WGS to predict antibiotic MICs for Neisseria gonorrhoeae
Source: J Antimicrob Chemother. 2017 Mar 10;72(7):1937–47. doi: 10.1093/jac/dkx067 (PMC5890716; doi:10.1093/jac/dkx067)
Supplement: Supplementary Data [file dkx067_supp.docx]

# **Supplementary data**

**Figure S1. Maximum likelihood phylogeny of *penA* alleles.** All previously published unique alleles and alleles available are Genbank are shown, after deduplication**.** Allele numbers are given where available, otherwise Genbank identifiers are used. Alleles found in our dataset are shown in red, alleles not found in grey. Alleles found in low frequency were grouped prior to analysis, the allele group is shown after the colon. Sequences were aligned using MUSCLE^22^ and the phylogeny constructed using phyML.^31^

**Figure S2. Distribution of cefixime minimum inhibitory concentrations, MICs, within fixed combinations of genetic determinants.** The numbers above each plot indicate an arbitrary allele identifier for the following genes/variables: *penA*, *penB* 120, *penB* 121, *ponA* 421, *mtrR* promoter disruption, *mtrR* 45, *mtrR* 39, *mtrR* truncation, mtr120, study country. Dashed lines represent clinical break-points.

**Figure S3. Distribution of penicillin minimum inhibitory concentrations, MICs, within fixed combinations of genetic determinants.** The numbers above each plot indicate an arbitrary allele identifier for the following genes/variables: *penA*, *penB* 120, *penB* 121, *ponA* 421, *bla_TEM_*, *mtrR* promoter disruption, *mtrR* 45, *mtrR* 39, *mtrR* truncation, *mtr120*, study country. Dashed lines represent clinical break-points

**Figure S4. Distribution of azithromycin minimum inhibitory concentrations, MICs, within fixed combinations of genetic determinants.** The numbers above each plot indicate an arbitrary allele identifier for the following genes/variables: rRNA A2059G copy number, rRNA C2611T copy number, *ermB* or *ermC*, *mtrR* promoter disruption, *mtrR* 45, *mtrR* 39, *mtrR* truncation, mtr120, study country. Dashed lines represent clinical break-points.

**Figure S5. Distribution of ciprofloxacin minimum inhibitory concentrations, MICs, within fixed combinations of genetic determinants.** The numbers above each plot indicate an arbitrary allele identifier for the following genes/variables: *gyrA* 91, *gyrA* 95, *parC* 86, *parC* 87, *parC* 91, study country. Dashed lines represent clinical break-points.

**Figure S6. Distribution of tetracycline minimum inhibitory concentrations, MICs, within fixed combinations of genetic determinants.** The numbers above each plot indicate an arbitrary allele identifier for the following genes/variables: *rpsJ* 57, *penB* 120, *tetM*, *mtrR* promoter disruption, *mtrR* 45, *mtrR* 39, *mtrR* truncation, mtr120, study country. Dashed lines represent clinical break-point

| Antibiotic | Predictor | Frequency (%) | Univariate coefficient | Univariate 95% confidence interval | Univariate p value | Multivariate coefficient | Multivariate 95% confidence interval | Multivariate p value |
| --- | --- | --- | --- | --- | --- | --- | --- | --- |
| Cefixime n=670 | Country: England | 245 (37%) | 0.00 |  |  | 0.00 |  |  |
|  | Country: Canada | 239 (36%) | 0.93 | (0.61, 1.24) | <0.001 | 0.60 | (0.42, 0.78) | <0.001 |
|  | Country: USA | 186 (28%) | 2.71 | (2.37, 3.05) | <0.001 | 1.36 | (1.18, 1.54) | <0.001 |
|  | *mtrR* 039 | 121 (18%) | -1.78 | (-2.17, -1.4) | <0.001 |  |  |  |
|  | *mtrR* 045 | 87 (13%) | -0.38 | (-0.85, 0.09) | 0.11 | 0.01 | (-0.27, 0.28) | 0.96 |
|  | mtr120 | 3 (0.4%) | 0.18 | (-2.18, 2.54) | 0.88 | 1.28 | (0.21, 2.35) | 0.019 |
|  | *mtrR* promoter: A-38C | 22 (3%) | 0.46 | (-0.3, 1.23) | 0.24 |  |  |  |
|  | *mtrR* promoter: deletion | 387 (58%) | 2.28 | (2.01, 2.56) | <0.001 |  |  |  |
|  | *mtrR* truncation | 44 (7%) | -1.44 | (-2.07, -0.81) | <0.001 |  |  |  |
|  | *penA*: M32091 | 13 (2%) | 0.00 |  |  | 0.00 |  |  |
|  | *penA*: II | 195 (29%) | 1.34 | (0.75, 1.94) | <0.001 | 0.65 | (0.15, 1.15) | 0.01 |
|  | *penA*: KP677512 | 12 (2%) | 3.38 | (2.54, 4.22) | <0.001 | 1.92 | (0.96, 2.88) | <0.001 |
|  | *penA*: KP721218 | 11 (2%) | 2.57 | (1.72, 3.43) | <0.001 | 2.01 | (1.25, 2.76) | <0.001 |
|  | *penA*: V | 46 (7%) | 2.31 | (1.66, 2.97) | <0.001 | 1.35 | (0.78, 1.91) | <0.001 |
|  | *penA*: X07470 | 47 (7%) | 2.14 | (1.48, 2.79) | <0.001 | 1.52 | (0.92, 2.12) | <0.001 |
|  | *penA*: XII | 51 (8%) | 2.47 | (1.82, 3.12) | <0.001 | 1.79 | (1.22, 2.37) | <0.001 |
|  | *penA*: XIX | 20 (3%) | 1.46 | (0.72, 2.21) | <0.001 | 0.96 | (0.36, 1.57) | 0.002 |
|  | *penA*: XV | 21 (3%) | -0.29 | (-1.02, 0.45) | 0.44 | -0.44 | (-1.04, 0.15) | 0.14 |
|  | *penA*: XXII | 45 (7%) | 1.19 | (0.53, 1.85) | <0.001 | 0.57 | (0.02, 1.13) | 0.042 |
|  | *penA*: XXXIV | 189 (28%) | 5.35 | (4.75, 5.95) | <0.001 | 3.08 | (2.19, 3.98) | <0.001 |
|  | *penA*: XXXV | 20 (3%) | 1.85 | (1.11, 2.60) | <0.001 | 0.89 | (0.24, 1.54) | 0.007 |
|  | *penA*: A501P | 1 (0.1%) | 7.20 |  | 0.001 | 4.74 | (3.05, 6.43) | <0.001 |
|  | *penA*: A501T | 14 (2%) | 0.68 |  | 0.22 | 0.97 | (0.21, 1.74) | 0.013 |
|  | *penA*: A501V | 10 (1%) | 1.18 |  | 0.073 | 0.96 | (0.35, 1.56) | 0.002 |
|  | *penA*: N512Y | 196 (29%) | 3.63 |  | <0.001 | 1.33 | (0.62, 2.04) | <0.001 |
|  | *penB* 120 | 369 (55%) | 2.36 | (2.10, 2.63) | <0.001 | 0.07 | (-0.19, 0.34) | 0.58 |
|  | *penB* 121 | 3 (0.4%) | -2.49 | (-4.84, -0.13) | 0.039 | -1.33 | (-2.39, -0.27) | 0.014 |
|  | *ponA* 421 | 413 (62%) | 2.17 | (1.89, 2.45) | <0.001 |  |  |  |
|  | Interaction: *mtrR* 045 + *penA*: II | 35 (5%) |  |  |  | 0.67 | (0.24, 1.1) | 0.002 |
|  | Interaction: *mtrR* 045 + Country: Canada | 28 (4%) |  |  |  | 0.72 | (0.27, 1.17) | 0.002 |
|  | Multivariate constant |  |  |  |  | -7.83 | (-8.29, -7.36) | <0.001 |
|  |  |  |  |  |  |  |  |  |
| Penicillin n=672 | Country: England | 247 (37%) | 0.00 |  |  | 0.00 |  |  |
|  | Country: Canada | 239 (36%) | 1.54 | (1.21, 1.88) | <0.001 | 1.33 | (1.14, 1.52) | <0.001 |
|  | Country: USA | 186 (28%) | 2.29 | (1.93, 2.64) | <0.001 | 1.57 | (1.37, 1.78) | <0.001 |
|  | *bla_TEM_* | 45 (7%) | 3.28 | (2.70, 3.87) | <0.001 | 4.55 | (3.80, 5.31) | <0.001 |
|  | *mtrR* 039 | 121 (18%) | -1.12 | (-1.53, -0.71) | <0.001 | 0.11 | (-0.19, 0.41) | 0.46 |
|  | *mtrR* 045 | 88 (13%) | -0.41 | (-0.88, 0.06) | 0.089 | 0.70 | (0.44, 0.96) | <0.001 |
|  | mtr120 | 3 (0.4%) | 0.69 | (-1.7, 3.08) | 0.57 | 0.98 | (-0.13, 2.09) | 0.085 |
|  | *mtrR* promoter: A-38C | 22 (3%) | 0.56 | (-0.23, 1.35) | 0.16 | 0.83 | (0.36, 1.31) | 0.001 |
|  | *mtrR* promoter: deletion | 388 (58%) | 2.22 | (1.93, 2.5) | <0.001 | 0.31 | (-0.07, 0.69) | 0.11 |
|  | *mtrR* truncation | 44 (7%) | -0.26 | (-0.9, 0.38) | 0.43 | -0.36 | (-0.72, -0.01) | 0.045 |
|  | *penA*: M32091 | 13 (2%) | 0.00 |  |  | 0.00 |  |  |
|  | *penA*: II | 195 (29%) | 2.56 | (1.67, 3.45) | <0.001 | -0.68 | (-1.92, 0.56) | 0.28 |
|  | *penA*: KP677512 | 12 (2%) | 3.30 | (2.05, 4.55) | <0.001 | -0.55 | (-1.98, 0.87) | 0.45 |
|  | *penA*: KP721218 | 11 (2%) | 4.71 | (3.43, 5.98) | <0.001 | 0.36 | (-1.09, 1.81) | 0.62 |
|  | *penA*: V | 46 (7%) | 4.67 | (3.69, 5.65) | <0.001 | 0.59 | (-0.77, 1.94) | 0.4 |
|  | *penA*: X07470 | 47 (7%) | 4.59 | (3.61, 5.57) | <0.001 | -0.61 | (-2.02, 0.79) | 0.39 |
|  | *penA*: XII | 51 (8%) | 4.53 | (3.56, 5.50) | <0.001 | 0.45 | (-0.91, 1.81) | 0.52 |
|  | *penA*: XIX | 20 (3%) | 5.34 | (4.22, 6.45) | <0.001 | -0.27 | (-1.67, 1.13) | 0.7 |
|  | *penA*: XV | 21 (3%) | -0.35 | (-1.45, 0.75) | 0.54 | -0.85 | (-1.5, -0.19) | 0.012 |
|  | *penA*: XXII | 46 (7%) | 1.83 | (0.85, 2.81) | <0.001 | -0.29 | (-1.54, 0.95) | 0.64 |
|  | *penA*: XXXIV | 190 (28%) | 4.66 | (3.76, 5.55) | <0.001 | 0.40 | (-0.94, 1.75) | 0.56 |
|  | *penA*: XXXV | 20 (3%) | 2.74 | (1.62, 3.85) | <0.001 | -0.34 | (-1.12, 0.44) | 0.39 |
|  | *penA*: F504L | 622 (93%) | 3.04 |  | <0.001 | 1.18 | (0.07, 2.29) | 0.037 |
|  | *penB* 120 | 370 (55%) | 2.44 | (2.18, 2.7) | <0.001 | 1.00 | (0.74, 1.26) | <0.001 |
|  | *penB* 121 | 3 (0.4%) | -0.99 | (-3.37, 1.4) | 0.42 |  |  |  |
|  | *ponA* 421 | 414 (62%) | 2.56 | (2.29, 2.82) | <0.001 | 0.74 | (0.41, 1.07) | <0.001 |
|  | Interaction: *bla_TEM_* + *mtrR* 39 | 25 (4%) |  |  |  | 1.50 |  | <0.001 |
|  | Interaction: *bla_TEM_* + *mtrR* promoter: deletion | 11 (2%) |  |  |  | 2.42 |  | <0.001 |
|  | Interaction: *bla_TEM_* + *penA*: XXXIV | 3 (0.4%) |  |  |  | -4.72 | (-6.06, -3.38) | <0.001 |
|  | Interaction: *bla_TEM_* + *ponA* 421 | 32 (5%) |  |  |  | -1.67 | (-2.41, -0.92) | <0.001 |
|  | Interaction: *bla_TEM_* + Country: USA | 13 (2%) |  |  |  | -1.26 |  | 0.001 |
|  | Interaction: *penA*: X07470 + Country Canada | 34 (5%) |  |  |  | 1.06 | (0.42, 1.71) | 0.001 |
|  | Multivariate constant |  |  |  |  | -3.87 | (-4.38, -3.36) | <0.001 |
|  |  |  |  |  |  |  |  |  |
| Azithromycin n=681 | Country: England | 249 (37%) | 0.00 |  |  | 0.00 |  |  |
|  | Country: Canada | 246 (36%) | 4.36 | (4.08, 4.64) | <0.001 | 2.28 | (2.04, 2.52) | <0.001 |
|  | Country: USA | 186 (27%) | 1.76 | (1.46, 2.07) | <0.001 | 1.74 | (1.39, 2.08) | <0.001 |
|  | *ermB* or *ermC* | 3 (0.4%) | 2.34 | (-0.07, 4.75) | 0.057 | 4.48 | (2.61, 6.35) | <0.001 |
|  | *mtrR* 039 | 124 (18%) | -0.04 | (-0.52, 0.44) | 0.88 | 0.37 | (0.14, 0.60) | 0.002 |
|  | *mtrR* 045 | 89 (13%) | -0.41 | (-0.96, 0.14) | 0.14 |  |  |  |
|  | mtr120 | 3 (0.4%) | 0.99 | (-1.79, 3.78) | 0.48 |  |  |  |
|  | *mtrR* promoter: A-38C | 22 (3%) | 2.12 | (1.07, 3.18) | <0.001 | 1.97 | (1.50, 2.45) | <0.001 |
|  | *mtrR* promoter: deletion | 393 (58%) | 0.48 | (0.10, 0.86) | 0.012 | 1.31 | (1.07, 1.55) | <0.001 |
|  | *mtrR* truncation | 44 (6%) | -0.22 | (-0.97, 0.53) | 0.56 | -0.97 | (-1.27, -0.66) | <0.001 |
|  | rRNA A2059G: 4 copies | 5 (1%) | 9.39 | (7.35, 11.43) | <0.001 | 8.55 | (7.69, 9.40) | <0.001 |
|  | rRNA C2611T: per copy [range 0-4] | 0 copies: 519 (76%); 1 copy: 2 (0.3%); 2 copies: 11 (2%); 3 copies: 13 (2%); 4 copies: 136 (20%) | 1.08 | (1, 1.16) | <0.001 | 1.52 | (1.25, 1.79) | <0.001 |
|  | Interaction: *ermB* or *ermC* + *mtrR* promoter: deletion | 3 (0.4%) |  |  |  | -3.60 | (-5.75, -1.46) | 0.001 |
|  | Interaction: *mtrR* promoter: deletion + rRNA C2611T per copy | 63 (9%) |  |  |  | -0.25 | (-0.34, -0.15) | <0.001 |
|  | Interaction: *mtrR* promoter: deletion + Country: USA | 137 (20%) |  |  |  | -0.77 | (-1.16, -0.37) | <0.001 |
|  | Interaction: rRNA C2611T per copy + Country: Canada | 141 (21%) |  |  |  | -0.60 | (-0.87, -0.32) | <0.001 |
|  | Interaction: rRNA C2611T per copy + Country: USA | 17 (2%) |  |  |  | -0.45 | (-0.75, -0.14) | 0.004 |
|  | Multivariate constant |  |  |  |  | -3.13 | (-3.32, -2.94) | <0.001 |
|  |  |  |  |  |  |  |  |  |
| Ciprofloxacin n=676 | Country: England | 249 (37%) | 0.00 |  |  | 0.00 |  |  |
|  | Country: Canada | 241 (36%) | -1.27 | (-2.11, -0.43) | 0.003 | -0.14 | (-0.31, 0.04) | 0.12 |
|  | Country: USA | 186 (28%) | 2.07 | (1.17, 2.96) | <0.001 | -0.44 | (-0.63, -0.25) | <0.001 |
|  | *gyrA* 91 | 374 (55%) | 9.58 | (9.41, 9.75) | <0.001 | 2.89 | (1.81, 3.98) | <0.001 |
|  | *gyrA* 95 | 371 (55%) | 9.60 | (9.45, 9.76) | <0.001 | 4.07 | (2.86, 5.29) | <0.001 |
|  | *parC* 86 | 18 (3%) | 4.19 | (1.91, 6.46) | <0.001 | 2.31 | (1.61, 3.01) | <0.001 |
|  | *parC* 87 | 326 (48%) | 8.62 | (8.26, 8.97) | <0.001 | 2.90 | (2.34, 3.45) | <0.001 |
|  | *parC* 91 | 20 (3%) | 4.10 | (1.94, 6.26) | <0.001 | 2.30 | (1.57, 3.03) | <0.001 |
|  | Interaction: *parC* 87 + *parC* 91 | 5 (1%) |  |  | . | -2.96 | (-4.08, -1.84) | <0.001 |
|  | Multivariate constant |  |  |  | . | -5.80 | (-5.95, -5.66) | <0.001 |
|  |  |  |  |  |  |  |  |  |
| Tetracycline n=681 | Country: England | 249 (37%) |  |  |  |  |  |  |
|  | Country: Canada | 246 (36%) | -0.60 | (-0.87, -0.32) | <0.001 | -0.77 | (-0.91, -0.63) | <0.001 |
|  | Country: USA | 186 (27%) | -0.96 | (-1.26, -0.66) | <0.001 | -1.42 | (-1.56, -1.27) | <0.001 |
|  | *mtrR* 039 | 124 (18%) | 0.03 | (-0.28, 0.35) | 0.84 |  |  |  |
|  | *mtrR* 045 | 89 (13%) | -0.39 | (-0.75, -0.03) | 0.032 | -0.07 | (-0.25, 0.11) | 0.45 |
|  | mtr120 | 3 (0.4%) | 0.76 | (-1.08, 2.59) | 0.42 |  |  |  |
|  | *mtrR* promoter: A-38C | 22 (3%) | 0.09 | (-0.59, 0.77) | 0.80 | 0.66 | (0.34, 0.99) | <0.001 |
|  | *mtrR* promoter: deletion | 393 (58%) | 0.83 | (0.59, 1.08) | <0.001 | 0.17 | (-0.07, 0.41) | 0.16 |
|  | *mtrR* truncation | 44 (6%) | 0.30 | (-0.19, 0.8) | 0.23 | -0.39 | (-0.64, -0.15) | 0.002 |
|  | *penB* 120 | 375 (55%) | 1.18 | (0.95, 1.4) | <0.001 | 0.72 | (0.42, 1.02) | <0.001 |
|  | *rpsJ* 57 | 638 (94%) | 2.21 | (1.73, 2.68) | <0.001 | 2.66 | (2.39, 2.92) | <0.001 |
|  | *tetM* | 53 (8%) | 3.81 | (3.46, 4.16) | <0.001 | 6.98 | (6.36, 7.60) | <0.001 |
|  | Interaction: *mtrR* 045 + *tetM* | 8 (1%) |  |  |  | -0.90 | (-1.48, -0.31) | 0.003 |
|  | Interaction: *mtrR* promoter: deletion + *penB* 120 | 349 (51%) |  |  |  | 0.55 | (0.17, 0.92) | 0.005 |
|  | Interaction: *mtrR* promoter: deletion + *tetM* | 11 (2%) |  |  |  | -1.02 | (-1.53, -0.51) | <0.001 |
|  | Interaction *rpsJ* 57 + *tetM* | 47 (7%) |  |  |  | -2.69 | (-3.36, -2.02) | <0.001 |
|  | Multivariate constant |  |  |  |  | -1.34 | (-1.57, -1.11) | <0.001 |

**Table S1. Univariate and multivariate predictors of minimum inhibitory concentration.**

Comparisons for genetic determinants are with the wild-type allele. *penA* comparisons are made with M32091. Country comparisons are relative to England. rRNA mutations are compared to samples with 0 copies of the mutation. There were no *parC* S88P or *pilQ* E666K mutations, no *norM* or MacAB promoter mutations, and no *erm*(F), *ere*, *mef* genes identified. Point mutation sites with a novel amino acid were classed as non-wild-type. The *penA* alleles listed indicate the presence of that specific allele or a closely related variant (see methods). *penA* genotypes could not be determined for 2 samples, these samples were excluded from the cefixime and penicillin models. A further 9 and 7 samples were excluded from the cefixime and pencillin models respectively as the *penA* SNPs could not be determined due to incomplete *de novo* assemblies. *parC* variants could not be determined for 5 samples, these samples were excluded from the ciprofloxacin model. For brevity only individual *penA* SNPs that were included in the multivariate model in addition to the *penA* allele are shown. rRNA A2059G was present in zero or four copies only.

| **Study** | **Oxford GUID** | **NCBI identifier** | **Sample name** | **Cefixime** | | **Penicillin** | | **Azithromycin** | | **Ciprofloxacin** | | **Tetracycline** | |
| --- | --- | --- | --- | --- | --- | --- | --- | --- | --- | --- | --- | --- | --- |
|  |  |  |  | **Phenotype** | **Prediction** | **Phenotype** | **Prediction** | **Phenotype** | **Prediction** | **Phenotype** | **Prediction** | **Phenotype** | **Prediction** |
| Brighton, England | 003224b2-4e68-4bb4-aefe-87c924393b76 | SRR3361307 | Gr09-1211 | 0.008 | 0.008 | 0.250 | 0.125 | 0.500 | 0.125 | 0.015 | 0.016 | 4.000 | 2.000 |
| Brighton, England | 00e7e328-505d-4c58-8e3e-0c2d12a53b00 | SRR3361308 | Gr08-1772 | 0.004 | 0.004 | 0.060 | 0.063 | 0.060 | 0.125 | 0.015 | 0.016 | 0.250 | 0.500 |
| Brighton, England | 025faa10-c6e0-448a-b048-909173298f29 | SRR3357011 | GC8251 | 0.125 | 0.125 | 1.000 | 1.000 | 0.500 | 0.250 | 32.000 | 16.000 | 8.000 | 8.000 |
| Brighton, England | 036fdc8b-f013-448d-b938-22c764f26f21 | SRR3361310 | Gr10-2238 | 0.250 | 0.125 | 1.000 | 1.000 | 0.500 | 0.250 | 32.000 | 16.000 | 8.000 | 8.000 |
| Brighton, England | 039bc69f-d5ae-4c17-a678-f2af105a3531 | SRR3361311 | Gr10-1636 | 0.125 | 0.125 | 1.000 | 1.000 | 0.500 | 0.250 | 16.000 | 16.000 | 8.000 | 8.000 |
| Brighton, England | 0414697e-66f2-47cf-bb19-86610b5359a7 | SRR3361312 | Gr10-2189 | 0.125 | 0.125 | 1.000 | 1.000 | 0.500 | 0.250 | 32.000 | 16.000 | 8.000 | 8.000 |
| Brighton, England | 04339651-a7bf-452b-85b1-274b51cbaf1a | SRR3349516 | GC11251 |  |  |  |  | 0.500 | 0.250 | 32.000 | 16.000 | 8.000 | 8.000 |
| Brighton, England | 04e4affa-fa4c-43da-bd53-24d3701088c3 | SRR3361313 | Gr10-1645 | 0.008 | 0.004 | 0.060 | 0.063 | 0.030 | 0.125 | 0.030 | 0.016 | 0.250 | 0.500 |
| Brighton, England | 052d7cc3-ed68-4c0b-a923-97b9d6f4a923 | SRR3361314 | Gr10-2192 | 0.015 | 0.016 | 2.000 | 1.000 | 0.250 | 0.250 | 32.000 | 16.000 | 8.000 | 8.000 |
| Brighton, England | 059d1be8-9f63-4367-8160-0f2a5c29e344 | SRR3361315 | Gr10-2215 | 0.015 | 0.008 | 0.060 | 0.250 | 0.125 | 0.125 | 0.030 | 0.016 | 16.000 | 32.000 |
| Brighton, England | 074d31dd-6e0f-4afd-a21d-26b7c38f0f02 | SRR3349518 | GC11240 | 0.008 | 0.008 | 0.250 | 0.250 | 0.250 | 0.250 | 8.000 | 16.000 | 32.000 | 16.000 |
| Brighton, England | 08511034-ab92-4efb-9a09-601c3b8af0ec | SRR3360913 | Gr05-0375 | 0.008 | 0.008 | 0.060 | 0.125 | 0.125 | 0.125 | 0.015 | 0.016 | 2.000 | 2.000 |
| Brighton, England | 0be9fd83-c224-4a1d-b7d2-312132d977f0 | SRR3361317 | Gr07-1002 | 0.030 | 0.016 | 2.000 | 1.000 | 1.000 | 0.250 | 0.500 | 16.000 | 8.000 | 8.000 |
| Brighton, England | 0c0c35d4-1ebb-4fd5-861a-4ef4d263a405 | SRR3361318 | Gr10-2220 | 0.004 | 0.004 | 0.060 | 0.063 | 0.060 | 0.125 | 0.030 | 0.016 | 0.250 | 0.500 |
| Brighton, England | 0c7e8031-6b20-411d-a034-c547a76d65b6 | SRR3361320 | Gr08-2086 | 0.002 | 0.004 | 0.030 | 0.031 | 0.250 | 0.125 | 0.015 | 0.016 | 0.250 | 0.500 |
| Brighton, England | 0e1c1ed2-bf1c-46a1-b56b-c20fe850b9ec | SRR3361321 | Gr07-1613 | 0.125 | 0.125 | 0.500 | 1.000 | 0.500 | 0.250 | 32.000 | 16.000 | 4.000 | 8.000 |
| Brighton, England | 0e1e753a-b232-404b-8aba-1b78baa751b4 | SRR3360917 | Gr04-0012 | 0.008 | 0.008 | 1.000 | 0.500 | 0.125 | 0.125 | 0.015 | 0.016 | 4.000 | 4.000 |
| Brighton, England | 0ebb465b-1e30-49f0-a346-f8ac02d689c5 | SRR3361322 | Gr09-0569 | 0.008 | 0.004 | 0.250 | 0.125 | 0.060 | 0.125 | 0.015 | 0.016 | 4.000 | 2.000 |
| Brighton, England | 0f52063a-6f6e-4304-9d6e-dbd491da6743 | SRR3349526 | GC11242 | 0.015 | 0.016 | 0.250 | 0.250 | 0.250 | 0.125 | 0.015 | 0.016 | 4.000 | 4.000 |
| Brighton, England | 10229367-ff5e-4741-b97e-bbe304ff9d6a | SRR3360921 | Gr04-0029 | 0.008 | 0.008 | 0.060 | 0.125 | 0.500 | 0.125 | 0.015 | 0.016 | 4.000 | 2.000 |
| Brighton, England | 12da8295-2984-45c8-9908-ce8b3d91527d | SRR3361324 | Gr07-1103 | 0.008 | 0.016 | 0.125 | 1.000 | 0.030 | 0.250 | 16.000 | 16.000 | 2.000 | 8.000 |
| Brighton, England | 13a11f58-bc3a-468d-85d4-616676dc7504 | SRR3360924 | Gr05-0378 | 0.015 | 0.008 | 0.250 | 0.250 | 0.030 | 0.250 | 0.015 | 0.016 | 1.000 | 2.000 |
| Brighton, England | 15926d1a-232c-4901-9b85-ab7adb656ecd | SRR3361325 | Gr10-1566 | 0.015 | 0.125 | 1.000 | 1.000 | 0.500 | 0.250 | 32.000 | 16.000 | 8.000 | 8.000 |
| Brighton, England | 16162c35-261f-4578-b19b-fcf94606d128 | SRR3361326 | Gr07-0417 | 0.004 | 0.008 | 0.060 | 0.125 | 0.125 | 0.125 | 0.015 | 0.016 | 8.000 | 2.000 |
| Brighton, England | 1620de89-d5e1-49fc-9ac9-87710bb4aa97 | SRR3360926 | Gr04-0003 | 0.015 | 0.016 | 0.250 | 0.500 | 0.125 | 0.250 | 0.030 | 0.016 | 4.000 | 8.000 |
| Brighton, England | 1935886b-6283-47cb-ad77-e1c330db1c79 | SRR3349538 | GC11279 | 0.125 | 0.125 | 1.000 | 1.000 | 0.500 | 0.250 | 32.000 | 16.000 | 8.000 | 8.000 |
| Brighton, England | 19a167a4-a4ac-4c76-a926-29d8d502a44b | SRR3360930 | Gr05-1671 | 0.015 | 0.016 | 0.500 | 1.000 | 0.250 | 0.250 | 32.000 | 16.000 | 4.000 | 8.000 |
| Brighton, England | 1b87ae4a-8f70-4906-b4bc-f48897cf0eef | SRR3361328 | Gr10-1646 | 0.008 | 0.008 | 0.500 | 0.250 | 0.125 | 0.125 | 0.030 | 0.016 | 16.000 | 32.000 |
| Brighton, England | 1bf97ace-7a6d-4b79-b729-c53e5ac082a5 | SRR3361329 | Gr09-0443 |  |  |  |  | 0.250 | 0.250 | 32.000 | 16.000 | 4.000 | 8.000 |
| Brighton, England | 1ce3dfb6-c947-4dca-898b-a249ae38edab | SRR3361332 | Gr07-1637 | 0.015 | 0.016 | 1.000 | 0.500 | 0.250 | 0.250 | 0.015 | 0.016 | 2.000 | 8.000 |
| Brighton, England | 1e23e143-123b-4cba-99d8-9c87b4475087 | SRR3361333 | Gr07-0418 | 0.004 | 0.008 | 0.250 | 0.250 | 0.250 | 0.125 | 0.015 | 0.016 | 2.000 | 2.000 |
| Brighton, England | 1ef66b9f-546b-4e8b-a701-4cf3b0018a11 | SRR3361336 | Gr07-1644 | 0.015 | 0.016 | 1.000 | 1.000 | 0.250 | 0.250 | 32.000 | 16.000 | 4.000 | 8.000 |
| Brighton, England | 1feef958-9428-4733-bf28-7807169bb023 | SRR3361337 | Gr10-1565 | 0.125 | 0.125 | 0.500 | 1.000 | 0.250 | 0.250 | 32.000 | 16.000 | 8.000 | 8.000 |
| Brighton, England | 204f1c6f-b4a6-41a1-b9ce-b1fd9d9f69a9 | SRR3357028 | GC8286 | 0.125 | 0.125 | 1.000 | 1.000 | 0.250 | 0.250 | 32.000 | 16.000 | 8.000 | 8.000 |
| Brighton, England | 20ccd869-1444-45a2-aecf-f2b36b3cb07f | SRR3360936 | Gr04-0027 | 0.008 | 0.008 | 0.030 | 0.125 | 0.500 | 0.125 | 0.015 | 0.016 | 64.000 | 64.000 |
| Brighton, England | 224e3bd9-e53a-477d-9104-071485a68812 | SRR3349544 | GC11274 | 0.008 | 0.004 | 0.125 | 0.063 | 0.060 | 0.125 | 0.015 | 0.016 | 0.250 | 0.500 |
| Brighton, England | 23d200d6-7b04-4c24-8245-7b32201bff03 | SRR3361338 | Gr10-2227 | 0.002 | 0.004 | 0.125 | 0.500 | 0.125 | 0.250 | 0.030 | 0.016 | 2.000 | 2.000 |
| Brighton, England | 24ea3692-c371-4ad9-bf08-d0f09fcab6a0 | SRR3360939 | Gr04-0014 | 0.008 | 0.008 | 0.125 | 0.250 | 0.125 | 0.125 | 0.015 | 0.016 | 0.500 | 0.500 |
| Brighton, England | 27364a4e-0b10-4283-b1cd-d55c9ce6e5d5 | SRR3361340 | Gr09-0592 | 0.004 | 0.008 | 0.250 | 0.125 | 0.125 | 0.125 | 0.015 | 0.016 | 4.000 | 2.000 |
| Brighton, England | 27611501-a936-4973-8731-1a879758ee71 | SRR3361341 | Gr10-1619 | 0.008 | 0.008 | 8.000 | 8.000 | 0.030 | 0.063 | 4.000 | 16.000 | 32.000 | 64.000 |
| Brighton, England | 27bc7c41-5243-4882-83c2-b78d6847ba0a | SRR3361342 | Gr09-1210 | 0.008 | 0.008 | 0.250 | 0.250 | 0.500 | 0.250 | 32.000 | 16.000 | 8.000 | 8.000 |
| Brighton, England | 27eb90fc-88ed-4336-ac7b-1fd51be23ab8 | SRR3349546 | GC11272 | 0.004 | 0.016 | 8.000 | 8.000 | 0.060 | 0.125 | 8.000 | 16.000 | 32.000 | 32.000 |
| Brighton, England | 28657e1e-c826-4518-97ba-64024851755b | SRR3361343 | Gr10-2194 | 0.002 | 0.004 | 0.060 | 0.031 | 0.060 | 0.125 | 0.030 | 0.016 | 0.250 | 0.500 |
| Brighton, England | 28833192-94ff-4024-8b19-dbdae2f81568 | SRR3361344 | Gr10-2208 | 0.125 | 0.125 | 1.000 | 1.000 | 0.250 | 0.250 | 16.000 | 16.000 | 8.000 | 8.000 |
| Brighton, England | 2a2df14c-3f66-476f-bb51-bf043907edd1 | SRR3361345 | Gr08-1027 | 0.002 | 0.004 | 0.030 | 0.031 | 0.125 | 0.125 | 0.015 | 0.016 | 0.500 | 0.500 |
| Brighton, England | 2ae77bb4-5c75-4676-b35e-436c0e218544 | SRR3361346 | Gr08-2505 | 0.004 | 0.016 | 0.250 | 0.500 | 0.250 | 0.250 | 8.000 | 16.000 | 8.000 | 8.000 |
| Brighton, England | 2afd47e0-ddb1-4c5d-8163-61bd0304fb71 | SRR3361347 | Gr10-1081 | 0.125 | 0.125 | 1.000 | 1.000 | 0.250 | 0.250 | 32.000 | 16.000 | 8.000 | 8.000 |
| Brighton, England | 2cf780f2-1653-4d5f-9dd2-46d99160d4da | SRR3360943 | Gr05-0892 | 0.004 | 0.004 | 0.060 | 0.031 | 0.500 | 0.125 | 0.015 | 0.016 | 0.500 | 0.500 |
| Brighton, England | 2d23aaae-c65a-490f-ad3c-56f392957e4e | SRR3360944 | Gr04-0024 | 0.002 | 0.016 | 0.500 | 0.500 | 0.500 | 0.250 | 4.000 | 16.000 | 2.000 | 4.000 |
| Brighton, England | 2d7206f5-2a5b-4f28-a8f0-f3ce01aeae37 | SRR3361349 | Gr07-1107 | 0.008 | 0.008 | 0.250 | 0.125 | 0.250 | 0.125 | 0.015 | 0.016 | 4.000 | 2.000 |
| Brighton, England | 2dd79201-f23d-4c0e-98f3-76a1c596ef08 | SRR3361350 | Gr09-1195 | 0.002 | 0.004 | 0.030 | 0.031 | 0.125 | 0.125 | 0.015 | 0.016 | 0.250 | 0.500 |
| Brighton, England | 2ddbb8de-bc4c-44a0-b8d7-196dbdb0b965 | SRR3361351 | Gr08-0996 | 0.015 | 0.016 | 0.500 | 0.500 | 0.250 | 0.250 | 16.000 | 16.000 | 4.000 | 2.000 |
| Brighton, England | 2e349b82-958a-4557-9219-dbd18acf3a3f | SRR3349554 | GC11256 | 0.004 | 0.008 | 0.125 | 0.125 | 0.125 | 0.125 | 0.015 | 0.016 | 4.000 | 2.000 |
| Brighton, England | 2ebab9e0-5a44-44f2-b449-355bc5827930 | SRR3357077 | GC8255 | 0.004 | 0.008 | 0.125 | 0.125 | 0.125 | 0.125 | 0.015 | 0.016 | 2.000 | 2.000 |
| Brighton, England | 2f79c788-db00-49d9-8153-afd2f1e6f077 | SRR3360947 | Gr04-0013 | 0.002 | 0.004 | 0.030 | 0.031 | 0.125 | 0.125 | 0.015 | 0.016 | 0.250 | 0.500 |
| Brighton, England | 301b6cd9-0e59-4e5d-bda0-4b6b0622d22b | SRR3349557 | GC11229 | 0.008 | 0.008 | 0.125 | 0.125 | 1.000 | 0.125 | 0.015 | 0.016 | 4.000 | 2.000 |
| Brighton, England | 3036986e-368a-4abc-8f34-90d1d624ed01 | SRR3361352 | Gr07-1657 | 0.004 | 0.008 | 0.125 | 0.125 | 0.125 | 0.125 | 0.015 | 0.016 | 4.000 | 2.000 |
| Brighton, England | 31650b70-71b2-4204-8945-3a0d8f5b338b | SRR3360949 | Gr05-0374 | 0.030 | 0.016 | 1.000 | 1.000 | 0.250 | 0.250 | 32.000 | 16.000 | 8.000 | 8.000 |
| Brighton, England | 31bce801-9f2a-438f-852a-900c1439ddac | SRR3361353 | Gr05-1686 | 0.015 | 0.008 | 0.250 | 0.125 | 0.250 | 0.125 | 0.015 | 0.016 | 2.000 | 2.000 |
| Brighton, England | 3270e849-9966-4e0f-9d27-bc452dba62ec | SRR3361354 | Gr10-1648 | 0.250 | 0.125 | 1.000 | 1.000 | 0.500 | 0.250 | 32.000 | 16.000 | 8.000 | 8.000 |
| Brighton, England | 33e91e5f-acb4-4116-8d12-13489e28ab65 | SRR3361355 | Gr08-2660 | 0.004 | 0.008 | 0.500 | 0.125 | 0.125 | 0.125 | 0.015 | 0.016 | 4.000 | 2.000 |
| Brighton, England | 342582b9-eb99-4c3b-8b8e-8e3a280c7d1b | SRR3361356 | Gr08-2085 | 0.008 | 0.016 | 0.500 | 1.000 | 0.250 | 0.250 | 32.000 | 16.000 | 4.000 | 8.000 |
| Brighton, England | 34e5f8b5-31b6-43c4-aeb7-dde06f77ec0e | SRR3360605 | Gr07-1672 | 0.015 | 0.016 | 1.000 | 1.000 | 0.500 | 0.250 | 32.000 | 16.000 | 8.000 | 8.000 |
| Brighton, England | 34eff39e-34af-466b-9676-0b44bdcde1c3 | SRR3360606 | Gr07-1669 | 0.008 | 0.008 | 2.000 | 4.000 | 0.125 | 0.125 | 2.000 | 2.000 | 64.000 | 64.000 |
| Brighton, England | 35782d00-f5d2-4f09-88af-eb578f1820db | SRR3360950 | Gr04-0062 | 0.004 | 0.008 | 0.060 | 0.125 | 0.125 | 0.125 | 0.015 | 0.016 | 2.000 | 2.000 |
| Brighton, England | 3593478b-05fd-41bd-bcb8-b768eaca3b2c | SRR3349563 | GC11220 | 0.015 | 0.008 | 0.125 | 0.125 | 0.125 | 0.125 | 0.015 | 0.016 | 32.000 | 64.000 |
| Brighton, England | 3810a27e-669a-43c3-bd45-223836c7ae61 | SRR3360607 | Gr07-1671 | 0.008 | 0.016 | 0.500 | 0.500 | 0.250 | 0.250 | 1.000 | 16.000 | 2.000 | 4.000 |
| Brighton, England | 38bb8e5b-b89d-4e4e-8ca9-1d2650fdb450 | SRR3360953 | Gr05-1682 | 0.030 | 0.008 | 0.500 | 0.125 | 0.250 | 0.125 | 0.015 | 0.016 | 2.000 | 2.000 |
| Brighton, England | 38d2b514-9d6f-475d-919c-241e7b753af3 | SRR3360608 | Gr09-1252 | 0.004 | 0.004 | 0.125 | 0.063 | 0.125 | 0.125 | 0.015 | 0.016 | 0.500 | 0.500 |
| Brighton, England | 3afd7a8e-6790-45a6-97f7-69c1a13a756c | SRR3360609 | Gr08-2658 | 0.015 | 0.016 | 0.500 | 0.250 | 0.125 | 0.125 | 0.015 | 0.016 | 4.000 | 2.000 |
| Brighton, England | 3c8c1feb-3466-4019-bc4e-4b023be6b75d | SRR3360610 | Gr09-1271 | 0.015 | 0.016 | 1.000 | 1.000 | 0.125 | 0.250 | 32.000 | 16.000 | 4.000 | 8.000 |
| Brighton, England | 3d235c33-96d8-415c-be99-c29c8d1681d9 | SRR3360612 | Gr10-2193 | 0.125 | 0.125 | 1.000 | 1.000 | 0.250 | 0.250 | 16.000 | 16.000 | 8.000 | 8.000 |
| Brighton, England | 3d48f53f-7f0f-4747-91dc-771689569a1f | SRR3360613 | Gr07-0364 | 0.004 | 0.008 | 0.250 | 0.125 | 0.250 | 0.125 | 0.015 | 0.016 | 4.000 | 2.000 |
| Brighton, England | 3d5ae094-a2f4-4a80-8ebc-2dd9e3c6d47d | SRR3360614 | Gr10-1635 | 0.060 | 0.031 | 0.500 | 0.500 | 0.250 | 0.250 | 16.000 | 8.000 | 8.000 | 8.000 |
| Brighton, England | 3d9c0060-b582-426a-8785-cb9207a2d94d | SRR3349568 | GC11280 | 0.008 | 0.016 | 8.000 | 8.000 | 0.060 | 0.125 | 8.000 | 16.000 | 32.000 | 32.000 |
| Brighton, England | 3f5627d2-118f-491a-a6cd-0d9b3d734e36 | SRR3360615 | Gr08-1469 | 0.008 | 0.016 | 8.000 | 4.000 | 0.125 | 0.063 | 4.000 | 16.000 | 64.000 | 64.000 |
| Brighton, England | 3f97b84c-051c-4005-8b1f-e4554dbf64cf | SRR3349573 | GC11296 | 0.015 | 0.016 | 0.500 | 0.500 | 0.250 | 0.250 | 16.000 | 16.000 | 4.000 | 2.000 |
| Brighton, England | 41372e4f-dff3-4892-9cf4-18ac756b9dab | SRR3360616 | Gr09-1253 | 0.125 | 0.125 | 1.000 | 1.000 | 0.500 | 0.250 | 32.000 | 16.000 | 8.000 | 8.000 |
| Brighton, England | 445fd893-4fd5-4e77-8499-221b11a0d674 | SRR3360617 | Gr09-0032 | 0.015 | 0.008 | 0.250 | 0.250 | 0.500 | 0.250 | 32.000 | 16.000 | 4.000 | 8.000 |
| Brighton, England | 456ec686-f97a-4224-8286-f46f1e488b2a | SRR3360618 | Gr09-1196 | 0.008 | 0.016 | 0.500 | 0.500 | 0.500 | 0.250 | 8.000 | 16.000 | 4.000 | 2.000 |
| Brighton, England | 465877aa-c929-4a65-8ede-e12f96fc84fa | SRR3360619 | Gr09-0580 | 0.125 | 0.125 | 0.500 | 1.000 | 0.500 | 0.250 | 32.000 | 16.000 | 8.000 | 8.000 |
| Brighton, England | 4c8850cb-0e45-4e4d-a422-c2a9a1d34457 | SRR3349577 | GC11278 | 0.004 | 0.008 | 8.000 | 8.000 | 0.125 | 0.125 | 0.015 | 0.016 | 2.000 | 2.000 |
| Brighton, England | 4ce2d596-fd8c-4c2f-8f66-04f84c4e4b52 | SRR3360622 | Gr09-0102 | 0.015 | 0.008 | 0.250 | 0.250 | 0.500 | 0.250 | 8.000 | 16.000 | 4.000 | 8.000 |
| Brighton, England | 4d796d28-870f-4381-9257-b3b297e4b474 | SRR3360964 | Gr04-0023 | 0.015 | 0.008 | 0.060 | 0.125 | 0.125 | 0.125 | 0.015 | 0.016 | 2.000 | 2.000 |
| Brighton, England | 4ff4fd30-4491-4489-8fa3-b454186b6f7d | SRR3360624 | Gr05-1685 | 0.002 | 0.008 | 1.000 | 8.000 | 0.125 | 0.063 | 2.000 | 16.000 | 64.000 | 64.000 |
| Brighton, England | 507cc787-f979-400b-8e4c-bc302672d58f | SRR3360625 | Gr07-0314 | 0.008 | 0.008 | 2.000 | 4.000 | 0.125 | 0.125 | 2.000 | 2.000 | 64.000 | 64.000 |
| Brighton, England | 53fecbfc-5338-485b-bf8c-b8dae960f722 | SRR3360967 | Gr04-0020 | 0.004 | 0.008 | 0.060 | 0.125 | 0.060 | 0.125 | 0.015 | 0.016 | 64.000 | 64.000 |
| Brighton, England | 5619fa73-e814-4dc7-af5a-d7284dc710be | SRR3360970 | Gr04-0057 | 0.008 | 0.016 | 0.250 | 0.500 | 0.125 | 0.250 | 8.000 | 16.000 | 2.000 | 8.000 |
| Brighton, England | 57be32a5-7a41-43b2-8140-015341dee293 | SRR3360627 | Gr07-1612 | 0.004 | 0.016 | 0.250 | 0.500 | 0.500 | 0.250 | 8.000 | 16.000 | 4.000 | 2.000 |
| Brighton, England | 590bfa43-d3fe-443a-8967-a508a1612594 | SRR3360628 | Gr10-2190 | 0.125 | 0.125 | 0.500 | 1.000 | 0.250 | 0.250 | 16.000 | 16.000 | 8.000 | 8.000 |
| Brighton, England | 599d7449-b6d0-4a2a-a5bd-5c2601f52879 | SRR3357157 | GC8259 | 0.125 | 0.125 | 2.000 | 0.500 | 0.250 | 0.125 | 32.000 | 16.000 | 8.000 | 4.000 |
| Brighton, England | 59b7956f-72c7-40d5-a449-6ec61f4fec08 | SRR3360629 | Gr10-1118 | 0.008 | 0.008 | 4.000 | 1.000 | 0.125 | 0.125 | 8.000 | 16.000 | 4.000 | 2.000 |
| Brighton, England | 5a9c227e-e300-4358-94e6-dd3a3fec1a47 | SRR3360630 | Gr09-0565 | 0.004 | 0.008 | 0.060 | 0.125 | 0.030 | 0.125 | 0.015 | 0.016 | 0.250 | 0.500 |
| Brighton, England | 5b18db60-5406-483a-b5a0-844b6887d09b | SRR3360632 | Gr09-1197 | 0.008 | 0.016 | 0.500 | 0.500 | 0.500 | 0.250 | 8.000 | 16.000 | 4.000 | 2.000 |
| Brighton, England | 5bef97c0-5d21-44fb-9934-bd461e6ab9e3 | SRR3360980 | Gr05-0380B | 0.008 | 0.016 | 0.250 | 0.500 | 0.250 | 0.250 | 8.000 | 16.000 | 8.000 | 8.000 |
| Brighton, England | 5cad1d89-1b4a-4090-87dc-c004f956eff5 | SRR3360633 | Gr10-1541 | 0.125 | 0.125 | 1.000 | 1.000 | 0.500 | 0.250 | 32.000 | 16.000 | 8.000 | 8.000 |
| Brighton, England | 5cf5441b-5af6-47f3-b0a9-4ae84068b97f | SRR3360634 | Gr10-1120 | 0.008 | 0.008 | 4.000 | 1.000 | 0.125 | 0.125 | 8.000 | 16.000 | 4.000 | 2.000 |
| Brighton, England | 5d460430-12c9-408b-a148-0c1e46fe68e1 | SRR3357160 | GC8266 | 0.004 | 0.016 | 0.030 | 0.250 | 0.125 | 0.125 | 0.015 | 0.016 | 2.000 | 4.000 |
| Brighton, England | 5d7207b8-4fac-4399-a24d-5a5105a144a2 | SRR3360981 | Gr04-0010 | 0.002 | 0.016 | 0.060 | 0.500 | 0.030 | 0.250 | 0.015 | 0.016 | 2.000 | 8.000 |
| Brighton, England | 5e1f839c-7aa7-4f6b-a16a-e1cceadbf46e | SRR3360982 | Gr04-0068 | 0.008 | 0.008 | 0.125 | 0.250 | 0.030 | 0.063 | 0.015 | 0.016 | 64.000 | 32.000 |
| Brighton, England | 5eb9f187-7661-4a16-bb6c-bb8c98c86a04 | SRR3360983 | Gr04-0065 | 0.008 | 0.008 | 0.250 | 0.250 | 0.500 | 0.250 | 8.000 | 16.000 | 4.000 | 2.000 |
| Brighton, England | 5fd5840e-a793-40d2-a00d-ce61e60ee76a | SRR3360635 | Gr09-1213 | 0.004 | 0.008 | 8.000 | 8.000 | 0.125 | 0.125 | 0.015 | 0.016 | 2.000 | 2.000 |
| Brighton, England | 615a06db-4e08-44c7-8b98-2558da0b52e6 | SRR3360984 | Gr04-0037 | 0.008 | 0.008 | 0.060 | 0.250 | 0.500 | 0.250 | 8.000 | 16.000 | 2.000 | 2.000 |
| Brighton, England | 622c4f0d-f108-4294-bd3d-28bb27be8d5f | SRR3360636 | Gr08-2084 | 0.002 | 0.008 | 0.030 | 0.125 | 0.030 | 0.063 | 0.015 | 0.016 | 0.250 | 0.250 |
| Brighton, England | 6645a23a-2e48-4916-9800-5d7b24d32dff | SRR3360637 | Gr07-1623 | 0.008 | 0.016 | 0.500 | 0.500 | 0.250 | 0.250 | 16.000 | 8.000 | 4.000 | 8.000 |
| Brighton, England | 66ce46a2-c798-455e-9dc2-a85ef649d801 | SRR3360638 | Gr08-2359 | 0.008 | 0.008 | 0.250 | 0.125 | 0.125 | 0.125 | 0.015 | 0.016 | 4.000 | 2.000 |
| Brighton, England | 67006e07-dc5d-42c0-b58a-24d412b7900e | SRR3360639 | Gr08-2504 | 0.015 | 0.016 | 0.500 | 0.500 | 0.250 | 0.250 | 16.000 | 16.000 | 4.000 | 2.000 |
| Brighton, England | 684a1cef-59c9-4b26-84d4-2fe1e325e76c | SRR3360991 | Gr04-0026 | 0.008 | 0.008 | 0.060 | 0.125 | 0.060 | 0.125 | 0.015 | 0.016 | 64.000 | 64.000 |
| Brighton, England | 6853dddb-f730-4957-8b82-17e538f4b0f1 | SRR3360640 | Gr10-1031 | 0.008 | 0.008 | 0.250 | 0.125 | 1.000 | 0.500 | 0.030 | 0.016 | 8.000 | 4.000 |
| Brighton, England | 695815ac-c8f4-4334-96c2-2ef7fe0e896a | SRR3360641 | Gr10-1644 | 0.030 | 0.031 | 0.500 | 0.500 | 0.250 | 0.250 | 16.000 | 8.000 | 8.000 | 8.000 |
| Brighton, England | 6aed6545-efa5-4486-9152-1e201b4617db | SRR3360642 | Gr10-1078 | 0.008 | 0.008 | 0.250 | 0.125 | 0.250 | 0.125 | 0.030 | 0.016 | 4.000 | 2.000 |
| Brighton, England | 6b1e3145-be07-4ace-a1e0-0f55c9f8fc15 | SRR3360993 | Gr04-0016 | 0.004 | 0.008 | 0.125 | 0.125 | 0.060 | 0.125 | 0.015 | 0.016 | 2.000 | 2.000 |
| Brighton, England | 6bb9e4e4-f998-46b6-a2c0-7149c876bc58 | SRR3360644 | Gr09-0648 | 0.004 | 0.008 | 8.000 | 2.000 | 0.125 | 0.125 | 8.000 | 16.000 | 64.000 | 64.000 |
| Brighton, England | 6c3f5d0d-ea6d-4468-885c-fc6a943376c0 | SRR3360645 | Gr10-1049 | 0.125 | 0.125 | 1.000 | 1.000 | 0.500 | 0.250 | 32.000 | 16.000 | 8.000 | 8.000 |
| Brighton, England | 6ec797df-e76c-4c31-8d4d-414874610174 | SRR3360646 | Gr07-0429 | 0.008 | 0.008 | 0.250 | 0.250 | 0.125 | 0.250 | 0.015 | 0.016 | 1.000 | 4.000 |
| Brighton, England | 6f29476c-f8b4-4526-91c7-8db4ba4d9a4a | SRR3360647 | Gr10-1097 | 0.004 | 0.004 | 0.060 | 0.063 | 0.060 | 0.125 | 0.030 | 0.016 | 32.000 | 64.000 |
| Brighton, England | 6f3f1236-15d8-4184-952f-ca7ca30fa0b6 | SRR3360648 | Gr09-0099 | 0.125 | 0.125 | 0.500 | 1.000 | 0.500 | 0.250 | 32.000 | 16.000 | 4.000 | 8.000 |
| Brighton, England | 714dbc36-e3d5-4bfe-a831-60f293d7df14 | SRR3360649 | Gr08-2255 | 0.015 | 0.016 | 0.500 | 0.500 | 0.250 | 0.250 | 16.000 | 16.000 | 4.000 | 2.000 |
| Brighton, England | 717b3ee1-04e3-4585-a28a-4fcb47b82ea9 | SRR3360650 | Gr10-1621 | 0.008 | 0.008 | 0.250 | 0.125 | 0.250 | 0.125 | 0.030 | 0.016 | 4.000 | 2.000 |
| Brighton, England | 718b61a0-b0ae-4f33-aeac-d369a42b93a2 | SRR3360651 | Gr10-1618 | 0.008 | 0.008 | 8.000 | 8.000 | 0.030 | 0.063 | 4.000 | 16.000 | 32.000 | 64.000 |
| Brighton, England | 733d84ce-3a7b-4067-8b5b-2723b8b1275a | SRR3349601 | GC11287 | 0.060 | 0.031 | 0.250 | 0.500 | 0.125 | 0.250 | 16.000 | 8.000 | 8.000 | 8.000 |
| Brighton, England | 737ab7f3-9bee-4772-aa47-a3686f0967f4 | SRR3360652 | Gr09-0031 | 0.015 | 0.008 | 4.000 | 4.000 | 0.060 | 0.125 | 4.000 | 16.000 | 64.000 | 64.000 |
| Brighton, England | 73cebb15-5ed9-406c-ab72-7f54fd0892d1 | SRR3360766 | Gr05-0893 | 0.004 | 0.004 | 0.030 | 0.031 | 0.500 | 0.125 | 0.015 | 0.016 | 0.250 | 0.500 |
| Brighton, England | 73dc46c8-ea4e-4193-9546-211ba516a169 | SRR3360653 | Gr07-1638 | 0.015 | 0.016 | 0.500 | 0.500 | 0.250 | 0.250 | 0.015 | 0.016 | 2.000 | 8.000 |
| Brighton, England | 74f7c181-ff49-4689-be18-02d41ff4a9e6 | SRR3360654 | Gr07-1120 | 0.004 | 0.004 | 0.125 | 0.063 | 0.060 | 0.125 | 0.015 | 0.016 | 0.250 | 0.500 |
| Brighton, England | 7620aff7-930b-4cbb-85cf-01781ed06bff | SRR3360771 | Gr05-0368 | 0.015 | 0.008 | 0.125 | 0.125 | 0.060 | 0.125 | 0.015 | 0.016 | 2.000 | 2.000 |
| Brighton, England | 7713fb91-739c-45a6-9408-a5e3f8307ca9 | SRR3360772 | Gr04-0015 | 0.008 | 0.016 | 0.500 | 0.500 | 0.125 | 0.250 | 8.000 | 16.000 | 4.000 | 8.000 |
| Brighton, England | 78f54357-effb-4d4d-a658-6f9d3874e3d9 | SRR3360774 | Gr04-0050 | 0.008 | 0.008 | 0.500 | 0.250 | 0.250 | 0.250 | 8.000 | 16.000 | 4.000 | 2.000 |
| Brighton, England | 79047a1b-86b6-4d0c-9dc3-a442a494128f | SRR3357181 | GC8256 | 0.125 | 0.125 | 1.000 | 1.000 | 0.500 | 0.250 | 32.000 | 16.000 | 8.000 | 8.000 |
| Brighton, England | 7d5aad71-0bfc-42e8-b17e-6e8eb6a35ec5 | SRR3360658 | Gr10-2219 | 0.125 | 0.125 | 0.500 | 1.000 | 0.250 | 0.250 | 16.000 | 16.000 | 8.000 | 8.000 |
| Brighton, England | 7dc769c5-ae98-4ce6-b01d-ea5ac4c06cf6 | SRR3360659 | Gr09-0643 | 0.125 | 0.125 | 0.500 | 1.000 | 0.250 | 0.250 | 32.000 | 16.000 | 8.000 | 8.000 |
| Brighton, England | 7fd977ee-5182-49b7-97dd-e2503eb39c57 | SRR3357186 | GC8268 | 0.060 | 0.125 | 0.250 | 1.000 | 0.015 | 0.250 | 8.000 | 16.000 | 4.000 | 8.000 |
| Brighton, England | 801016ff-95c9-4a9b-a1b3-2ba5218181fe | SRR3360810 | Gr04-0005 | 0.030 | 0.016 | 1.000 | 0.500 | 0.125 | 0.250 | 0.015 | 0.016 | 4.000 | 8.000 |
| Brighton, England | 80e9e586-d275-47d8-9606-8f4934186445 | SRR3360811 | Gr05-0369 | 0.004 | 0.004 | 0.030 | 0.031 | 0.250 | 0.125 | 0.015 | 0.016 | 0.250 | 0.500 |
| Brighton, England | 816c1ab0-19f2-42d2-a0e4-1965446e69d8 | SRR3360661 | Gr08-2508 | 0.002 | 0.008 | 2.000 | 8.000 | 0.125 | 0.125 | 0.015 | 0.016 | 4.000 | 2.000 |
| Brighton, England | 8192bb86-1369-4276-8787-d827e49d7b5f | SRR3360662 | Gr09-0126 | 0.002 | 0.008 | 0.060 | 0.125 | 0.030 | 0.125 | 0.015 | 0.016 | 2.000 | 2.000 |
| Brighton, England | 81a1600c-2976-4976-9072-ee7f262ddf80 | SRR3349615 | GC11289 | 0.004 | 0.016 | 0.125 | 0.500 | 0.125 | 0.250 | 16.000 | 8.000 | 8.000 | 8.000 |
| Brighton, England | 81e02ede-99b3-46f4-9738-a942f19a939c | SRR3360813 | Gr05-0370 | 0.030 | 0.016 | 1.000 | 1.000 | 0.125 | 0.250 | 32.000 | 16.000 | 8.000 | 8.000 |
| Brighton, England | 8274f622-e2e6-4fcc-8284-a6c364866e16 | SRR3360663 | Gr08-2657 | 0.015 | 0.004 | 0.500 | 0.031 | 0.250 | 0.125 | 0.015 | 0.016 | 0.250 | 0.500 |
| Brighton, England | 82faeda7-599f-471d-964e-2b0be933a4a5 | SRR3360664 | Gr07-1180 | 0.015 | 0.016 | 2.000 | 1.000 | 0.250 | 0.250 | 32.000 | 16.000 | 8.000 | 8.000 |
| Brighton, England | 82ff63ba-bd10-473f-8010-b3caa87aa76b | SRR3360665 | Gr09-0112 | 0.002 | 0.004 | 0.030 | 0.063 | 0.030 | 0.125 | 0.015 | 0.016 | 1.000 | 0.500 |
| Brighton, England | 84988b42-ed65-4745-8211-dd62d87728c1 | SRR3360667 | Gr10-1059 | 0.008 | 0.016 | 0.250 | 0.500 | 0.250 | 0.250 | 32.000 | 8.000 | 8.000 | 8.000 |
| Brighton, England | 86a10a6d-3405-4ff7-a5e8-c68a1143a82a | SRR3360669 | Gr08-1138 | 0.002 | 0.031 | 0.030 | 0.250 | 0.060 | 0.125 | 0.015 | 0.016 | 1.000 | 2.000 |
| Brighton, England | 86d9f87d-30f2-411d-9aa9-458c924e40af | SRR3360670 | Gr08-1137 | 0.008 | 0.016 | 0.500 | 0.250 | 0.500 | 0.125 | 0.015 | 0.016 | 4.000 | 2.000 |
| Brighton, England | 88895eec-121f-492c-9b1f-b83334072aad | SRR3360671 | Gr07-0317 | 0.015 | 0.016 | 2.000 | 1.000 | 0.500 | 0.250 | 32.000 | 16.000 | 8.000 | 8.000 |
| Brighton, England | 89d9f578-f50c-47d5-a120-b9b0e79ac1fd | SRR3357191 | GC8205 | 0.250 | 0.125 | 1.000 | 1.000 | 0.500 | 0.250 | 32.000 | 16.000 | 8.000 | 8.000 |
| Brighton, England | 8a7acd60-0b8e-4038-bcbb-f8278a7d2108 | SRR3360672 | Gr07-1104 | 0.008 | 0.008 | 8.000 | 4.000 | 0.125 | 0.063 | 2.000 | 16.000 | 64.000 | 32.000 |
| Brighton, England | 8acc5366-0c75-464b-be9b-87fdb1e058b9 | SRR3357192 | GC8298 | 0.125 | 0.125 | 2.000 | 1.000 | 0.500 | 0.250 | 32.000 | 16.000 | 8.000 | 8.000 |
| Brighton, England | 8e681fe9-bb3e-4dfc-839c-b9445e9537c4 | SRR3360674 | Gr09-1272 | 0.030 | 0.125 | 0.125 | 1.000 | 0.030 | 0.250 | 8.000 | 16.000 | 2.000 | 8.000 |
| Brighton, England | 8f8d2ae6-4b23-48e6-8e7d-5f42d9831ac5 | SRR3360827 | Gr05-0889 | 0.008 | 0.004 | 0.060 | 0.031 | 0.500 | 0.125 | 0.015 | 0.016 | 0.250 | 0.500 |
| Brighton, England | 91fd37d8-d6b1-417f-9d2b-65556312cfbe | SRR3360829 | Gr04-0032 | 0.015 | 0.008 | 0.125 | 0.125 | 0.125 | 0.125 | 0.015 | 0.016 | 2.000 | 2.000 |
| Brighton, England | 926863d7-56b1-4557-829e-4045156d0218 | SRR3357194 | GC8269 | 0.060 | 0.125 | 0.250 | 1.000 | 0.060 | 0.250 | 8.000 | 16.000 | 4.000 | 8.000 |
| Brighton, England | 928b9ea6-82e0-49a0-8e2c-8f789350830c | SRR3360675 | Gr07-1178 | 0.015 | 0.016 | 2.000 | 1.000 | 0.500 | 0.250 | 32.000 | 16.000 | 8.000 | 8.000 |
| Brighton, England | 94d223fe-394b-49b9-ab5a-55b7cd32b9e0 | SRR3360832 | Gr05-0885 | 0.008 | 0.008 | 0.125 | 0.063 | 0.250 | 0.063 | 0.015 | 0.016 | 2.000 | 2.000 |
| Brighton, England | 985f80da-73c4-42a7-a99e-cb21ca7194ef | SRR3360677 | Gr09-0645 | 0.002 | 0.004 | 0.030 | 0.063 | 0.125 | 0.250 | 0.015 | 0.016 | 2.000 | 0.500 |
| Brighton, England | 986e6cbe-09c2-4b16-8f6d-25d12e08167f | SRR3360678 | Gr07-1144 | 0.008 | 0.008 | 8.000 | 4.000 | 0.250 | 0.125 | 4.000 | 2.000 | 64.000 | 64.000 |
| Brighton, England | 98889bed-6648-4b64-ac2f-802118d5ade1 | SRR3360679 | Gr10-1082 | 0.004 | 0.008 | 2.000 | 8.000 | 0.125 | 0.125 | 0.030 | 0.016 | 2.000 | 2.000 |
| Brighton, England | 9bb8b4b9-d033-4bae-b505-e3883474769f | SRR3360680 | Gr10-2209 | 0.060 | 0.031 | 0.500 | 0.500 | 0.250 | 0.250 | 16.000 | 8.000 | 8.000 | 8.000 |
| Brighton, England | 9c4612e8-7cae-42aa-a7e7-8ee33e967996 | SRR3360681 | Gr05-1687 | 0.004 | 0.004 | 0.250 | 0.063 | 0.500 | 0.250 | 8.000 | 16.000 | 2.000 | 4.000 |
| Brighton, England | 9e3a2db6-8b27-4485-9974-27bfd8935954 | SRR3360836 | Gr04-0064 | 0.004 | 0.008 | 0.060 | 0.125 | 0.125 | 0.125 | 0.015 | 0.016 | 64.000 | 64.000 |
| Brighton, England | 9e872dd0-93bf-410f-8639-c73fb70c8755 | SRR3360683 | Gr10-2191 | 0.002 | 0.004 | 0.060 | 0.031 | 0.060 | 0.125 | 0.030 | 0.016 | 0.250 | 0.500 |
| Brighton, England | 9e8b4aa1-fae5-40ef-a79b-1dce06434c5a | SRR3360684 | Gr08-1470 | 0.008 | 0.016 | 0.500 | 0.500 | 0.250 | 0.250 | 8.000 | 16.000 | 4.000 | 2.000 |
| Brighton, England | a20ce48f-6b1c-4a41-a351-5eb669a2d0b3 | SRR3360685 | Gr07-1629 | 0.002 | 0.008 | 0.250 | 0.125 | 0.125 | 0.125 | 0.015 | 0.016 | 2.000 | 2.000 |
| Brighton, England | a246fc51-3508-4d99-82fc-23634851b7b9 | SRR3360686 | Gr10-1622 | 0.250 | 0.125 | 1.000 | 1.000 | 0.500 | 0.250 | 32.000 | 16.000 | 8.000 | 8.000 |
| Brighton, England | a479a20d-c6ce-4880-baab-8d0355c30e4c | SRR3360687 | Gr07-1620 | 0.002 | 0.004 | 0.030 | 0.031 | 0.125 | 0.125 | 0.015 | 0.016 | 1.000 | 0.500 |
| Brighton, England | a4a54667-3cb0-4bb9-b064-166ece1ba2d1 | SRR3360688 | Gr08-1471 | 0.008 | 0.008 | 0.250 | 0.125 | 8.000 | 8.000 | 0.015 | 0.016 | 2.000 | 2.000 |
| Brighton, England | a5b8d741-7cfb-411e-8d59-cd5a824dc6f4 | SRR3357229 | GC8302 | 0.030 | 0.016 | 0.250 | 0.250 | 0.125 | 0.125 | 0.015 | 0.016 | 2.000 | 2.000 |
| Brighton, England | a716206a-25b1-41e7-82c9-34149d6b0ffb | SRR3360689 | Gr09-0571 | 0.004 | 0.008 | 0.125 | 0.125 | 0.250 | 0.125 | 0.015 | 0.016 | 4.000 | 2.000 |
| Brighton, England | a87ee0af-d9b5-4f0e-babb-6c7ba6907a97 | SRR3357246 | GC8253 | 0.030 | 0.016 | 0.125 | 0.125 | 0.125 | 0.125 | 0.015 | 0.016 | 1.000 | 0.500 |
| Brighton, England | ab949a09-8365-4dbd-a636-622665554cc5 | SRR3360690 | Gr09-0577 | 0.004 | 0.008 | 2.000 | 8.000 | 0.125 | 0.125 | 0.015 | 0.016 | 2.000 | 2.000 |
| Brighton, England | acf0a94f-0ba2-4d4d-96b3-ab2373628b0f | SRR3360691 | Gr07-0396 | 0.015 | 0.008 | 8.000 | 4.000 | 0.125 | 0.125 | 2.000 | 2.000 | 64.000 | 64.000 |
| Brighton, England | ad547171-57e8-4411-9931-03e369708386 | SRR3360692 | Gr09-0101 | 0.015 | 0.008 | 0.125 | 0.063 | 0.125 | 0.063 | 0.015 | 0.016 | 2.000 | 2.000 |
| Brighton, England | ad7708c4-9c0b-4e97-937f-1028be88bb5f | SRR3360693 | Gr09-1200 |  |  | 0.500 | 1.000 | 0.500 | 0.250 | 16.000 | 16.000 | 8.000 | 8.000 |
| Brighton, England | afb073dd-c8a7-47f4-a066-9bc7dda1bae5 | SRR3360846 | Gr05-1675 | 0.015 | 0.016 | 0.500 | 0.500 | 0.500 | 0.250 | 32.000 | 8.000 | 4.000 | 8.000 |
| Brighton, England | afcc166f-045a-49ac-b9fb-49fe6cf5f8f9 | SRR3360694 | Gr07-1106 | 0.015 | 0.016 | 0.500 | 0.500 | 0.250 | 0.250 | 16.000 | 16.000 | 4.000 | 2.000 |
| Brighton, England | b2658bd3-519d-4598-8fef-a24f35baf9ae | SRR3360696 | Gr10-1055 | 0.004 | 0.008 | 0.125 | 0.125 | 0.125 | 0.125 | 0.030 | 0.016 | 2.000 | 2.000 |
| Brighton, England | b2896225-8e21-46bf-9cae-29438d17cf0a | SRR3360697 | Gr07-1621 | 0.008 | 0.008 | 8.000 | 4.000 | 0.125 | 0.125 | 2.000 | 2.000 | 64.000 | 64.000 |
| Brighton, England | b4a33027-e05c-4cc8-85b1-fd345ea8ceda | SRR3357252 | GC8240 | 0.125 | 0.125 | 1.000 | 1.000 | 0.500 | 0.250 | 32.000 | 16.000 | 8.000 | 8.000 |
| Brighton, England | b7d05d5e-5c93-48ac-9e58-301a97f95327 | SRR3360698 | Gr10-1060 | 0.030 | 0.031 | 0.250 | 0.500 | 0.250 | 0.250 | 16.000 | 8.000 | 8.000 | 8.000 |
| Brighton, England | ba3c95dd-1bd4-4d21-87c5-a3870bff70ad | SRR3349658 | GC11216 | 0.008 | 0.016 | 0.125 | 0.250 | 0.250 | 0.125 | 0.015 | 0.016 | 4.000 | 4.000 |
| Brighton, England | ba55626d-c126-4a0d-9d4c-1cd7d6426f5a | SRR3360700 | Gr07-1670 | 0.008 | 0.016 | 0.500 | 0.500 | 0.250 | 0.250 | 1.000 | 16.000 | 2.000 | 4.000 |
| Brighton, England | ba848349-bde1-47b6-925c-f2bd33736a8a | SRR3360851 | Gr05-0376 | 0.004 | 0.004 | 0.030 | 0.063 | 0.125 | 0.250 | 8.000 | 16.000 | 2.000 | 4.000 |
| Brighton, England | bad4486b-21ca-4008-9f34-f8638dbc944c | SRR3360701 | Gr07-0313 | 0.004 | 0.008 | 0.500 | 0.125 | 0.250 | 0.125 | 0.015 | 0.016 | 4.000 | 2.000 |
| Brighton, England | bb9c9160-ef0d-41fc-b97d-aa7578662d8b | SRR3360702 | Gr10-1681 | 0.125 | 0.125 | 0.500 | 1.000 | 0.250 | 0.250 | 32.000 | 16.000 | 8.000 | 8.000 |
| Brighton, England | bcaa537a-379b-4ed3-92a4-5760d9ceed03 | SRR3360703 | Gr07-0428 | 0.008 | 0.016 | 1.000 | 1.000 | 0.500 | 0.250 | 16.000 | 16.000 | 4.000 | 8.000 |
| Brighton, England | bda7aca0-5de8-4874-bf04-6feb2997b4d7 | SRR3360854 | Gr05-0367 | 0.015 | 0.004 | 0.060 | 0.063 | 0.030 | 0.125 | 0.015 | 0.016 | 0.500 | 0.500 |
| Brighton, England | be63989e-4d5f-4a23-8d27-0bd6bb761077 | SRR3360704 | Gr07-1658 | 0.015 | 0.016 | 0.125 | 0.500 | 0.250 | 0.250 | 16.000 | 16.000 | 2.000 | 4.000 |
| Brighton, England | be7964aa-100e-42b4-8b2f-c22ade1ce369 | SRR3360705 | Gr08-2659 | 0.002 | 0.004 | 0.030 | 0.031 | 0.060 | 0.125 | 0.015 | 0.016 | 0.250 | 0.500 |
| Brighton, England | bed23283-d088-41db-b64b-2167aa7fea34 | SRR3357260 | GC8261 | 0.008 | 0.016 | 0.125 | 0.125 | 0.125 | 0.125 | 0.015 | 0.016 | 1.000 | 0.500 |
| Brighton, England | bf6060d1-0394-4dd4-9189-719cbc3dbff5 | SRR3360706 | Gr10-1658 | 0.008 | 0.008 | 0.250 | 0.125 | 0.125 | 0.125 | 0.030 | 0.016 | 4.000 | 2.000 |
| Brighton, England | c168da0e-2368-4935-9117-0b8b695b6538 | SRR3360707 | Gr10-1050 | 0.125 | 0.125 | 1.000 | 1.000 | 0.500 | 0.250 | 32.000 | 16.000 | 8.000 | 8.000 |
| Brighton, England | c16a825f-5eb8-41b3-b6c7-0d0a09057e17 | SRR3360708 | Gr10-1623 | 0.004 | 0.008 | 0.125 | 0.125 | 8.000 | 8.000 | 0.030 | 0.016 | 4.000 | 2.000 |
| Brighton, England | c1a34f92-30b5-4b9a-8042-7ada04951e74 | SRR3357263 | GC8267 | 0.125 | 0.125 | 1.000 | 1.000 | 0.500 | 0.250 | 32.000 | 16.000 | 8.000 | 8.000 |
| Brighton, England | c24b791e-c9c6-4d0b-9caa-eeba42de6041 | SRR3360709 | Gr10-1624 | 0.125 | 0.125 | 1.000 | 1.000 | 0.500 | 0.250 | 32.000 | 16.000 | 8.000 | 8.000 |
| Brighton, England | c2d3240d-bda4-4b89-82f7-9d199f60fbdc | SRR3360710 | Gr10-2188 | 0.008 | 0.008 | 0.125 | 0.125 | 0.250 | 0.125 | 0.030 | 0.016 | 4.000 | 2.000 |
| Brighton, England | c32f4679-eff2-46df-a06b-b09a2ecdf237 | SRR3360711 | Gr10-1647 | 0.125 | 0.125 | 1.000 | 1.000 | 0.500 | 0.250 | 32.000 | 16.000 | 8.000 | 8.000 |
| Brighton, England | c3a01126-6977-4a92-82c9-b084534c5fab | SRR3357264 | GC8280 | 0.004 | 0.008 | 0.030 | 0.125 | 0.125 | 0.125 | 0.015 | 0.016 | 4.000 | 2.000 |
| Brighton, England | c409e469-40f9-4f1e-9ab1-a7fea6332de0 | SRR3360712 | Gr09-1198 | 0.125 | 0.125 | 1.000 | 1.000 | 0.250 | 0.250 | 16.000 | 16.000 | 8.000 | 8.000 |
| Brighton, England | c5541b4f-c41f-4c8b-8261-fc3799eba045 | SRR3360713 | Gr10-1620 | 0.008 | 0.008 | 8.000 | 4.000 | 0.060 | 0.125 | 8.000 | 16.000 | 32.000 | 64.000 |
| Brighton, England | c57995c0-d5ae-42c3-9866-61b76c592840 | SRR3360714 | Gr10-1121 | 0.125 | 0.125 | 0.500 | 1.000 | 0.500 | 0.250 | 16.000 | 16.000 | 8.000 | 8.000 |
| Brighton, England | ca15592f-c2ca-40fd-833c-a1c502220839 | SRR3360867 | Gr04-0011 | 0.015 | 0.008 | 8.000 | 4.000 | 0.060 | 0.063 | 8.000 | 16.000 | 64.000 | 32.000 |
| Brighton, England | ca400b40-aede-4a29-b934-d2ea0b503801 | SRR3349673 | GC11219 | 0.060 | 0.031 | 0.250 | 0.500 | 0.125 | 0.250 | 16.000 | 8.000 | 4.000 | 8.000 |
| Brighton, England | cb844c4f-9c19-4e3e-9174-8bacdd23a901 | SRR3360715 | Gr09-0596 | 0.004 | 0.004 | 0.030 | 0.031 | 0.125 | 0.125 | 0.015 | 0.016 | 0.250 | 0.500 |
| Brighton, England | cbb0806f-4867-44d5-a320-80ed51695b47 | SRR3360716 | Gr08-2615 | 0.015 | 0.016 | 1.000 | 0.500 | 0.125 | 0.250 | 16.000 | 16.000 | 2.000 | 4.000 |
| Brighton, England | cefef4c9-5bcd-42b1-8ec9-9d126305dcd8 | SRR3360717 | Gr07-1631 | 0.008 | 0.008 | 0.250 | 0.125 | 8.000 | 8.000 | 0.015 | 0.016 | 2.000 | 2.000 |
| Brighton, England | d01b9c3d-942d-49fc-aa6a-849345af4f12 | SRR3360718 | Gr10-1041 | 0.250 | 0.125 | 1.000 | 1.000 | 0.500 | 0.250 | 32.000 | 16.000 | 8.000 | 8.000 |
| Brighton, England | d1134c00-4628-463e-918a-c04fc09fcce3 | SRR3360872 | Gr04-0019 | 0.004 | 0.008 | 0.125 | 0.125 | 0.060 | 0.125 | 0.015 | 0.016 | 64.000 | 64.000 |
| Brighton, England | d24f3a1a-f44b-40dc-b4bd-f469f82d40e9 | SRR3360719 | Gr09-1246 | 0.125 | 0.125 | 1.000 | 1.000 | 0.500 | 0.250 | 32.000 | 16.000 | 8.000 | 8.000 |
| Brighton, England | d29e9c1e-16ea-4b78-9414-e8a2601b2973 | SRR3360720 | Gr07-0315 | 0.015 | 0.016 | 1.000 | 1.000 | 0.500 | 0.250 | 32.000 | 16.000 | 8.000 | 8.000 |
| Brighton, England | d3814bca-8f1e-4287-bcce-93797f543a3f | SRR3360721 | Gr09-0030 | 0.030 | 0.016 | 0.500 | 0.500 | 0.125 | 0.250 | 0.015 | 0.016 | 4.000 | 8.000 |
| Brighton, England | d5a722f0-4d69-4fd4-b82a-1f0508976e3c | SRR3349688 | GC11297 | 0.060 | 0.125 | 0.500 | 1.000 | 0.500 | 0.250 | 16.000 | 16.000 | 8.000 | 8.000 |
| Brighton, England | d779b69c-8760-4d84-b9a4-3a09cfc76649 | SRR3357288 | GC8304 | 0.125 | 0.125 | 0.500 | 0.500 | 0.500 | 0.250 | 16.000 | 16.000 | 4.000 | 2.000 |
| Brighton, England | d7c663f3-70c0-4473-93d0-67a5fa947496 | SRR3360722 | Gr09-0647 | 0.002 | 0.004 | 0.030 | 0.031 | 0.125 | 0.125 | 0.015 | 0.016 | 0.250 | 0.500 |
| Brighton, England | d7c6b2dd-a24d-49f9-a7e7-09e6d4e5ea61 | SRR3357289 | GC8231 | 0.008 | 0.008 | 0.125 | 0.250 | 0.125 | 0.250 | 2.000 | 2.000 | 16.000 | 32.000 |
| Brighton, England | d7d71fd5-6450-430b-b982-29bac412d151 | SRR3360876 | Gr04-0058 | 0.008 | 0.008 | 0.125 | 0.125 | 0.125 | 0.125 | 0.015 | 0.016 | 64.000 | 64.000 |
| Brighton, England | da88ef08-f121-46ec-bd0a-c7a9bd8b4ad0 | SRR3360723 | Gr10-1122 | 0.125 | 0.125 | 1.000 | 1.000 | 0.250 | 0.250 | 16.000 | 16.000 | 8.000 | 8.000 |
| Brighton, England | db290d47-0348-4e2a-b31a-15352c5a219b | SRR3357305 | GC8265 | 0.060 | 0.016 | 0.500 | 0.250 | 0.250 | 0.125 | 0.015 | 0.016 | 2.000 | 2.000 |
| Brighton, England | dd19ab42-3ec6-469f-b43a-65367aeac563 | SRR3360883 | Gr04-0041 | 0.008 | 0.008 | 0.060 | 0.250 | 0.030 | 0.125 | 0.125 | 0.125 | 2.000 | 2.000 |
| Brighton, England | ddc1ef8f-a819-4511-b986-6f35c43335a5 | SRR3360725 | Gr10-2229 | 0.008 | 0.008 | 0.125 | 0.125 | 0.125 | 0.125 | 0.030 | 0.016 | 2.000 | 2.000 |
| Brighton, England | e01006f1-126d-4a4a-be9c-1ce87fec1e41 | SRR3360726 | Gr09-0971 | 0.015 | 0.008 | 0.250 | 0.125 | 0.125 | 0.125 | 0.015 | 0.016 | 4.000 | 2.000 |
| Brighton, England | e0de7301-9615-4f28-8bbb-3f192e978b6f | SRR3360727 | Gr10-2214 | 0.008 | 0.008 | 0.125 | 0.125 | 0.250 | 0.125 | 0.030 | 0.016 | 4.000 | 64.000 |
| Brighton, England | e210b142-0823-4ce5-8ec0-66e4e4bcf44a | SRR3360728 | Gr10-2235 | 0.125 | 0.125 | 1.000 | 1.000 | 0.250 | 0.250 | 16.000 | 16.000 | 8.000 | 8.000 |
| Brighton, England | e2513f42-2b7c-43e1-8c60-55f8fc07123d | SRR3360729 | Gr09-1274 | 0.015 | 0.004 | 0.125 | 0.063 | 0.060 | 0.125 | 0.015 | 0.016 | 0.500 | 0.500 |
| Brighton, England | e337e896-21b3-4d70-a419-78d1f13b122c | SRR3360730 | Gr07-1105 | 0.004 | 0.008 | 0.500 | 0.125 | 0.250 | 0.125 | 0.015 | 0.016 | 4.000 | 2.000 |
| Brighton, England | e3b8f922-f997-471b-8dfe-8e0dd2ee4629 | SRR3360731 | Gr10-1682 | 0.125 | 0.125 | 1.000 | 1.000 | 0.250 | 0.250 | 16.000 | 16.000 | 8.000 | 8.000 |
| Brighton, England | e59ec5ec-db53-464e-ad7c-9c768d400ae1 | SRR3360889 | Gr04-0052 | 0.030 | 0.016 | 1.000 | 1.000 | 0.125 | 0.250 | 8.000 | 16.000 | 4.000 | 8.000 |
| Brighton, England | e68514bd-6632-4d10-bd0a-ab2c8bdd1c75 | SRR3360733 | Gr09-0649 | 0.015 | 0.008 | 0.125 | 0.250 | 0.250 | 0.250 | 32.000 | 16.000 | 8.000 | 8.000 |
| Brighton, England | e75c2f80-4c4c-467a-9466-ca3d013ee663 | SRR3360734 | Gr10-2230 | 0.008 | 0.008 | 0.250 | 0.125 | 0.125 | 0.125 | 0.030 | 0.016 | 2.000 | 2.000 |
| Brighton, England | e7a7cd3c-b147-4b04-860b-78d1cbec4c2e | SRR3360735 | Gr10-1072 | 0.002 | 0.004 | 0.060 | 0.063 | 0.060 | 0.125 | 0.030 | 0.016 | 0.250 | 0.500 |
| Brighton, England | e82c46b3-2c6f-4283-b5b3-0587a20f18fb | SRR3360736 | Gr09-0042 | 0.030 | 0.008 | 0.500 | 0.250 | 0.125 | 0.063 | 8.000 | 16.000 | 64.000 | 64.000 |
| Brighton, England | e887e6f4-425e-44ce-b4a7-dd177a9b1409 | SRR3360890 | Gr05-0373 | 0.002 | 0.004 | 0.030 | 0.063 | 0.125 | 0.125 | 0.015 | 0.016 | 0.500 | 0.250 |
| Brighton, England | e93c6fd4-81c7-4a39-a53a-a51558ad710e | SRR3360737 | Gr08-1774 | 0.002 | 0.004 | 0.060 | 0.063 | 0.060 | 0.125 | 0.015 | 0.016 | 0.500 | 0.500 |
| Brighton, England | ea327f06-ea45-482d-9a8e-d0328803201f | SRR3360738 | Gr09-0043 | 0.015 | 0.016 | 1.000 | 1.000 | 0.125 | 0.250 | 32.000 | 16.000 | 4.000 | 8.000 |
| Brighton, England | eb0e3606-fcd6-4354-b021-715d854fadba | SRR3357314 | GC8241 | 0.060 | 0.016 | 1.000 | 1.000 | 0.250 | 0.250 | 32.000 | 16.000 | 8.000 | 8.000 |
| Brighton, England | ec7ef850-d300-43f9-9146-46ef4e19914b | SRR3360740 | Gr10-1558 |  |  | 0.250 | 0.250 | 0.250 | 0.125 | 0.030 | 0.016 | 0.500 | 0.500 |
| Brighton, England | ed83ff21-c7fd-4b4c-93f0-5af2819657ee | SRR3360893 | Gr05-0379 | 0.015 | 0.016 | 0.500 | 0.500 | 0.250 | 0.250 | 16.000 | 8.000 | 8.000 | 8.000 |
| Brighton, England | f396aef5-8086-40e8-9656-e0a0f71557ea | SRR3360743 | Gr09-0593 | 0.125 | 0.125 | 0.500 | 1.000 | 0.250 | 0.250 | 32.000 | 16.000 | 8.000 | 8.000 |
| Brighton, England | f76e77cd-9f3f-4040-b5c9-3c1394e399cb | SRR3360745 | Gr09-0650 | 0.015 | 0.008 | 0.125 | 0.125 | 0.250 | 0.125 | 0.015 | 0.016 | 4.000 | 2.000 |
| Brighton, England | f7e09716-182d-418c-a0ae-efbd19317730 | SRR3349726 | GC11231 | 0.015 | 0.008 | 1.000 | 2.000 | 0.125 | 0.063 | 0.015 | 0.016 | 4.000 | 2.000 |
| Brighton, England | f834b15a-b0c0-400d-af49-49e36aa342dd | SRR3360746 | Gr10-2228 | 0.008 | 0.008 | 0.250 | 0.125 | 0.250 | 0.125 | 0.030 | 0.016 | 4.000 | 2.000 |
| Brighton, England | f893ffe6-459c-421e-b1de-4f50b62a7f78 | SRR3360747 | Gr08-2614 | 0.004 | 0.008 | 0.125 | 0.125 | 0.125 | 0.125 | 0.015 | 0.016 | 4.000 | 2.000 |
| Brighton, England | f9e44032-d249-4e6e-b1b2-fd2e686032cc | SRR3360748 | Gr09-0100 | 0.125 | 0.125 | 0.500 | 1.000 | 0.500 | 0.250 | 32.000 | 16.000 | 4.000 | 8.000 |
| Brighton, England | f9efd35e-a673-46bc-a730-2b69622f3c53 | SRR3360749 | Gr08-1151 | 0.015 | 0.008 | 0.500 | 0.250 | 0.250 | 0.125 | 0.015 | 0.016 | 4.000 | 32.000 |
| Brighton, England | fa2333da-c348-4a4e-949f-25717d2d603e | SRR3360750 | Gr07-1636 | 0.030 | 0.008 | 8.000 | 4.000 | 0.125 | 0.125 | 4.000 | 2.000 | 64.000 | 64.000 |
| Brighton, England | fadcfe0b-b49f-423f-b990-4c01e6d8bd6b | SRR3360751 | Gr05-1689 | 0.015 | 0.016 | 1.000 | 0.500 | 0.500 | 0.250 | 16.000 | 16.000 | 4.000 | 2.000 |
| Brighton, England | fc3bb6ef-a3c7-4336-ac71-fb600b0a2f23 | SRR3360752 | Gr09-0572 | 0.008 | 0.008 | 0.125 | 0.250 | 0.500 | 0.250 | 32.000 | 16.000 | 8.000 | 8.000 |
| Brighton, England | fd24d58f-5e8d-4caf-a028-9d241e91058c | SRR3360753 | Gr10-1119 | 0.125 | 0.125 | 1.000 | 1.000 | 0.500 | 0.250 | 16.000 | 16.000 | 8.000 | 8.000 |
| Brighton, England | fd29694e-4e38-459f-a234-258f34febefc | SRR3360754 | Gr07-0427 | 0.004 | 0.008 | 0.125 | 0.125 | 0.250 | 0.125 | 0.015 | 0.016 | 4.000 | 2.000 |
| Brighton, England | fe49374c-6b5a-4b37-964c-3391131651b4 | SRR3360755 | Gr09-1212 | 0.008 | 0.008 | 0.250 | 0.250 | 0.500 | 0.250 | 32.000 | 16.000 | 8.000 | 8.000 |
| Canada | 005d594b-116d-4c54-a995-d7a8ae2b8864 | SRR2736194 | SRR2736194 | 0.004 | 0.008 | 0.250 | 0.250 | 8.000 | 8.000 | 0.015 | 0.016 | 1.000 | 2.000 |
| Canada | 018f366e-4a06-40b1-b32f-82d7ac0eab9f | SRR2736169 | SRR2736169 | 0.008 | 0.008 | 0.500 | 0.250 | 4.000 | 4.000 | 0.015 | 0.016 | 1.000 | 1.000 |
| Canada | 01b6bd71-e1ac-4e3f-819b-e928c156532d | SRR2736249 | SRR2736249 | 0.016 | 0.016 | 1.000 | 1.000 | 2.000 | 2.000 | 0.032 | 0.016 | 4.000 | 4.000 |
| Canada | 01d916b5-c60b-4542-bad0-b224d9c7de4e | SRR2736279 | SRR2736279 | 0.125 | 0.125 | 4.000 | 2.000 | 16.000 | 8.000 | 16.000 | 16.000 | 8.000 | 4.000 |
| Canada | 02169bc2-777a-4c1c-909d-c9416aeeed0c | SRR2736198 | SRR2736198 | 0.125 | 0.016 | 2.000 | 2.000 | 2.000 | 8.000 | 16.000 | 16.000 | 4.000 | 4.000 |
| Canada | 0244ed5f-7c7a-4870-b00f-eba51c9b5e66 | SRR1661330 | WHO-O | 0.016 | 0.031 | 64.000 | 128.000 | 0.250 | 1.000 | 0.015 | 0.016 | 1.000 | 4.000 |
| Canada | 0551fe6f-749b-498e-be12-dee769e75b67 | SRR2736235 | SRR2736235 | 0.008 | 0.008 | 0.250 | 0.250 | 8.000 | 8.000 | 0.015 | 0.016 | 2.000 | 2.000 |
| Canada | 05c7540a-48df-4b18-8a15-4eb6ef413bb0 | SRR2736115 | SRR2736115 | 0.008 | 0.016 | 1.000 | 2.000 | 2.000 | 1.000 | 0.016 | 0.016 | 4.000 | 4.000 |
| Canada | 077589e1-4d63-4a65-9ce0-3d9864d25f75 | SRR2736114 | SRR2736114 | 0.008 | 0.016 | 1.000 | 2.000 | 2.000 | 1.000 | 16.000 | 16.000 | 4.000 | 4.000 |
| Canada | 077fd96f-cd35-4d3f-97cb-b7963f8e46a9 | SRR2736146 | SRR2736146 | 0.016 | 0.016 | 1.000 | 1.000 | 2.000 | 1.000 | 8.000 | 16.000 | 1.000 | 2.000 |
| Canada | 08b86a5e-1b3b-468e-b8c1-a2f12019b9ee | SRR2736251 | SRR2736251 | 0.008 | 0.008 | 0.250 | 0.250 | 16.000 | 8.000 | 0.015 | 0.016 | 1.000 | 2.000 |
| Canada | 09930387-b1a9-4f2d-862a-8443286ad52d | SRR1661175 | SRR1661175 | 0.125 | 0.125 | 2.000 | 2.000 | 0.500 | 1.000 | 16.000 | 16.000 | 4.000 | 4.000 |
| Canada | 0aa538e6-4699-4d48-a633-af85e8e9f149 | SRR2736262 | SRR2736262 | 0.032 | 0.016 | 8.000 | 2.000 | 2.000 | 1.000 | 0.032 | 0.016 | 4.000 | 4.000 |
| Canada | 0ab97f74-48b4-4e43-9825-3af46c8b0b59 | SRR2736218 | SRR2736218 | 0.032 | 0.008 | 0.500 | 0.250 | 0.250 | 0.500 | 0.015 | 0.016 | 2.000 | 1.000 |
| Canada | 0cae1e1b-a15e-40fb-88a5-008159defe07 | SRR2736179 | SRR2736179 | 0.008 | 0.008 | 0.250 | 0.250 | 4.000 | 4.000 | 0.015 | 0.016 | 2.000 | 1.000 |
| Canada | 0ea7fbd1-f223-4635-90b3-35843a6812ad | SRR2736134 | SRR2736134 | |  |  |  | 512.000 | 512.000 | 4.000 | 2.000 | 64.000 | 32.000 |
| Canada | 10f429bd-55a3-4077-9246-683ce35aa1e0 | SRR2736177 | SRR2736177 | 0.032 | 0.031 | 2.000 | 2.000 | 2.000 | 4.000 | 16.000 | 16.000 | 4.000 | 4.000 |
| Canada | 1179438c-3f5f-4b08-8e17-bbdb2b090704 | SRR2736168 | SRR2736168 | 0.032 | 0.031 | 2.000 | 2.000 | 16.000 | 8.000 | 16.000 | 16.000 | 4.000 | 4.000 |
| Canada | 11f143dd-fd78-4263-a45d-cfabaf26cc00 | SRR2736233 | SRR2736233 | 0.008 | 0.008 | 0.125 | 0.250 | 8.000 | 8.000 | 0.015 | 0.016 | 2.000 | 2.000 |
| Canada | 12299188-e591-4522-967f-c6107f3a7bb6 | SRR2736152 | SRR2736152 | 0.032 | 0.016 | 0.500 | 0.500 | 8.000 | 8.000 | 0.015 | 0.016 | 1.000 | 1.000 |
| Canada | 1302dd28-61e8-4075-9da6-73151c98ed88 | SRR2736096 | SRR2736096 | 0.016 | 0.016 | 4.000 | 2.000 | 2.000 | 1.000 | 32.000 | 16.000 | 8.000 | 4.000 |
| Canada | 138bd2b1-2abc-4d34-b162-16a7a011fb7d | SRR2736139 | SRR2736139 | 0.016 | 0.016 | 2.000 | 2.000 | 16.000 | 8.000 | 16.000 | 16.000 | 4.000 | 4.000 |
| Canada | 14dc2136-9d8a-49d9-94aa-e3a14c107f82 | SRR2736175 | SRR2736175 | 0.032 | 0.031 | 2.000 | 2.000 | 2.000 | 1.000 | 16.000 | 16.000 | 8.000 | 4.000 |
| Canada | 14e1672f-3c25-40ce-8a13-da072b2cc5f0 | SRR2736192 | SRR2736192 | 0.008 | 0.008 | 0.500 | 0.250 | 8.000 | 8.000 | 0.015 | 0.016 | 1.000 | 2.000 |
| Canada | 14e611c2-6401-49ee-b5c3-9e2d7d99c8fc | SRR2736220 | SRR2736220 | 0.125 | 0.125 | 8.000 | 2.000 | 16.000 | 8.000 | 16.000 | 16.000 | 4.000 | 4.000 |
| Canada | 15d5d7c6-e645-4d64-9c71-5965752d8bf8 | SRR2736099 | SRR2736099 | 0.032 | 0.016 | 4.000 | 2.000 | 2.000 | 1.000 | 0.016 | 0.016 | 8.000 | 4.000 |
| Canada | 179fb783-ec70-479c-9357-02cf8429cbd4 | SRR1661324 | WHO-F | 0.008 | 0.004 | 0.032 | 0.125 | 0.125 | 0.500 | 0.015 | 0.016 | 0.250 | 0.250 |
| Canada | 18a5c1d2-1baa-4ea4-959b-c22bc885701b | SRR2736267 | SRR2736267 | 0.004 | 0.016 | 0.250 | 0.500 | 8.000 | 8.000 | 0.015 | 0.016 | 2.000 | 1.000 |
| Canada | 193f067b-4f77-4355-b718-32c15930ff7d | SRR2736242 | SRR2736242 | 0.032 | 0.016 | 0.500 | 0.500 | 16.000 | 8.000 | 0.016 | 0.016 | 2.000 | 2.000 |
| Canada | 1aea9e32-08c8-4b0f-964d-9d14d03617df | SRR2736287 | SRR2736287 | 0.004 | 0.008 | 0.125 | 0.250 | 8.000 | 8.000 | 0.015 | 0.016 | 1.000 | 2.000 |
| Canada | 1af44cd5-c8fd-4fcd-898d-3edca783ff47 | SRR2736174 | SRR2736174 | 0.016 | 0.008 | 0.500 | 0.250 | 4.000 | 4.000 | 0.015 | 0.016 | 1.000 | 1.000 |
| Canada | 1b7a2136-82bd-47ac-9592-01b7c9e38722 | SRR2736294 | SRR2736294 | 0.016 | 0.008 | 0.500 | 0.500 | 2.000 | 2.000 | 0.015 | 0.016 | 2.000 | 2.000 |
| Canada | 1b892625-35e4-478c-a9fb-f2da78d7ef5d | SRR2736102 | SRR2736102 | 0.032 | 0.016 | 4.000 | 2.000 | 2.000 | 1.000 | 0.016 | 0.016 | 8.000 | 4.000 |
| Canada | 1cb056fa-cd94-44b1-8d1b-793af9a90940 | SRR2736135 | SRR2736135 | 0.008 | 0.031 | 0.500 | 2.000 | 2.000 | 1.000 | 32.000 | 16.000 | 2.000 | 4.000 |
| Canada | 1e802382-83d9-4a92-a89a-26e28c595d10 | SRR1661262 | SRR1661262 | 0.008 | 0.016 | 2.000 | 0.500 | 1.000 | 1.000 | 16.000 | 16.000 | 4.000 | 4.000 |
| Canada | 1f7bd07f-80ce-436b-b84b-07975f565fbf | SRR2736289 | SRR2736289 | 0.004 | 0.008 | 0.125 | 0.250 | 8.000 | 8.000 | 0.015 | 0.016 | 1.000 | 1.000 |
| Canada | 221cf694-49b5-43f6-afbb-20cc2ff5b5c7 | SRR2736137 | SRR2736137 | 0.016 | 0.016 | 0.250 | 0.500 | 2.000 | 8.000 | 0.015 | 0.016 | 1.000 | 1.000 |
| Canada | 2281faf4-20dc-4ff6-9944-23a498212fec | SRR2736214 | SRR2736214 | 0.016 | 0.031 | 4.000 | 2.000 | 512.000 | 512.000 | 32.000 | 16.000 | 2.000 | 4.000 |
| Canada | 25850c14-be76-4a13-a2a7-a3698bebecef | SRR2736302 | SRR2736302 | 0.008 | 0.008 | 0.250 | 0.250 | 4.000 | 8.000 | 0.015 | 0.016 | 1.000 | 2.000 |
| Canada | 258db2ba-42ac-4944-99de-56797924de19 | SRR2736252 | SRR2736252 | 0.008 | 0.008 | 0.250 | 0.250 | 8.000 | 8.000 | 0.015 | 0.016 | 1.000 | 2.000 |
| Canada | 25ece4d2-3e5d-45e8-aa13-521a7808c3ab | SRR2736217 | SRR2736217 | 0.016 | 0.008 | 0.500 | 0.250 | 16.000 | 8.000 | 0.015 | 0.016 | 2.000 | 2.000 |
| Canada | 261b27ad-b655-46ea-8fca-023075979c08 | SRR2736282 | SRR2736282 | 0.008 | 0.008 | 0.250 | 0.250 | 8.000 | 8.000 | 0.015 | 0.016 | 1.000 | 2.000 |
| Canada | 29050ba5-2d1c-4a55-ae86-7a9be6d3a629 | SRR1661323 | ATCC49226 | 0.016 | 0.031 | 0.500 | 1.000 | 0.500 | 1.000 | 0.015 | 0.016 | 0.500 | 2.000 |
| Canada | 293e7ed7-7032-4ff8-8c09-e69cf37aeb77 | SRR1661168 | SRR1661168 | 0.500 | 0.250 | 4.000 | 4.000 | 0.500 | 1.000 |  |  | 4.000 | 4.000 |
| Canada | 2a471979-b2c9-4819-bf79-52b8ecd2868e | SRR2736124 | SRR2736124 | 0.032 | 0.016 | 2.000 | 2.000 | 2.000 | 1.000 | 0.016 | 0.016 | 8.000 | 4.000 |
| Canada | 2ab5b9bb-a368-4199-a243-40cda5500f3b | SRR2736178 | SRR2736178 | 0.125 | 0.125 | 2.000 | 2.000 | 2.000 | 1.000 | 16.000 | 16.000 | 8.000 | 4.000 |
| Canada | 2d52f606-a74b-4099-8ad4-fadc58bdb8b9 | SRR2736112 | SRR2736112 | 0.008 | 0.016 | 4.000 | 2.000 | 2.000 | 1.000 | 0.016 | 0.016 | 4.000 | 4.000 |
| Canada | 2da3b801-7083-4bee-a595-4781a9e79524 | SRR2736286 | SRR2736286 | 0.008 | 0.008 | 0.125 | 0.250 | 8.000 | 8.000 | 0.015 | 0.016 | 1.000 | 2.000 |
| Canada | 2ecb079e-a14b-438f-bb09-d1d6e7e8e4f1 | SRR2736232 | SRR2736232 | 0.125 | 0.125 | 2.000 | 2.000 | 8.000 | 8.000 | 16.000 | 16.000 | 8.000 | 4.000 |
| Canada | 2efc5c7c-578f-47db-b180-7cefeef9ebb5 | SRR2736301 | SRR2736301 | 0.008 | 0.008 | 0.500 | 0.250 | 1.000 | 1.000 | 0.015 | 0.016 | 2.000 | 2.000 |
| Canada | 2f3ede49-6d3d-4b1c-b4d8-bd9c1d90e8e1 | SRR2736195 | SRR2736195 | 0.004 | 0.008 | 0.125 | 0.250 | 8.000 | 8.000 | 0.015 | 0.016 | 1.000 | 2.000 |
| Canada | 304c9fe0-8d60-4a2e-a5c3-32b2110cc5f3 | SRR2736284 | SRR2736284 | 0.016 | 0.008 | 0.125 | 0.250 | 8.000 | 8.000 | 0.015 | 0.016 | 2.000 | 2.000 |
| Canada | 3136f64b-4dbb-431c-8ae5-3a34f9fdc91a | SRR2736172 | SRR2736172 | 0.008 | 0.008 | 0.250 | 0.250 | 2.000 | 4.000 | 0.015 | 0.016 | 1.000 | 1.000 |
| Canada | 32a611a2-f7fd-4b97-9488-5b248534456e | SRR2736116 | SRR2736116 | 0.016 | 0.016 | 1.000 | 2.000 | 2.000 | 1.000 | 0.016 | 0.016 | 4.000 | 4.000 |
| Canada | 32f77ba7-4ca7-4553-ad26-74b701acb900 | SRR1661326 | WHO-K | 0.500 | 0.250 | 2.000 | 4.000 | 0.250 | 1.000 | 32.000 | 16.000 | 2.000 | 4.000 |
| Canada | 3376bef8-3ec6-49f2-a4dd-3468d6b6f1de | SRR2736154 | SRR2736154 | 0.063 | 0.031 | 4.000 | 2.000 | 16.000 | 8.000 | 16.000 | 16.000 | 2.000 | 4.000 |
| Canada | 35a880ed-3a35-41d0-ae53-7429271539c4 | SRR2736274 | SRR2736274 | 0.032 | 0.063 | 0.500 | 1.000 | 8.000 | 8.000 | 8.000 | 8.000 | 4.000 | 4.000 |
| Canada | 37a27f1b-1bd8-4b70-9a49-07d0a96546bb | SRR2736104 | SRR2736104 | 0.032 | 0.016 | 4.000 | 2.000 | 2.000 | 1.000 | 0.032 | 0.016 | 8.000 | 4.000 |
| Canada | 37f70285-0fd6-40c1-a85c-0a50a353adbe | SRR2736306 | SRR2736306 | 0.016 | 0.008 | 0.250 | 0.250 | 8.000 | 8.000 | 0.015 | 0.016 | 2.000 | 2.000 |
| Canada | 398d4686-1187-45c4-b7c6-a4be16cb7764 | SRR2736119 | SRR2736119 | 0.016 | 0.016 | 4.000 | 2.000 | 2.000 | 1.000 | 0.032 | 0.016 | 4.000 | 4.000 |
| Canada | 3ab04b22-adab-4fc8-8753-f6a9ce1738f5 | SRR2736213 | SRR2736213 | 0.032 | 0.031 | 2.000 | 2.000 | 2.000 | 1.000 | 16.000 | 16.000 | 4.000 | 4.000 |
| Canada | 3c4403a3-579b-401e-9adb-2385f3a57377 | SRR2736147 | SRR2736147 | 0.016 | 0.031 | 2.000 | 2.000 | 2.000 | 4.000 | 16.000 | 16.000 | 2.000 | 4.000 |
| Canada | 3d2eb64b-abe2-4c8d-b41e-01134022303d | SRR2736159 | SRR2736159 | 0.008 | 0.008 | 0.500 | 0.500 | 2.000 | 2.000 | 0.015 | 0.016 | 2.000 | 2.000 |
| Canada | 3e15738a-249d-4fb0-a9fa-644b49d77cd2 | SRR2736095 | SRR2736095 | 0.032 | 0.016 | 1.000 | 2.000 | 2.000 | 1.000 | 0.015 | 0.016 | 4.000 | 4.000 |
| Canada | 3eaa7dc5-7d40-417f-acb4-4a9ded00b180 | SRR2736291 | SRR2736291 | 0.016 | 0.008 | 0.125 | 0.250 | 8.000 | 8.000 | 0.015 | 0.016 | 2.000 | 2.000 |
| Canada | 3edb8301-d391-4951-9467-1009efe5b9fc | SRR2736211 | SRR2736211 | 0.016 | 0.008 | 0.250 | 0.250 | 1.000 | 1.000 | 0.015 | 0.016 | 1.000 | 2.000 |
| Canada | 3f9efa9a-474b-4147-93c2-1937a42a6ee7 | SRR2736202 | SRR2736202 | |  |  |  | 16.000 | 8.000 | 0.015 | 0.016 | 1.000 | 2.000 |
| Canada | 3fb47e4e-6ced-46d2-ac60-d250027a4f7c | SRR2736277 | SRR2736277 | 0.125 | 0.125 | 2.000 | 2.000 | 8.000 | 8.000 | 16.000 | 16.000 | 4.000 | 4.000 |
| Canada | 40377274-6e2f-46f6-9fef-73f0b067ce2e | SRR2736094 | SRR2736094 | 0.008 | 0.016 | 1.000 | 2.000 | 2.000 | 1.000 | 0.016 | 0.016 | 4.000 | 4.000 |
| Canada | 40f6a455-4a8f-4bb3-bfc4-994dd7f7b0f8 | SRR2736184 | SRR2736184 | 0.008 | 0.008 | 0.250 | 0.250 | 4.000 | 4.000 | 0.015 | 0.016 | 1.000 | 1.000 |
| Canada | 449e6759-764b-444d-9740-56801e91fc90 | SRR2736131 | SRR2736131 | 0.008 | 0.016 | 0.250 | 0.500 | 4.000 | 8.000 | 0.015 | 0.016 | 1.000 | 1.000 |
| Canada | 4533a533-329f-4c31-aa9a-660ecd3a3a6a | SRR2736160 | SRR2736160 | |  |  |  | 16.000 | 8.000 | 16.000 | 16.000 | 4.000 | 4.000 |
| Canada | 46824b9c-aa10-4f18-807f-eb7ba00d5e5a | SRR2736272 | SRR2736272 | 0.008 | 0.008 | 0.500 | 0.500 | 4.000 | 2.000 | 0.015 | 0.016 | 4.000 | 2.000 |
| Canada | 4ab29d30-e2d2-4fd0-a5bd-4c6a22149db7 | SRR2736230 | SRR2736230 | 0.125 | 0.125 | 2.000 | 2.000 | 8.000 | 8.000 | 16.000 | 16.000 | 8.000 | 4.000 |
| Canada | 4c8b487a-1556-45d5-92f5-e5d5a74ee2b0 | SRR2736285 | SRR2736285 | 0.008 | 0.008 | 0.125 | 0.250 | 8.000 | 8.000 | 0.015 | 0.016 | 2.000 | 2.000 |
| Canada | 4cbe433e-ed66-4777-97e6-12122964cbc0 | SRR2736127 | SRR2736127 | 0.016 | 0.016 | 1.000 | 0.500 | 2.000 | 2.000 | 0.125 | 0.125 | 2.000 | 1.000 |
| Canada | 4ccbb326-3e8e-45f4-829b-6ba68039bae2 | SRR2736153 | SRR2736153 | 0.032 | 0.016 | 0.500 | 0.500 | 8.000 | 8.000 | 0.015 | 0.016 | 1.000 | 1.000 |
| Canada | 4dbb104b-a7bb-43b9-a635-d74e50996d85 | SRR2736200 | SRR2736200 | 0.008 | 0.008 | 0.500 | 0.250 | 4.000 | 4.000 | 0.015 | 0.016 | 1.000 | 1.000 |
| Canada | 4dbe6a46-8008-4d5f-a11c-2e0188863486 | SRR2736148 | SRR2736148 | 0.032 | 0.031 | 2.000 | 2.000 | 2.000 | 4.000 | 16.000 | 16.000 | 8.000 | 64.000 |
| Canada | 4f9c39e1-9300-415e-81d0-f9bd0b468ee5 | SRR2736110 | SRR2736110 | 0.016 | 0.016 | 1.000 | 0.500 | 4.000 | 4.000 | 0.015 | 0.016 | 2.000 | 1.000 |
| Canada | 4fc98ea8-c4c3-46d3-ae9b-82b25f3f47ef | SRR2736165 | SRR2736165 | 0.008 | 0.016 | 1.000 | 2.000 | 2.000 | 1.000 | 32.000 | 16.000 | 2.000 | 4.000 |
| Canada | 506aba83-879b-4aa1-a679-db0ccf2cda1c | SRR2736098 | SRR2736098 | 0.016 | 0.016 | 2.000 | 2.000 | 2.000 | 1.000 | 0.016 | 0.016 | 4.000 | 4.000 |
| Canada | 50d53221-dc02-4882-86fa-ee4d45c05467 | SRR2736237 | SRR2736237 | 0.008 | 0.008 | 0.250 | 0.250 | 8.000 | 8.000 | 0.015 | 0.016 | 2.000 | 2.000 |
| Canada | 528bcdc9-8bca-4973-b4da-768df82c8952 | SRR2736278 | SRR2736278 | 0.008 | 0.008 | 0.125 | 0.250 | 8.000 | 8.000 | 0.015 | 0.016 | 1.000 | 2.000 |
| Canada | 528e2a67-0101-4091-8e50-5cb381a4d4d2 | SRR2736276 | SRR2736276 | 0.125 | 0.125 | 2.000 | 2.000 | 8.000 | 4.000 | 16.000 | 16.000 | 4.000 | 4.000 |
| Canada | 53ba411e-1542-46b9-b404-22ae37c785e9 | SRR2736280 | SRR2736280 | 0.125 | 0.125 | 2.000 | 2.000 | 2.000 | 1.000 | 16.000 | 16.000 | 8.000 | 4.000 |
| Canada | 53d51764-9037-49a6-9bcb-81b54e95fb5c | SRR2736268 | SRR2736268 | 0.016 | 0.008 | 0.125 | 0.250 | 8.000 | 8.000 | 0.015 | 0.016 | 2.000 | 2.000 |
| Canada | 542f1fb2-3517-4f47-ac4c-44d91019481a | SRR2736247 | SRR2736247 | 0.250 | 0.125 | 1.000 | 2.000 | 8.000 | 8.000 | 16.000 | 16.000 | 4.000 | 4.000 |
| Canada | 54b3c581-06c6-488a-af97-81d0e3343513 | SRR2736197 | SRR2736197 | 0.004 | 0.008 | 0.125 | 0.250 | 8.000 | 8.000 | 0.015 | 0.016 | 1.000 | 2.000 |
| Canada | 5589afe2-c4e4-4a7b-bbfe-512fc0315a89 | SRR2736201 | SRR2736201 | 0.016 | 0.008 | 0.250 | 0.250 | 4.000 | 4.000 | 0.015 | 0.016 | 0.500 | 1.000 |
| Canada | 5683c8d5-f1ad-4598-959e-e68a739d445e | SRR2736305 | SRR2736305 | 0.016 | 0.008 | 1.000 | 0.500 | 2.000 | 2.000 | 0.015 | 0.016 | 4.000 | 2.000 |
| Canada | 59007850-21bc-4d12-b3ae-282aee819bea | SRR2736203 | SRR2736203 | 0.032 | 0.031 | 256.000 | 64.000 | 512.000 | 512.000 | 16.000 | 16.000 | 64.000 | 16.000 |
| Canada | 59f583bf-34f3-4bd5-a814-64323346aea7 | SRR2736295 | SRR2736295 | 0.008 | 0.008 | 0.250 | 0.250 | 8.000 | 8.000 | 0.015 | 0.016 | 2.000 | 2.000 |
| Canada | 5a3c4706-c99c-4761-9322-8adc4f93a030 | SRR2736292 | SRR2736292 | 0.008 | 0.008 | 0.125 | 0.250 | 8.000 | 8.000 | 0.015 | 0.016 | 1.000 | 2.000 |
| Canada | 5b855cf2-04f7-4e2a-a885-1eb7cddb6094 | SRR2736181 | SRR2736181 | 0.008 | 0.008 | 0.250 | 0.250 | 4.000 | 4.000 | 0.015 | 0.016 | 2.000 | 2.000 |
| Canada | 5be2aa1e-52c9-4e34-9008-22cf27e27825 | SRR2736188 | SRR2736188 | 0.008 | 0.008 | 0.125 | 0.250 | 8.000 | 8.000 | 0.015 | 0.016 | 1.000 | 2.000 |
| Canada | 5c6a921c-fba8-443e-8e38-7e9dbf3bd587 | SRR2736164 | SRR2736164 | 0.063 | 0.031 | 4.000 | 2.000 | 2.000 | 4.000 | 16.000 | 16.000 | 4.000 | 4.000 |
| Canada | 5ef5c845-8891-4f89-b898-fc5ddefe45fa | SRR2736241 | SRR2736241 | 0.125 | 0.125 | 1.000 | 2.000 | 8.000 | 8.000 | 16.000 | 16.000 | 8.000 | 4.000 |
| Canada | 5f892ca4-3cc3-4fa7-b078-37860f86818f | SRR2736281 | SRR2736281 | 0.032 | 0.031 | 0.500 | 1.000 | 2.000 | 1.000 | 16.000 | 8.000 | 4.000 | 4.000 |
| Canada | 5f8af983-134b-4614-a139-1adcf2188e1c | SRR2736142 | SRR2736142 | 0.008 | 0.016 | 2.000 | 1.000 | 16.000 | 1.000 | 0.015 | 0.016 | 64.000 | 32.000 |
| Canada | 62b1d659-f41d-4ee5-a324-f847a1f40b23 | SRR1661265 | SRR1661265 | 0.032 | 0.016 | 1.000 | 1.000 | 0.500 | 8.000 | 4.000 | 16.000 | 2.000 | 2.000 |
| Canada | 62bd19ef-25b4-408b-953c-7f1d7e2aa9ae | SRR2736275 | SRR2736275 | 0.032 | 0.016 | 1.000 | 1.000 | 2.000 | 2.000 | 0.032 | 0.016 | 4.000 | 4.000 |
| Canada | 64732d48-683e-4309-bffa-df8de73c2d35 | SRR2736103 | SRR2736103 | 0.016 | 0.016 | 4.000 | 2.000 | 2.000 | 1.000 | 0.016 | 0.016 | 8.000 | 4.000 |
| Canada | 649956c2-263a-4377-b1c1-472767eed711 | SRR2736223 | SRR2736223 | 0.125 | 0.125 | 4.000 | 2.000 | 16.000 | 8.000 | 16.000 | 16.000 | 8.000 | 4.000 |
| Canada | 65539c8e-c95c-4513-be8c-6a5acd67bc3d | SRR2736140 | SRR2736140 | 0.008 | 0.016 | 0.500 | 0.500 | 4.000 | 8.000 | 0.015 | 0.016 | 1.000 | 1.000 |
| Canada | 66ce22b3-5b86-46d0-8e9a-66c9b6c6b2a1 | SRR2736163 | SRR2736163 | 0.125 | 0.125 | 4.000 | 2.000 | 16.000 | 8.000 | 16.000 | 16.000 | 4.000 | 4.000 |
| Canada | 67c42b00-ea0a-45f9-805a-b398f7fad1d1 | SRR2736100 | SRR2736100 | 0.016 | 0.016 | 2.000 | 2.000 | 2.000 | 1.000 | 0.016 | 0.016 | 8.000 | 4.000 |
| Canada | 6a7418cd-a173-448d-bcd3-3e89c3e4cd75 | SRR2736293 | SRR2736293 | 0.016 | 0.008 | 0.016 | 0.500 | 2.000 | 2.000 | 0.015 | 0.016 | 2.000 | 2.000 |
| Canada | 6c3fb8da-0dfe-4419-9144-ec5533ae49c0 | SRR2736191 | SRR2736191 | 0.008 | 0.008 | 0.250 | 0.250 | 8.000 | 8.000 | 0.015 | 0.016 | 1.000 | 2.000 |
| Canada | 6cd804a1-0ab9-4532-9943-f9cc5a9f44fa | SRR1661211 | SRR1661211 | 0.016 | 0.008 | 0.500 | 0.250 | 0.500 | 1.000 | 0.015 | 0.016 | 1.000 | 2.000 |
| Canada | 6d6bc8be-d50f-4a10-bee7-a02091a92ceb | SRR2736122 | SRR2736122 | 0.032 | 0.016 | 2.000 | 2.000 | 2.000 | 1.000 | 0.016 | 0.016 | 4.000 | 4.000 |
| Canada | 6e3a1312-50df-43c8-944e-67a948f7bb3b | SRR2736167 | SRR2736167 | 0.032 | 0.031 | 2.000 | 2.000 | 2.000 | 1.000 | 16.000 | 16.000 | 4.000 | 4.000 |
| Canada | 6eede377-942a-4d45-b650-463b6fb1e508 | SRR2736171 | SRR2736171 | 0.008 | 0.008 | 0.500 | 0.250 | 4.000 | 4.000 | 0.015 | 0.016 | 1.000 | 1.000 |
| Canada | 71d25721-e8d4-4c4c-b9cd-f78aed23a8bd | SRR2736156 | SRR2736156 | 0.032 | 0.031 | 1.000 | 2.000 | 4.000 | 1.000 | 16.000 | 16.000 | 4.000 | 4.000 |
| Canada | 729e9432-5702-4e65-ba7e-1be01775b70b | SRR2736207 | SRR2736207 | 0.032 | 0.008 | 0.250 | 0.250 | 16.000 | 8.000 | 0.015 | 0.016 | 1.000 | 2.000 |
| Canada | 74209adc-5ccb-4e16-8018-b695f6a31558 | SRR2736244 | SRR2736244 | 0.063 | 0.125 | 1.000 | 2.000 | 8.000 | 8.000 | 16.000 | 16.000 | 4.000 | 4.000 |
| Canada | 74364e3c-d571-4a59-a19e-72a8ede515ad | SRR2736259 | SRR2736259 | 0.125 | 0.125 | 4.000 | 2.000 | 16.000 | 8.000 | 16.000 | 16.000 | 4.000 | 4.000 |
| Canada | 7623a352-d3fb-4c38-bf1e-dc326d828b66 | SRR2736128 | SRR2736128 | 0.016 | 0.016 | 8.000 | 2.000 | 2.000 | 1.000 | 0.016 | 0.016 | 8.000 | 4.000 |
| Canada | 76814cbd-395e-4f6d-9818-79e2a032cf89 | SRR2736254 | SRR2736254 | 0.250 | 0.125 | 4.000 | 2.000 | 16.000 | 8.000 | 16.000 | 16.000 | 4.000 | 4.000 |
| Canada | 77456d4a-6556-44fc-a28a-17190c527c49 | SRR2736256 | SRR2736256 | 0.016 | 0.016 | 2.000 | 1.000 | 2.000 | 2.000 | 0.032 | 0.016 | 4.000 | 4.000 |
| Canada | 78be0faa-d92f-4f71-beaf-625ed3e5ee58 | SRR2736186 | SRR2736186 | 0.032 | 0.031 | 2.000 | 2.000 | 2.000 | 1.000 | 16.000 | 16.000 | 8.000 | 4.000 |
| Canada | 79094472-7385-4553-ba5a-0e810d2b2a6a | SRR1661245 | SRR1661245 | 0.250 | 0.125 | 2.000 | 2.000 | 1.000 | 1.000 | 16.000 | 16.000 | 4.000 | 4.000 |
| Canada | 792eca02-02aa-42b1-86c9-6da4f3e2e90b | SRR2736222 | SRR2736222 | 0.125 | 0.125 | 4.000 | 2.000 | 8.000 | 8.000 | 16.000 | 16.000 | 2.000 | 4.000 |
| Canada | 79c856cc-5735-40d2-b202-c85158e0694b | SRR2736151 | SRR2736151 | 0.016 | 0.031 | 1.000 | 2.000 | 2.000 | 4.000 | 16.000 | 16.000 | 2.000 | 4.000 |
| Canada | 7af0cb87-700e-49ec-9c1e-f0611ebbf512 | SRR2736138 | SRR2736138 | 0.016 | 0.016 | 2.000 | 2.000 | 8.000 | 8.000 |  |  | 4.000 | 4.000 |
| Canada | 7cb76bc3-55ab-4b61-a06c-78df194c9b99 | SRR2736209 | SRR2736209 | 0.032 | 0.125 | 4.000 | 2.000 | 16.000 | 1.000 | 16.000 | 16.000 | 4.000 | 4.000 |
| Canada | 7d5ef554-26fd-4d8f-b2bc-310782a00970 | SRR2736253 | SRR2736253 | 0.008 | 0.008 | 0.250 | 0.500 | 2.000 | 4.000 | 0.015 | 0.016 | 1.000 | 2.000 |
| Canada | 805d70b1-a3fe-45c0-9cf9-93eeebeb4eb4 | SRR2736212 | SRR2736212 | 0.032 | 0.016 | 0.500 | 0.500 | 8.000 | 4.000 | 0.016 | 0.016 | 2.000 | 2.000 |
| Canada | 80cf80aa-e4b5-424b-a622-5c175e457be4 | SRR2736180 | SRR2736180 | 0.008 | 0.008 | 0.250 | 0.250 | 4.000 | 4.000 | 0.015 | 0.016 | 1.000 | 1.000 |
| Canada | 8175dafc-5ffb-4dd7-b170-13b35f7f5c8d | SRR2736205 | SRR2736205 | 0.032 | 0.031 | 2.000 | 2.000 | 8.000 | 4.000 | 16.000 | 16.000 | 4.000 | 4.000 |
| Canada | 81c38d06-1c6a-41b7-a396-604a43851606 | SRR2736166 | SRR2736166 | 0.016 | 0.016 | 4.000 | 2.000 | 8.000 | 8.000 | 16.000 | 16.000 | 4.000 | 4.000 |
| Canada | 847bf895-1d20-400b-ad21-76f3d7f2dc05 | SRR2736239 | SRR2736239 | |  |  |  | 2.000 | 2.000 | 0.015 | 0.016 | 2.000 | 2.000 |
| Canada | 84ee58c2-5036-40f2-a0f9-081052b3f20f | SRR2736206 | SRR2736206 | 0.032 | 0.008 | 0.250 | 0.250 | 16.000 | 8.000 | 0.015 | 0.016 | 2.000 | 2.000 |
| Canada | 8afc3675-c4f3-4220-b70d-849bec010ec0 | SRR2736136 | SRR2736136 | 0.032 | 0.016 | 0.500 | 0.500 | 4.000 | 8.000 | 0.015 | 0.016 | 2.000 | 1.000 |
| Canada | 8c45b996-011a-472f-8f09-20650b3babd4 | SRR2736261 | SRR2736261 | 0.032 | 0.016 | 4.000 | 2.000 | 2.000 | 1.000 | 0.016 | 0.016 | 4.000 | 4.000 |
| Canada | 8ca73c32-9f75-4ea1-9035-85a04b7c42b1 | SRR2736199 | SRR2736199 | 0.125 | 0.125 | 2.000 | 1.000 | 8.000 | 4.000 | 16.000 | 16.000 | 2.000 | 2.000 |
| Canada | 8e423b30-ad7b-458a-a51f-287c80cca084 | SRR2736299 | SRR2736299 | 0.032 | 0.008 | 0.125 | 0.250 | 8.000 | 8.000 | 0.015 | 0.016 | 2.000 | 2.000 |
| Canada | 8e9c3c9b-afa7-4773-88db-8ecb2785cc48 | SRR2736219 | SRR2736219 | 0.016 | 0.008 | 0.500 | 0.250 | 8.000 | 8.000 | 0.015 | 0.016 | 2.000 | 2.000 |
| Canada | 8f8130b0-0685-43e9-8b04-606a2e60834c | SRR2736132 | SRR2736132 | 0.016 | 0.016 | 2.000 | 2.000 | 4.000 | 8.000 | 0.016 | 0.016 | 2.000 | 4.000 |
| Canada | 92046845-2030-4f62-b6fb-898f29e0b336 | SRR2736260 | SRR2736260 | 0.250 | 0.125 | 8.000 | 2.000 | 2.000 | 4.000 | 16.000 | 16.000 | 4.000 | 4.000 |
| Canada | 9299386e-5588-4250-a183-d7bedca348c7 | SRR2736263 | SRR2736263 | 0.125 | 0.125 | 4.000 | 2.000 | 8.000 | 8.000 | 16.000 | 16.000 | 8.000 | 4.000 |
| Canada | 93693da4-b2a3-43e0-8c66-e638427f2b2b | SRR1661199 | SRR1661199 | 0.032 | 0.016 | 2.000 | 1.000 | 0.500 | 1.000 | 4.000 | 16.000 | 2.000 | 2.000 |
| Canada | 99c570c5-9166-4efe-9a25-449ecf51ed83 | SRR2736157 | SRR2736157 | 0.032 | 0.016 | 2.000 | 2.000 | 32.000 | 8.000 | 32.000 | 16.000 | 4.000 | 4.000 |
| Canada | 99cedf90-6f04-464b-b6b5-adfe24eb1ac2 | SRR2736227 | SRR2736227 | 0.125 | 0.125 | 2.000 | 2.000 | 8.000 | 8.000 | 16.000 | 16.000 | 4.000 | 4.000 |
| Canada | 9a10de39-7a8d-43ab-ae85-fea609596471 | SRR1661243 | SRR1661243 | 0.016 | 0.031 | 2.000 | 2.000 | 1.000 | 1.000 | 16.000 | 16.000 | 4.000 | 4.000 |
| Canada | 9a98816c-626c-45cc-b521-e746cd96d1f3 | SRR2736150 | SRR2736150 | 0.125 | 0.125 | 2.000 | 2.000 | 0.500 | 1.000 | 16.000 | 16.000 | 4.000 | 4.000 |
| Canada | 9ad73c18-eba9-4a47-912c-5e976ab2ffb2 | SRR1661167 | SRR1661167 | 0.063 | 0.016 | 8.000 | 2.000 | 2.000 | 1.000 | 0.032 | 0.016 | 4.000 | 4.000 |
| Canada | 9c351b3d-37ba-41d2-8734-c283f08cdaf4 | SRR2736109 | SRR2736109 | 0.008 | 0.016 | 0.500 | 2.000 | 2.000 | 1.000 | 0.015 | 0.016 | 2.000 | 4.000 |
| Canada | 9cdf9db4-adea-4465-a151-bc1039f07835 | SRR2736193 | SRR2736193 | 0.016 | 0.016 | 1.000 | 1.000 | 8.000 | 2.000 | 4.000 | 16.000 | 2.000 | 2.000 |
| Canada | 9d6d9db0-7ef4-48b0-882d-aa33487aad5e | SRR1661207 | SRR1661207 | 0.125 | 0.016 | 256.000 | 64.000 | 512.000 | 512.000 | 4.000 | 2.000 | 64.000 | 32.000 |
| Canada | a072db5c-a1ce-430c-b1a5-c9abb35c372e | SRR2736144 | SRR2736144 | 0.032 | 0.016 | 0.500 | 0.500 | 8.000 | 8.000 | 0.015 | 0.016 | 2.000 | 1.000 |
| Canada | a4eb095f-2195-4953-8058-90faf75a616c | SRR2736182 | SRR2736182 | 0.032 | 0.008 | 0.250 | 0.250 | 4.000 | 4.000 | 0.015 | 0.016 | 1.000 | 1.000 |
| Canada | a5945c5f-608e-4acb-918c-af69b4420a17 | SRR2736297 | SRR2736297 | 0.016 | 0.008 | 0.125 | 0.250 | 8.000 | 8.000 | 0.015 | 0.016 | 1.000 | 2.000 |
| Canada | a6a54d33-a2a6-475d-b82c-15e9a19e1679 | SRR1661322 | SRR1661322 | 0.125 | 0.031 | 16.000 | 2.000 | 0.500 | 1.000 | 0.032 | 0.016 | 8.000 | 4.000 |
| Canada | a6fab132-881d-4070-af30-4d76fee7f5b5 | SRR1661223 | SRR1661223 | |  |  |  | 0.500 | 1.000 | 0.125 | 2.000 | 1.000 | 2.000 |
| Canada | a9204c17-1889-4af6-8cb4-7fad9cc096c2 | SRR2736258 | SRR2736258 | 0.063 | 0.125 | 4.000 | 2.000 | 16.000 | 8.000 | 16.000 | 16.000 | 4.000 | 4.000 |
| Canada | a926e632-fe29-4275-8737-63036ee3b9ab | SRR2736126 | SRR2736126 | |  |  |  | 2.000 | 1.000 | 0.032 | 0.016 | 4.000 | 4.000 |
| Canada | a97ff5ba-28e3-4dba-84b1-4c7bf82c26d3 | SRR2736121 | SRR2736121 | 0.016 | 0.016 | 2.000 | 2.000 | 2.000 | 1.000 | 0.016 | 0.016 | 4.000 | 4.000 |
| Canada | ab59c737-2642-4cdc-98f8-8ba22a0a47ff | SRR2736143 | SRR2736143 | 0.032 | 0.031 | 8.000 | 2.000 | 4.000 | 8.000 | 16.000 | 16.000 | 4.000 | 4.000 |
| Canada | ac5beca5-cef0-48ae-aeb7-2fd1138aac5b | SRR2736255 | SRR2736255 | 0.032 | 0.016 | 2.000 | 1.000 | 2.000 | 2.000 | 0.032 | 0.016 | 8.000 | 4.000 |
| Canada | ac74a4b1-16fe-4dbd-8aad-b2611c59ccd7 | SRR2736234 | SRR2736234 | 0.032 | 0.008 | 0.250 | 0.250 | 8.000 | 8.000 | 0.015 | 0.016 | 2.000 | 2.000 |
| Canada | ac90870c-8710-4bf6-87c4-9801ecdfd55c | SRR2736246 | SRR2736246 | 0.125 | 0.125 | 2.000 | 2.000 | 8.000 | 8.000 | 16.000 | 16.000 | 4.000 | 4.000 |
| Canada | b1927177-fbdf-474e-97cc-beb1c5b49917 | SRR1661315 | SRR1661315 | 0.125 | 0.125 | 4.000 | 1.000 | 8.000 | 8.000 | 16.000 | 16.000 | 4.000 | 4.000 |
| Canada | b1fc8a28-c803-4e58-a9fc-5d28c0458b45 | SRR2736271 | SRR2736271 | 0.016 | 0.008 | 0.500 | 0.500 | 4.000 | 2.000 | 0.015 | 0.016 | 4.000 | 2.000 |
| Canada | b250615b-b664-432b-bb6e-1563b147deba | SRR2736183 | SRR2736183 | 0.008 | 0.008 | 0.250 | 0.250 | 4.000 | 4.000 | 0.015 | 0.016 | 1.000 | 1.000 |
| Canada | b3218409-0b0f-4974-b030-947048bb00b0 | SRR2736240 | SRR2736240 | 0.008 | 0.008 | 0.250 | 0.250 | 8.000 | 8.000 | 0.015 | 0.016 | 2.000 | 2.000 |
| Canada | b3f2faa1-4da0-489f-a554-1acb68c7bbc1 | SRR2736108 | SRR2736108 | 0.008 | 0.016 | 1.000 | 2.000 | 2.000 | 1.000 | 0.015 | 0.016 | 2.000 | 4.000 |
| Canada | b51e4b43-30dc-4b9d-81c0-6c6eb0de04ce | SRR1661327 | WHO-L | 0.250 | 0.063 | 2.000 | 4.000 | 0.500 | 0.500 | 32.000 | 8.000 | 4.000 | 2.000 |
| Canada | b58aa115-03e1-476e-9fd7-66ff94e11806 | SRR2736264 | SRR2736264 | 0.125 | 0.125 | 2.000 | 2.000 | 16.000 | 8.000 | 16.000 | 16.000 | 4.000 | 4.000 |
| Canada | b6ce2a9d-f2a5-4463-9779-ced8dac0c15b | SRR2736133 | SRR2736133 | |  |  |  | 4.000 | 8.000 | 0.015 | 0.016 | 1.000 | 1.000 |
| Canada | b792daa4-8686-47ae-8b49-d947be23293d | SRR2736273 | SRR2736273 | 0.032 | 0.063 | 1.000 | 1.000 | 8.000 | 8.000 | 16.000 | 8.000 | 2.000 | 4.000 |
| Canada | b89267e0-5a16-4533-b494-942a7024fa06 | SRR2736185 | SRR2736185 | 0.032 | 0.031 | 2.000 | 2.000 | 2.000 | 1.000 | 16.000 | 16.000 | 8.000 | 4.000 |
| Canada | baa1b80b-3939-4595-a468-b686f5cf4577 | SRR2736210 | SRR2736210 | 0.016 | 0.008 | 0.250 | 0.250 | 4.000 | 4.000 | 0.015 | 0.016 | 2.000 | 2.000 |
| Canada | baf21ab6-c077-4277-abaf-943b7e5f0f5c | SRR2736290 | SRR2736290 | 0.016 | 0.016 | 1.000 | 1.000 | 4.000 | 2.000 | 0.016 | 0.016 | 4.000 | 4.000 |
| Canada | bbac69c1-d634-40f5-be13-5a24f96d2f08 | SRR1661250 | SRR1661250 | 0.125 | 0.125 | 1.000 | 2.000 | 1.000 | 1.000 | 16.000 | 16.000 | 2.000 | 4.000 |
| Canada | bd0f399b-eaa3-4b8b-ae90-f1df6a685545 | SRR2736129 | SRR2736129 | 0.016 | 0.016 | 2.000 | 2.000 | 8.000 | 4.000 | 0.016 | 0.016 | 4.000 | 4.000 |
| Canada | bd30df50-20c3-4bbf-bb01-11efd75a9a91 | SRR1661329 | WHO-N | 0.008 | 0.008 | 8.000 | 8.000 | 0.125 | 0.500 | 4.000 | 16.000 | 16.000 | 16.000 |
| Canada | bdae066b-9e48-4932-bcf0-289dc441f29b | SRR2736105 | SRR2736105 | 0.032 | 0.031 | 2.000 | 2.000 | 2.000 | 1.000 | 16.000 | 8.000 | 8.000 | 4.000 |
| Canada | beb2ab62-f31f-4dee-9e03-1433b46b2a7d | SRR2736204 | SRR2736204 | 0.032 | 0.016 | 2.000 | 1.000 | 4.000 | 4.000 | 8.000 | 16.000 | 2.000 | 2.000 |
| Canada | bf8bfe56-9ee7-42f5-9913-0d791518a71c | SRR2736125 | SRR2736125 | 0.016 | 0.016 | 1.000 | 2.000 | 2.000 | 1.000 | 0.032 | 0.016 | 4.000 | 4.000 |
| Canada | bff3c312-9285-4621-a9e2-55b5b89e2400 | SRR2736288 | SRR2736288 | 0.016 | 0.008 | 0.250 | 0.250 | 8.000 | 8.000 | 0.015 | 0.016 | 1.000 | 2.000 |
| Canada | c336a17f-acb2-4147-8d6d-dad031059443 | SRR2736120 | SRR2736120 | 0.016 | 0.016 | 2.000 | 2.000 | 2.000 | 1.000 | 0.016 | 0.016 | 4.000 | 4.000 |
| Canada | c395ae79-938a-47ab-855c-430429a6b459 | SRR2736300 | SRR2736300 | 0.008 | 0.008 | 0.125 | 0.250 | 8.000 | 8.000 | 0.015 | 0.016 | 2.000 | 2.000 |
| Canada | c4c6ebec-b4f4-44e9-b60a-8eb1970bb50e | SRR2736130 | SRR2736130 | 0.016 | 0.016 | 0.500 | 0.500 | 4.000 | 8.000 |  |  | 1.000 | 1.000 |
| Canada | c5ad3930-afd6-4804-b33a-1a1a08aae74c | SRR1661331 | WHO-P | 0.008 | 0.008 | 0.250 | 0.250 | 2.000 | 1.000 | 0.015 | 0.016 | 0.500 | 2.000 |
| Canada | c6ec2ee4-404f-44d3-9b9f-12d2e651244b | SRR2736283 | SRR2736283 | 0.008 | 0.008 | 0.125 | 0.250 | 8.000 | 8.000 | 0.015 | 0.016 | 1.000 | 2.000 |
| Canada | c8c7db0b-7261-4a82-84e3-2000c87b5559 | SRR2736266 | SRR2736266 | 0.063 | 0.125 | 2.000 | 2.000 | 4.000 | 4.000 | 16.000 | 16.000 | 4.000 | 4.000 |
| Canada | c8f66891-2863-4d49-a009-98507db36977 | SRR2736161 | SRR2736161 | 0.125 | 0.125 | 4.000 | 2.000 | 16.000 | 8.000 | 16.000 | 16.000 | 4.000 | 4.000 |
| Canada | c8fa5bb8-31fd-4c6f-9308-d45ac8072515 | SRR2736225 | SRR2736225 | 0.016 | 0.016 | 0.500 | 0.500 | 16.000 | 8.000 | 0.016 | 0.016 | 2.000 | 2.000 |
| Canada | c9fba687-4f60-4f5e-860b-4e937a9aa12b | SRR2736236 | SRR2736236 | 0.008 | 0.008 | 0.250 | 0.250 | 8.000 | 8.000 | 0.015 | 0.016 | 2.000 | 2.000 |
| Canada | cb32bfc6-84e0-41fe-ae91-bf30758fb11d | SRR2736196 | SRR2736196 | 0.004 | 0.008 | 0.250 | 0.250 | 8.000 | 8.000 | 0.015 | 0.016 | 1.000 | 2.000 |
| Canada | cb48ea4f-2692-4fe6-9b5f-cae610b3d8f2 | SRR2736304 | SRR2736304 | 0.016 | 0.008 | 1.000 | 0.500 | 2.000 | 2.000 | 0.015 | 0.016 | 4.000 | 2.000 |
| Canada | cc09c31d-108a-4f0a-9711-90a41dcec46d | SRR2736106 | SRR2736106 | 0.032 | 0.016 | 4.000 | 2.000 | 2.000 | 1.000 | 0.016 | 0.016 | 8.000 | 4.000 |
| Canada | cd8e4de0-33b5-454f-802e-c7fc5f375301 | SRR2736208 | SRR2736208 | 0.016 | 0.008 | 0.500 | 0.250 | 16.000 | 8.000 | 0.015 | 0.016 | 1.000 | 2.000 |
| Canada | cea504b5-5cad-437b-a0f8-e87702abdcda | SRR2736097 | SRR2736097 | 0.032 | 0.016 | 4.000 | 2.000 | 2.000 | 1.000 | 0.016 | 0.016 | 4.000 | 4.000 |
| Canada | d01ab949-5237-4fc3-97f5-c974821c1a24 | SRR2736158 | SRR2736158 | 0.032 | 0.031 | 4.000 | 2.000 | 8.000 | 8.000 |  |  | 4.000 | 4.000 |
| Canada | d15613e2-cc65-4d4c-a279-9a85a5169e2d | SRR2736093 | SRR2736093 | 0.004 | 0.016 | 2.000 | 2.000 | 2.000 | 1.000 | 0.015 | 0.016 | 2.000 | 4.000 |
| Canada | d222790d-93d9-4f39-ad17-92bef3f6f39b | SRR1661227 | SRR1661227 | 0.063 | 0.031 | 4.000 | 2.000 | 512.000 | 512.000 | 32.000 | 16.000 | 4.000 | 4.000 |
| Canada | d222c927-72a9-47da-9ea0-33307cf21dc9 | SRR1661183 | SRR1661183 | 0.032 | 0.031 | 2.000 | 2.000 | 1.000 | 1.000 | 16.000 | 16.000 | 4.000 | 4.000 |
| Canada | d2ac17ff-b445-48e5-9caf-c860c347c159 | SRR1661153 | SRR1661153 | 0.063 | 0.031 | 4.000 | 2.000 | 0.500 | 1.000 | 0.016 | 0.016 | 2.000 | 4.000 |
| Canada | d2bdd39f-08d6-4ea2-95df-3e6f22f3df29 | SRR2736176 | SRR2736176 | 0.004 | 0.008 | 0.250 | 0.250 | 2.000 | 4.000 | 0.015 | 0.016 | 1.000 | 1.000 |
| Canada | d2fdfad2-0341-4a74-bdbd-2dc4fbffb1cb | SRR2736111 | SRR2736111 | 0.008 | 0.016 | 0.250 | 0.500 | 4.000 | 4.000 | 0.015 | 0.016 | 2.000 | 1.000 |
| Canada | d3482a5c-57d7-4c53-a419-b75c2d353fd6 | SRR2736215 | SRR2736215 | 0.063 | 0.063 | 2.000 | 4.000 | 8.000 | 8.000 | 8.000 | 8.000 | 4.000 | 4.000 |
| Canada | d38cc14b-8f4e-48d2-9668-e02ba93ba802 | SRR2736298 | SRR2736298 | 0.016 | 0.008 | 0.500 | 0.500 | 2.000 | 2.000 | 0.015 | 0.016 | 2.000 | 2.000 |
| Canada | d398bc7f-d53e-4f84-bf80-e4168fa8a4c5 | SRR2736265 | SRR2736265 | 0.125 | 0.125 | 2.000 | 2.000 | 8.000 | 8.000 | 16.000 | 16.000 | 4.000 | 4.000 |
| Canada | d413f7a9-c370-4c23-aec7-24dbeac3a466 | SRR2736187 | SRR2736187 | 0.016 | 0.008 | 0.125 | 0.250 | 8.000 | 4.000 | 0.015 | 0.016 | 1.000 | 1.000 |
| Canada | d599cdd8-570c-4df2-b9d3-25aec0200d20 | SRR2736269 | SRR2736269 | 0.032 | 0.008 | 0.500 | 0.500 | 2.000 | 2.000 | 0.015 | 0.016 | 2.000 | 2.000 |
| Canada | d6463d5e-2322-42d4-9cf9-f1339ae57144 | SRR2736118 | SRR2736118 | 0.008 | 0.031 | 0.500 | 0.500 | 1.000 | 0.500 | 0.015 | 0.016 | 2.000 | 1.000 |
| Canada | d8371c2e-6565-469e-8004-c2971dfa9648 | SRR1661242 | SRR1661242 | 0.250 | 0.125 | 2.000 | 2.000 | 0.500 | 1.000 |  |  | 4.000 | 4.000 |
| Canada | d896559b-f601-48f4-b49d-3a96b9374f85 | SRR2736189 | SRR2736189 | 0.016 | 0.008 | 0.125 | 0.250 | 8.000 | 8.000 | 0.015 | 0.016 | 1.000 | 2.000 |
| Canada | d89bf4d5-1c3d-422b-b240-37651136968d | SRR2736224 | SRR2736224 | 0.125 | 0.125 | 4.000 | 2.000 | 8.000 | 8.000 | 16.000 | 16.000 | 4.000 | 4.000 |
| Canada | d948b2f7-f973-45ae-8124-58ce362efcd5 | SRR2736231 | SRR2736231 | 0.016 | 0.031 | 1.000 | 2.000 | 8.000 | 8.000 | 16.000 | 16.000 | 4.000 | 4.000 |
| Canada | d9bf6ec0-9a04-4148-97cc-74fa4646697f | SRR2736221 | SRR2736221 | 0.063 | 0.008 | 0.500 | 0.250 | 16.000 | 8.000 | 0.015 | 0.016 | 2.000 | 2.000 |
| Canada | dcc69ca8-e75c-4650-a557-d75d5d74e1f7 | SRR2736155 | SRR2736155 | 0.063 | 0.031 | 4.000 | 2.000 | 4.000 | 2.000 | 16.000 | 16.000 | 8.000 | 64.000 |
| Canada | df059529-7bd9-4428-97db-eb568584631e | SRR1661279 | SRR1661279 | 0.250 | 0.125 | 4.000 | 2.000 | 2.000 | 4.000 | 16.000 | 16.000 | 4.000 | 4.000 |
| Canada | e077bee0-db2a-41f6-b0bf-12acf2b9fac0 | SRR2736173 | SRR2736173 | 0.008 | 0.008 | 0.500 | 0.250 | 4.000 | 4.000 | 0.015 | 0.016 | 1.000 | 1.000 |
| Canada | e50385ee-abe5-4728-8146-193821b25110 | SRR2736145 | SRR2736145 | 0.032 | 0.016 | 0.500 | 0.500 | 8.000 | 8.000 | 0.015 | 0.016 | 2.000 | 1.000 |
| Canada | e7ab9469-7cb5-4144-8fd3-818ab30c8b54 | SRR2736248 | SRR2736248 | 0.063 | 0.125 | 2.000 | 2.000 | 8.000 | 8.000 | 16.000 | 16.000 | 4.000 | 4.000 |
| Canada | e849deb0-e253-4d14-96b6-9f5907e0ec9d | SRR2736226 | SRR2736226 | 0.125 | 0.125 | 2.000 | 2.000 | 8.000 | 8.000 | 16.000 | 16.000 | 8.000 | 4.000 |
| Canada | e91f666a-c83a-49e7-8af5-4f8624424ff6 | SRR2736228 | SRR2736228 | 0.250 | 0.125 | 4.000 | 2.000 | 8.000 | 8.000 | 16.000 | 16.000 | 8.000 | 4.000 |
| Canada | eab481fc-4ac8-4bb6-a88f-7c03087df512 | SRR2736229 | SRR2736229 | 0.250 | 0.125 | 2.000 | 2.000 | 8.000 | 8.000 | 16.000 | 16.000 | 8.000 | 4.000 |
| Canada | ebb74cb6-d7a3-44e2-b766-d00ac3460ab3 | SRR2736243 | SRR2736243 | 0.063 | 0.063 | 2.000 | 1.000 | 8.000 | 4.000 | 16.000 | 8.000 | 4.000 | 4.000 |
| Canada | ebdce0d9-bd2d-4d48-ac41-ba61197e7f27 | SRR2736250 | SRR2736250 | 0.008 | 0.008 | 0.250 | 0.250 | 8.000 | 8.000 | 0.015 | 0.016 | 2.000 | 2.000 |
| Canada | ebf44a0a-2a34-46c1-9988-129300724c39 | SRR2736107 | SRR2736107 | 0.008 | 0.016 | 1.000 | 2.000 | 2.000 | 1.000 | 0.016 | 0.016 | 4.000 | 4.000 |
| Canada | ec6a38a4-8630-414f-90f2-7ad65c88da7b | SRR2736149 | SRR2736149 | 0.032 | 0.063 | 256.000 | 128.000 | 2.000 | 1.000 | 16.000 | 16.000 | 64.000 | 16.000 |
| Canada | eeb78e1d-744d-4cbb-aa12-fc0fd2d0bc6e | SRR2736257 | SRR2736257 | 0.032 | 0.063 | 2.000 | 1.000 | 8.000 | 8.000 | 8.000 | 8.000 | 4.000 | 4.000 |
| Canada | eee3cc7e-93ed-468d-92ed-7cd6a961b9d1 | SRR2736245 | SRR2736245 | 0.125 | 0.125 | 1.000 | 2.000 | 8.000 | 8.000 | 16.000 | 16.000 | 4.000 | 4.000 |
| Canada | ef0b4bbb-511c-4066-b839-64247026f422 | SRR1661328 | WHO-M | 0.008 | 0.031 | 8.000 | 128.000 | 0.250 | 1.000 | 2.000 | 2.000 | 1.000 | 4.000 |
| Canada | f186b8f9-75de-4b5e-97c6-10c85e302a27 | SRR2736303 | SRR2736303 | 0.008 | 0.008 | 0.250 | 0.250 | 8.000 | 8.000 | 0.015 | 0.016 | 1.000 | 2.000 |
| Canada | f2eea16c-726d-4f4b-9745-d4524fbfada6 | SRR2736238 | SRR2736238 | 0.008 | 0.008 | 0.250 | 0.250 | 2.000 | 2.000 | 0.015 | 0.016 | 2.000 | 2.000 |
| Canada | f307b003-7e7a-426a-8edc-29a4c89bf2fe | SRR2736113 | SRR2736113 | 0.016 | 0.016 | 1.000 | 2.000 | 2.000 | 1.000 | 0.016 | 0.016 | 4.000 | 4.000 |
| Canada | f37663b5-9e0c-42a7-b740-c8e9fa4810c8 | SRR2736170 | SRR2736170 | 0.008 | 0.008 | 0.500 | 0.250 | 4.000 | 4.000 | 0.015 | 0.016 | 1.000 | 1.000 |
| Canada | f488ca52-89d3-4d36-8b52-5304e7584bcd | SRR1661325 | WHO-G | 0.008 | 0.008 | 0.500 | 4.000 | 0.250 | 0.250 | 0.125 | 0.125 | 32.000 | 16.000 |
| Canada | f507c8f0-295e-4559-83b1-4ec64e3ea549 | SRR1661281 | SRR1661281 | 0.032 | 0.008 | 0.500 | 0.250 | 1.000 | 1.000 | 0.015 | 0.016 | 1.000 | 2.000 |
| Canada | f71e71c6-4c95-487e-b0a0-e5857b14e28d | SRR2736296 | SRR2736296 | 0.008 | 0.008 | 0.125 | 0.250 | 8.000 | 8.000 | 0.015 | 0.016 | 1.000 | 2.000 |
| Canada | f7cfe580-6466-42eb-8d74-d2272f199c29 | SRR1661155 | SRR1661155 | 0.125 | 0.016 | 2.000 | 2.000 | 1.000 | 1.000 | 0.016 | 0.016 | 4.000 | 4.000 |
| Canada | f8207e17-76fd-43f1-bdbf-1cf90144ae0c | SRR2736216 | SRR2736216 | 0.063 | 0.063 | 2.000 | 4.000 | 8.000 | 8.000 | 8.000 | 8.000 | 4.000 | 4.000 |
| Canada | f8b755e6-5913-4376-ab2d-545acf2c7bcb | SRR2736162 | SRR2736162 | 0.125 | 0.125 | 2.000 | 2.000 | 16.000 | 8.000 | 16.000 | 16.000 | 4.000 | 4.000 |
| Canada | f8c35e30-af1b-4f8e-8a6d-6e77bf947699 | SRR1661249 | SRR1661249 | 0.125 | 0.125 | 2.000 | 2.000 | 1.000 | 1.000 | 16.000 | 16.000 | 2.000 | 4.000 |
| Canada | f9771a65-9ed0-445b-bf35-c8035e6fc1ec | SRR2736270 | SRR2736270 | 0.032 | 0.031 | 0.500 | 2.000 | 0.063 | 2.000 | 4.000 | 16.000 | 1.000 | 4.000 |
| Canada | fa190eeb-3978-4042-ae07-cf62a1492234 | SRR2736117 | SRR2736117 | 0.032 | 0.016 | 2.000 | 2.000 | 2.000 | 1.000 | 0.016 | 0.016 | 4.000 | 4.000 |
| Canada | fa4c9e75-0b8c-4112-a2d6-6051cbcb0020 | SRR2736123 | SRR2736123 | 0.016 | 0.016 | 2.000 | 2.000 | 2.000 | 1.000 | 0.016 | 0.016 | 4.000 | 4.000 |
| Canada | fb48759d-b1cf-418d-9d75-a6a3e4a031a5 | SRR2736190 | SRR2736190 | 0.004 | 0.008 | 0.125 | 0.250 | 8.000 | 8.000 | 0.015 | 0.016 | 1.000 | 2.000 |
| Canada | fb49583a-24bf-42e4-b767-60ff8ff7d7eb | SRR1661292 | SRR1661292 | 4.000 | 0.125 | 2.000 | 2.000 | 0.500 | 1.000 | 16.000 | 16.000 | 4.000 | 4.000 |
| Canada | ff50db8d-3afc-477c-8a56-6318bf0a77c7 | SRR2736141 | SRR2736141 | 0.016 | 0.031 | 2.000 | 2.000 | 8.000 | 8.000 | 16.000 | 16.000 | 2.000 | 4.000 |
| Canada | ff733012-d5b5-4caf-9ad6-d12d6bdf9231 | SRR2736101 | SRR2736101 | 0.032 | 0.016 | 2.000 | 2.000 | 2.000 | 1.000 | 0.016 | 0.016 | 8.000 | 4.000 |
| USA | 013e45ce-2174-4ffd-acbf-1bf96326e49f | ERR191797 | ERR191797 | 0.015 | 0.031 | 1.000 | 0.500 | 0.500 | 0.500 | 8.000 | 16.000 | 2.000 | 2.000 |
| USA | 01db320e-3865-4876-a96f-be02604986ca | ERR223665 | ERR223665 | 0.250 | 0.250 | 2.000 | 2.000 | 0.500 | 0.500 | 8.000 | 16.000 | 2.000 | 2.000 |
| USA | 03847ff8-0aca-453e-8ae9-74b0d12a750f | ERR223654 | ERR223654 | 0.030 | 0.031 | 2.000 | 2.000 | 0.500 | 0.500 | 4.000 | 16.000 | 1.000 | 1.000 |
| USA | 06a95186-7632-4ea3-8422-81a2cef368ab | ERR223691 | ERR223691 | 0.250 | 0.250 | 2.000 | 2.000 | 0.250 | 0.500 | 16.000 | 16.000 | 2.000 | 2.000 |
| USA | 0812811b-e50d-4fcb-bc0d-90970782b024 | ERR223609 | ERR223609 | 0.250 | 0.250 | 2.000 | 2.000 | 0.500 | 0.500 | 16.000 | 16.000 | 4.000 | 2.000 |
| USA | 090113db-3851-4010-a459-b3e42011e8c3 | ERR191774 | ERR191774 | 0.250 | 0.250 | 2.000 | 2.000 | 1.000 | 0.500 | 16.000 | 16.000 | 2.000 | 2.000 |
| USA | 0a82afc4-ee68-4907-b3f2-558e51e1fb8e | ERR223667 | ERR223667 | 0.250 | 0.250 | 0.500 | 1.000 | 0.500 | 0.500 | 0.015 | 0.016 | 0.500 | 1.000 |
| USA | 0d1102a0-0e2d-45a5-b56e-4f0aa74bacd3 | ERR191776 | ERR191776 | 0.250 | 0.250 | 4.000 | 2.000 | 1.000 | 0.500 | 16.000 | 16.000 | 4.000 | 2.000 |
| USA | 0ead445f-e8a9-4794-bfad-72a20f76bbf2 | ERR191764 | ERR191764 | 0.250 | 0.250 | 4.000 | 2.000 | 1.000 | 0.500 | 32.000 | 16.000 | 4.000 | 2.000 |
| USA | 0fafc6eb-ed61-4033-a684-4cc9609bfccb | ERR223611 | ERR223611 | 0.250 | 0.250 | 1.000 | 2.000 | 0.500 | 0.500 | 16.000 | 16.000 | 2.000 | 2.000 |
| USA | 1085d3eb-19b5-4cf9-95d7-4181dad3fde8 | ERR223620 | ERR223620 | 0.015 | 0.031 | 0.250 | 1.000 | 0.125 | 0.500 | 2.000 | 16.000 | 0.500 | 2.000 |
| USA | 129cfee2-f8a4-4dd2-b9c5-4598375c8c05 | ERR223681 | ERR223681 | 0.250 | 0.250 | 1.000 | 1.000 | 0.500 | 0.500 | 0.015 | 0.016 | 1.000 | 1.000 |
| USA | 17be866b-dc42-426c-9782-c4605745ed28 | ERR223692 | ERR223692 | 0.030 | 0.031 | 8.000 | 8.000 | 0.250 | 0.250 | 2.000 | 16.000 | 64.000 | 16.000 |
| USA | 1883c75e-7805-456e-81d2-e9753cfaf419 | ERR191800 | ERR191800 | 0.250 | 0.250 | 0.500 | 1.000 | 0.500 | 0.500 | 0.015 | 0.016 | 1.000 | 1.000 |
| USA | 19bddd3d-accd-49b2-99c5-dd319f9cd927 | ERR191775 | ERR191775 | 0.060 | 0.250 | 4.000 | 2.000 | 1.000 | 0.500 | 16.000 | 16.000 | 4.000 | 2.000 |
| USA | 1a208729-95c9-43f3-9768-24166661df99 | ERR191811 | ERR191811 | 0.060 | 0.063 | 2.000 | 2.000 | 0.250 | 0.250 | 16.000 | 16.000 | 2.000 | 1.000 |
| USA | 1b0abceb-4ff6-4ea9-b510-6550f04c3f1a | ERR191767 | ERR191767 | 0.015 | 0.016 | 0.500 | 0.500 | 8.000 | 16.000 | 0.015 | 0.016 | 1.000 | 1.000 |
| USA | 1b0dae5f-f45c-4879-a9f3-d7379f80305d | ERR223680 | ERR223680 | 0.030 | 0.016 | 0.250 | 0.250 | 16.000 | 8.000 | 0.015 | 0.016 | 1.000 | 1.000 |
| USA | 1ce6d1cf-3b82-421e-9fd4-b45a07bcc361 | ERR191793 | ERR191793 | 0.060 | 0.031 | 4.000 | 1.000 | 1.000 | 0.500 | 0.015 | 0.016 | 32.000 | 32.000 |
| USA | 1f5ecae9-3ea2-4abd-9cf0-67ec23cc2335 | ERR191730 | ERR191730 | 0.250 | 0.250 | 4.000 | 2.000 | 1.000 | 0.500 | 16.000 | 16.000 | 4.000 | 2.000 |
| USA | 202e7856-1ef4-4673-9099-cb38b285048f | ERR191753 | ERR191753 | 0.060 | 0.063 | 16.000 | 16.000 | 0.500 | 0.500 | 4.000 | 2.000 | 16.000 | 32.000 |
| USA | 238ade2c-bfa5-4135-8612-50a57af72a75 | ERR191792 | ERR191792 | 0.250 | 0.250 | 2.000 | 2.000 | 0.500 | 0.500 | 16.000 | 16.000 | 4.000 | 2.000 |
| USA | 23ae0ef6-d5d0-4003-a59d-2bb16544e969 | ERR191761 | ERR191761 | 0.030 | 0.031 | 4.000 | 2.000 | 0.500 | 0.500 | 16.000 | 16.000 | 2.000 | 2.000 |
| USA | 28ba3c44-c524-418b-882a-cb7fe731a4bf | ERR191768 | ERR191768 | 0.250 | 0.250 | 4.000 | 2.000 | 1.000 | 0.500 | 16.000 | 16.000 | 4.000 | 2.000 |
| USA | 2b0d54ff-714c-4203-9c01-586e2d38eabd | ERR223651 | ERR223651 | 0.250 | 0.250 | 4.000 | 2.000 | 1.000 | 0.500 | 16.000 | 16.000 | 4.000 | 2.000 |
| USA | 2d08ee08-e0a0-4b90-a06c-dc1d49ba88d1 | ERR191747 | ERR191747 | 0.015 | 0.016 | 0.250 | 0.250 | 8.000 | 8.000 | 0.015 | 0.016 | 1.000 | 1.000 |
| USA | 2d370f82-2bad-4ea0-9672-571488bbf44f | ERR191732 | ERR191732 | 0.250 | 0.250 | 4.000 | 2.000 | 1.000 | 0.500 | 16.000 | 16.000 | 4.000 | 2.000 |
| USA | 2e569f7b-0715-4c8a-91a3-28f5056f03cc | ERR191751 | ERR191751 | 0.060 | 0.031 | 4.000 | 2.000 | 0.500 | 0.500 | 16.000 | 16.000 | 4.000 | 2.000 |
| USA | 2e7680c4-1256-4dda-80d4-313c23a58b91 | ERR191819 | ERR191819 | 0.015 | 0.031 | 8.000 | 4.000 | 0.250 | 0.250 | 1.000 | 16.000 | 32.000 | 16.000 |
| USA | 2fd9d0d8-7c44-4b3e-994e-351ae65dff8d | ERR191812 | ERR191812 | 0.250 | 0.031 | 2.000 | 0.500 | 0.500 | 0.500 | 0.030 | 0.016 | 2.000 | 2.000 |
| USA | 306a4d46-50cb-4768-a59d-2af1a7a9c1ca | ERR191790 | ERR191790 | 0.500 | 0.250 | 4.000 | 2.000 | 0.500 | 0.500 | 32.000 | 16.000 | 2.000 | 2.000 |
| USA | 32a04c59-8353-4c01-bc06-599285aaf81f | ERR223612 | ERR223612 | 0.015 | 0.016 | 0.500 | 0.500 | 8.000 | 8.000 | 0.015 | 0.016 | 1.000 | 1.000 |
| USA | 32a37441-2a7e-4578-aba4-35193977fade | ERR191736 | ERR191736 | 0.250 | 0.250 | 2.000 | 2.000 | 0.500 | 0.500 | 16.000 | 16.000 | 2.000 | 2.000 |
| USA | 34ff6402-e9b4-4c00-9b85-e69e8b0a3694 | ERR191804 | ERR191804 | 0.250 | 0.250 | 2.000 | 2.000 | 0.500 | 0.500 | 8.000 | 16.000 | 2.000 | 2.000 |
| USA | 36692fbf-28b8-40e4-b52c-27429221c5d3 | ERR223672 | ERR223672 | 0.015 | 0.031 | 1.000 | 0.500 | 1.000 | 0.500 | 8.000 | 16.000 | 2.000 | 2.000 |
| USA | 37606c91-a29d-4c80-97bd-e72a9b3701cd | ERR223643 | ERR223643 | 0.250 | 0.250 | 2.000 | 2.000 | 0.500 | 0.500 | 32.000 | 16.000 | 2.000 | 2.000 |
| USA | 384be17c-5395-42b4-9875-e52757fab368 | ERR223607 | ERR223607 | 0.250 | 0.250 | 2.000 | 2.000 | 0.125 | 0.500 | 16.000 | 16.000 | 2.000 | 2.000 |
| USA | 390f7f7d-a339-45fd-9e17-b9fa4cf424b6 | ERR191813 | ERR191813 | 0.015 | 0.031 | 2.000 | 0.500 | 1.000 | 0.500 | 0.015 | 0.016 | 4.000 | 2.000 |
| USA | 3ad7e458-c9fd-4a0a-be8e-d77d2e8c49a0 | ERR191785 | ERR191785 | 0.060 | 0.031 | 2.000 | 2.000 | 0.500 | 0.500 | 8.000 | 16.000 | 2.000 | 2.000 |
| USA | 3bc33135-c2a7-4f6d-b1c9-2893ba2927a1 | ERR223687 | ERR223687 | 0.500 | 0.250 | 1.000 | 2.000 | 0.250 | 0.500 | 16.000 | 16.000 | 2.000 | 2.000 |
| USA | 3c4b614b-3765-405a-8030-970c3344903f | ERR191740 | ERR191740 | 0.250 | 0.031 | 2.000 | 1.000 | 0.250 | 0.500 | 0.015 | 0.016 | 0.500 | 1.000 |
| USA | 40c0856b-4330-43c9-8a72-9938b4d43e8a | ERR223656 | ERR223656 | 0.015 | 0.031 | 16.000 | 16.000 | 0.500 | 0.500 | 8.000 | 8.000 | 32.000 | 32.000 |
| USA | 41db39de-754c-46dd-9ab0-283e3aa4b969 | ERR223666 | ERR223666 | 0.015 | 0.031 | 0.250 | 1.000 | 1.000 | 0.500 | 8.000 | 16.000 | 2.000 | 2.000 |
| USA | 4347da75-e2c3-4d21-a4d0-fe737b0fdcbd | ERR191796 | ERR191796 | 0.250 | 0.250 | 4.000 | 2.000 | 0.500 | 0.500 | 16.000 | 16.000 | 2.000 | 2.000 |
| USA | 44bfd709-f2a5-465e-94e9-7dd3587bac75 | ERR191802 | ERR191802 | 0.250 | 0.250 | 1.000 | 1.000 | 0.250 | 0.500 | 0.015 | 0.016 | 0.500 | 1.000 |
| USA | 46140612-6cb5-4dcf-ba55-2f9c114e6e2d | ERR223688 | ERR223688 | 0.015 | 0.016 | 1.000 | 0.500 | 0.500 | 0.500 | 4.000 | 16.000 | 1.000 | 1.000 |
| USA | 46684cc7-f33a-4670-9fb0-9c013c5d9df4 | ERR191824 | ERR191824 | 0.250 | 0.250 | 2.000 | 2.000 | 1.000 | 0.500 | 16.000 | 16.000 | 4.000 | 2.000 |
| USA | 46b007e0-9785-4fa0-be59-7c8a2d9c6aea | ERR223610 | ERR223610 | 0.060 | 0.250 | 4.000 | 2.000 | 2.000 | 2.000 | 16.000 | 16.000 | 4.000 | 2.000 |
| USA | 486694ad-876d-43ae-b9e0-96ae447fefda | ERR223669 | ERR223669 | 0.250 | 0.250 | 4.000 | 2.000 | 0.500 | 0.500 | 16.000 | 16.000 | 4.000 | 2.000 |
| USA | 4b08a0ba-9adc-40b8-a547-40cf7d6b62f1 | ERR191766 | ERR191766 | 0.500 | 0.250 | 2.000 | 2.000 | 0.500 | 0.500 | 32.000 | 16.000 | 4.000 | 2.000 |
| USA | 4f0a2e4e-bc4b-4cc4-bda9-abcd0aa30ec9 | ERR191756 | ERR191756 | 0.250 | 0.250 | 2.000 | 2.000 | 0.500 | 0.500 | 32.000 | 16.000 | 4.000 | 2.000 |
| USA | 5004d0c9-3cdd-4cdf-be8f-04437c7f165c | ERR191763 | ERR191763 | 0.060 | 0.063 | 2.000 | 4.000 | 1.000 | 1.000 | 32.000 | 16.000 | 2.000 | 2.000 |
| USA | 509c7ff7-a86f-4f11-88a9-6d00615f93b5 | ERR191817 | ERR191817 | 0.060 | 0.250 | 0.500 | 2.000 | 0.500 | 0.500 | 0.015 | 16.000 | 0.500 | 2.000 |
| USA | 524a9129-4f0d-436a-a352-31a18d030f02 | ERR223619 | ERR223619 | 0.250 | 0.250 | 2.000 | 2.000 | 0.500 | 0.500 | 32.000 | 16.000 | 4.000 | 2.000 |
| USA | 5345d7a5-2dcd-4e01-960b-d75ec073b3f2 | ERR223630 | ERR223630 | 0.015 | 0.250 | 0.250 | 2.000 | 0.030 | 0.500 | 0.015 | 16.000 | 0.250 | 2.000 |
| USA | 54e7d69d-4e5a-4f68-9193-a671eb056426 | ERR191807 | ERR191807 | 0.015 | 0.031 | 0.250 | 2.000 | 0.060 | 0.500 | 4.000 | 16.000 | 0.250 | 2.000 |
| USA | 5933482c-686d-45b7-8c5a-b9542629c311 | ERR223693 | ERR223693 | 0.250 | 0.250 | 2.000 | 2.000 | 0.250 | 0.500 | 16.000 | 16.000 | 2.000 | 2.000 |
| USA | 5a467b3f-bda6-4642-8883-87c4fe587ba1 | ERR191782 | ERR191782 | 0.250 | 0.250 | 2.000 | 2.000 | 1.000 | 0.500 | 16.000 | 16.000 | 4.000 | 2.000 |
| USA | 5c173e72-fe25-4ee6-88e0-fc364ab5d431 | ERR191789 | ERR191789 | 0.060 | 0.031 | 4.000 | 2.000 | 0.500 | 0.500 | 8.000 | 16.000 | 2.000 | 2.000 |
| USA | 5f059146-36cb-497d-97b8-4c7d6267b1ed | ERR191777 | ERR191777 | 0.060 | 0.250 | 4.000 | 2.000 | 1.000 | 0.500 | 16.000 | 16.000 | 4.000 | 2.000 |
| USA | 5fd5d9af-1c91-498e-b672-5cecdf0198a1 | ERR223698 | ERR223698 | 0.030 | 0.016 | 0.500 | 0.250 | 2.000 | 0.500 | 0.015 | 0.016 | 1.000 | 1.000 |
| USA | 62e61e90-4d0c-4e6f-b755-f8b83e832ec1 | ERR223629 | ERR223629 | 0.250 | 0.250 | 2.000 | 2.000 | 0.500 | 0.500 | 16.000 | 16.000 | 2.000 | 2.000 |
| USA | 63ac60ed-d478-429d-bb44-53258fb2c681 | ERR191731 | ERR191731 | 0.015 | 0.031 | 1.000 | 4.000 | 1.000 | 0.500 | 16.000 | 16.000 | 4.000 | 2.000 |
| USA | 649e84c6-11e8-4496-b83e-c56d0f6b8408 | ERR191735 | ERR191735 | 0.015 | 0.031 | 2.000 | 2.000 | 1.000 | 0.500 | 8.000 | 16.000 | 1.000 | 1.000 |
| USA | 655697eb-90c7-4f6d-a803-200e10fb0065 | ERR191814 | ERR191814 | 0.250 | 0.250 | 2.000 | 2.000 | 0.500 | 0.500 | 32.000 | 16.000 | 2.000 | 2.000 |
| USA | 65d71191-ffed-4ea2-a031-0da44fae304e | ERR223653 | ERR223653 | 0.250 | 0.250 | 2.000 | 2.000 | 1.000 | 0.500 | 16.000 | 16.000 | 4.000 | 2.000 |
| USA | 660eab55-6740-40fe-9ae7-b12abb30ec7e | ERR223674 | ERR223674 | 0.060 | 0.031 | 4.000 | 2.000 | 1.000 | 0.500 | 16.000 | 16.000 | 4.000 | 2.000 |
| USA | 684bf4d3-4122-4334-b359-7f88accc0e59 | ERR223649 | ERR223649 | 0.250 | 0.250 | 4.000 | 2.000 | 0.500 | 0.500 | 16.000 | 16.000 | 2.000 | 2.000 |
| USA | 694b8cbc-b586-4624-ba38-d785baee8e3a | ERR191820 | ERR191820 | 0.250 | 0.250 | 4.000 | 2.000 | 1.000 | 0.500 | 16.000 | 16.000 | 4.000 | 2.000 |
| USA | 6a47bb1a-b9e3-4dbd-9ed9-96ac72aaed7b | ERR223657 | ERR223657 | 0.250 | 0.250 | 2.000 | 2.000 | 1.000 | 0.500 | 16.000 | 16.000 | 2.000 | 2.000 |
| USA | 6ace46f7-20e7-4333-b2e5-12bef28d1a0f | ERR191809 | ERR191809 | 0.015 | 0.031 | 4.000 | 4.000 | 1.000 | 0.500 | 16.000 | 16.000 | 4.000 | 2.000 |
| USA | 6af54540-da1e-4169-b5ad-705fd94a0be6 | ERR223671 | ERR223671 | 0.250 | 0.250 | 2.000 | 2.000 | 1.000 | 0.500 | 16.000 | 16.000 | 4.000 | 2.000 |
| USA | 6c14bdb3-9f61-4730-bb78-0b591c996dbf | ERR191784 | ERR191784 | 0.250 | 0.250 | 2.000 | 2.000 | 1.000 | 0.500 | 16.000 | 16.000 | 2.000 | 2.000 |
| USA | 6d653864-4e95-43f8-95a9-a0067fdfd307 | ERR191749 | ERR191749 | 0.015 | 0.016 | 1.000 | 0.500 | 0.500 | 0.500 | 2.000 | 16.000 | 0.500 | 1.000 |
| USA | 704e6d9c-6e81-4318-9608-a34eb696ddde | ERR191801 | ERR191801 | 0.015 | 0.016 | 0.500 | 1.000 | 0.500 | 0.500 | 0.015 | 0.016 | 0.250 | 0.125 |
| USA | 70dc0e07-a046-4bce-a1f5-d045e6a306ba | ERR191772 | ERR191772 | 0.250 | 0.250 | 4.000 | 2.000 | 1.000 | 0.500 | 16.000 | 16.000 | 2.000 | 2.000 |
| USA | 72ce49ae-fcde-4f91-9444-50676819c775 | ERR223608 | ERR223608 | 0.060 | 0.250 | 4.000 | 2.000 | 2.000 | 4.000 | 16.000 | 16.000 | 2.000 | 2.000 |
| USA | 735d8e6a-dfac-4a39-b1be-8e3f1001e95d | ERR223650 | ERR223650 | 0.060 | 0.016 | 0.500 | 0.500 | 0.500 | 0.500 | 0.015 | 0.016 | 1.000 | 1.000 |
| USA | 74f0c901-6fc0-4f0b-92c4-b9d343255a3d | ERR223660 | ERR223660 | 0.015 | 0.016 | 32.000 | 4.000 | 0.500 | 0.250 | 4.000 | 8.000 | 16.000 | 16.000 |
| USA | 76312a90-56c1-4c57-be93-02035934c2c2 | ERR223686 | ERR223686 | 0.015 | 0.031 | 1.000 | 1.000 | 0.250 | 0.250 | 2.000 | 16.000 | 16.000 | 32.000 |
| USA | 764e80ec-0ddd-469a-91f3-a1d479325b65 | ERR223694 | ERR223694 | 0.030 | 0.016 | 0.250 | 0.500 | 0.250 | 0.250 | 1.000 | 16.000 | 16.000 | 16.000 |
| USA | 793cfca0-2849-43b1-b025-2fbc3fa0f99f | ERR223690 | ERR223690 | 0.015 | 0.016 | 16.000 | 8.000 | 0.030 | 0.500 | 2.000 | 8.000 | 0.250 | 1.000 |
| USA | 7add30ab-1ce3-4b4d-939e-1d60ebdce25b | ERR223683 | ERR223683 | 0.250 | 0.250 | 1.000 | 1.000 | 1.000 | 0.500 | 4.000 | 16.000 | 1.000 | 1.000 |
| USA | 7aef6061-5950-438a-bc5c-ce9f22c035ff | ERR223624 | ERR223624 | 0.030 | 0.016 | 0.250 | 0.250 | 8.000 | 4.000 | 0.015 | 0.016 | 1.000 | 1.000 |
| USA | 7b11b424-b1f9-4444-ab21-2ad000ab7153 | ERR191779 | ERR191779 | 0.015 | 0.016 | 0.250 | 0.500 | 0.030 | 0.500 | 0.015 | 0.016 | 0.250 | 0.125 |
| USA | 7bdd4295-a915-4e69-a110-af85f442684a | ERR223622 | ERR223622 | 0.015 | 0.031 | 0.250 | 2.000 | 0.060 | 0.500 | 4.000 | 16.000 | 0.500 | 2.000 |
| USA | 7c649f1b-7650-4bc2-8942-d837ef78645a | ERR223664 | ERR223664 | 0.030 | 0.031 | 4.000 | 4.000 | 0.250 | 0.500 | 16.000 | 16.000 | 2.000 | 2.000 |
| USA | 7dcf2972-2e21-407a-966a-c05d484f54d3 | ERR191750 | ERR191750 | 0.250 | 0.250 | 4.000 | 2.000 | 1.000 | 0.500 | 32.000 | 16.000 | 2.000 | 2.000 |
| USA | 7ed8c660-cf4d-47f9-80f4-1cd895bf0519 | ERR223638 | ERR223638 | 0.060 | 0.031 | 4.000 | 4.000 | 0.500 | 0.500 | 16.000 | 16.000 | 2.000 | 2.000 |
| USA | 7f1da0d1-76c8-47ec-b4e7-61fea965a578 | ERR191754 | ERR191754 | 0.250 | 0.250 | 2.000 | 2.000 | 0.500 | 0.500 | 16.000 | 16.000 | 2.000 | 2.000 |
| USA | 80de4164-6262-498b-a17e-f251312253d3 | ERR223616 | ERR223616 | 0.030 | 0.016 | 0.250 | 0.250 | 16.000 | 8.000 | 0.015 | 0.016 | 1.000 | 1.000 |
| USA | 837533b7-3594-4628-a4ee-b117f708cbb5 | ERR223623 | ERR223623 | 0.250 | 0.250 | 1.000 | 2.000 | 0.250 | 0.500 | 16.000 | 16.000 | 2.000 | 2.000 |
| USA | 84e01fbd-5329-4bad-b7a6-729fb3d805d0 | ERR191816 | ERR191816 | 0.250 | 0.250 | 2.000 | 2.000 | 0.500 | 0.500 | 32.000 | 16.000 | 4.000 | 2.000 |
| USA | 888eec8d-c094-465c-8e93-a4bc79588420 | ERR223696 | ERR223696 | 0.030 | 0.031 | 4.000 | 2.000 | 1.000 | 0.500 | 16.000 | 16.000 | 4.000 | 2.000 |
| USA | 8a61bd72-e265-4589-8012-50463e2cfc3a | ERR223635 | ERR223635 | 0.250 | 0.250 | 2.000 | 2.000 | 0.500 | 0.500 | 32.000 | 16.000 | 4.000 | 2.000 |
| USA | 8ad7553d-aa18-4a39-b4bb-b06ab3177420 | ERR223697 | ERR223697 | 0.250 | 0.250 | 1.000 | 1.000 | 0.500 | 0.500 | 0.015 | 0.016 | 1.000 | 1.000 |
| USA | 8e54afff-b4f9-4ff7-91be-3b447161809c | ERR191815 | ERR191815 | 0.030 | 0.031 | 2.000 | 2.000 | 1.000 | 0.500 | 4.000 | 16.000 | 1.000 | 1.000 |
| USA | 8e9eb1de-c7e7-4fe6-b542-c072ad978c7c | ERR223663 | ERR223663 | 0.250 | 0.250 | 8.000 | 2.000 | 1.000 | 0.500 | 16.000 | 16.000 | 4.000 | 2.000 |
| USA | 8eb3d960-10db-4a60-9454-0c1e19574e02 | ERR223633 | ERR223633 | 0.250 | 0.250 | 1.000 | 1.000 | 0.250 | 0.500 | 8.000 | 16.000 | 0.500 | 1.000 |
| USA | 8ebbe201-221a-4327-a180-76cfc4e52310 | ERR223621 | ERR223621 | 0.500 | 0.250 | 4.000 | 2.000 | 0.500 | 0.500 | 16.000 | 16.000 | 4.000 | 2.000 |
| USA | 8f842c3c-d0a8-4705-89d4-91c26746f5b0 | ERR223644 | ERR223644 | 0.015 | 0.016 | 0.250 | 0.500 | 16.000 | 16.000 | 0.015 | 0.016 | 1.000 | 1.000 |
| USA | 9274c2ce-8f60-4c8e-8f70-bdc4b2f2dfac | ERR191808 | ERR191808 | 0.250 | 0.250 | 2.000 | 2.000 | 0.500 | 0.500 | 16.000 | 16.000 | 1.000 | 2.000 |
| USA | 929eb8d2-ca00-4049-a886-8217f6108ea3 | ERR191760 | ERR191760 | 0.500 | 0.250 | 2.000 | 2.000 | 0.500 | 0.500 | 32.000 | 16.000 | 4.000 | 2.000 |
| USA | 95f06af8-0353-49d1-8ead-fabcaba7ddaf | ERR223615 | ERR223615 | 0.250 | 0.250 | 4.000 | 2.000 | 0.500 | 0.500 | 32.000 | 16.000 | 4.000 | 2.000 |
| USA | 96c3f735-df10-437d-ab0a-ccdb094d2f6c | ERR191780 | ERR191780 | 0.250 | 0.250 | 2.000 | 2.000 | 0.500 | 0.500 | 16.000 | 16.000 | 2.000 | 2.000 |
| USA | 96d58bb6-9013-461f-ba6f-0079fb9af208 | ERR223689 | ERR223689 | 0.250 | 0.250 | 1.000 | 2.000 | 0.250 | 0.500 | 16.000 | 16.000 | 2.000 | 2.000 |
| USA | 98cd4610-4b93-4ed7-b86a-4fbb64f31f42 | ERR191821 | ERR191821 | 0.060 | 0.016 | 1.000 | 0.500 | 0.250 | 0.500 | 0.015 | 0.016 | 1.000 | 1.000 |
| USA | 990c7d52-2161-4017-a5aa-09c2c46baa2d | ERR191771 | ERR191771 | 0.060 | 0.031 | 4.000 | 4.000 | 1.000 | 0.500 | 8.000 | 16.000 | 4.000 | 2.000 |
| USA | 9a308e71-2751-47a7-8cc6-41838607692b | ERR223639 | ERR223639 | 0.250 | 0.250 | 2.000 | 2.000 | 0.500 | 0.500 | 32.000 | 16.000 | 2.000 | 2.000 |
| USA | 9b7c87c8-d111-4503-b836-8bbdef2c9039 | ERR191759 | ERR191759 | 0.015 | 0.031 | 0.500 | 0.500 | 1.000 | 0.500 | 8.000 | 16.000 | 2.000 | 2.000 |
| USA | 9c5bdb4a-14e6-4ace-9003-edfd388fe45c | ERR191746 | ERR191746 | 0.250 | 0.250 | 4.000 | 2.000 | 1.000 | 0.500 | 32.000 | 16.000 | 4.000 | 2.000 |
| USA | 9d8df89d-553c-486a-adc5-6b4ee41b7454 | ERR191822 | ERR191822 | 0.250 | 0.250 | 4.000 | 2.000 | 1.000 | 0.500 | 16.000 | 16.000 | 4.000 | 2.000 |
| USA | 9df78dc0-69aa-4bca-b964-59b6ad29abe6 | ERR223613 | ERR223613 | 0.250 | 0.250 | 4.000 | 2.000 | 0.500 | 0.500 | 16.000 | 16.000 | 4.000 | 2.000 |
| USA | 9eeafdb6-5018-454a-991d-087f82ed4b38 | ERR191741 | ERR191741 | 0.060 | 0.125 | 1.000 | 1.000 | 0.250 | 0.500 | 0.015 | 0.016 | 0.500 | 1.000 |
| USA | 9f0a3533-9d9b-4bb3-aed5-80be81da1548 | ERR223631 | ERR223631 | 0.250 | 0.250 | 4.000 | 2.000 | 0.500 | 0.500 | 16.000 | 16.000 | 2.000 | 2.000 |
| USA | 9f266662-39bd-458c-8edd-baa2a51f7ed3 | ERR191787 | ERR191787 | 0.030 | 0.031 | 2.000 | 4.000 | 0.500 | 0.500 | 16.000 | 16.000 | 2.000 | 2.000 |
| USA | a0a28e6b-31d9-42da-9e83-f296f2f4b180 | ERR223604 | ERR223604 | 0.030 | 0.016 | 0.250 | 0.250 | 16.000 | 8.000 | 0.015 | 0.016 | 1.000 | 1.000 |
| USA | a3ba58ff-df55-4e6e-a37b-498bc2ad87c1 | ERR223682 | ERR223682 | 0.015 | 0.016 | 0.250 | 0.250 | 16.000 | 8.000 | 0.015 | 0.016 | 1.000 | 1.000 |
| USA | a3e6e376-9b0e-456d-ad92-db1f781b133a | ERR223647 | ERR223647 | 0.250 | 0.250 | 4.000 | 2.000 | 0.500 | 0.500 | 16.000 | 16.000 | 4.000 | 2.000 |
| USA | a57bc4b9-5082-4f72-a3f0-48b2f496c0ad | ERR191825 | ERR191825 | 0.030 | 0.016 | 0.500 | 0.250 | 16.000 | 8.000 | 0.015 | 0.016 | 1.000 | 1.000 |
| USA | a62ee71e-1f36-4210-873c-ecee55faf213 | ERR191755 | ERR191755 | 0.060 | 0.031 | 8.000 | 2.000 | 0.500 | 0.500 | 16.000 | 16.000 | 4.000 | 2.000 |
| USA | a7c24385-76f3-48c3-8daa-8805ec3e4cac | ERR191758 | ERR191758 | 0.250 | 0.250 | 2.000 | 2.000 | 0.500 | 0.500 | 32.000 | 16.000 | 4.000 | 2.000 |
| USA | a82e091f-336a-4d8f-b80c-7e17aa35e74e | ERR191818 | ERR191818 | 0.250 | 0.250 | 2.000 | 2.000 | 1.000 | 0.500 | 32.000 | 16.000 | 16.000 | 2.000 |
| USA | a89fa9d7-e9a6-408c-b7a7-236c51aefae9 | ERR223634 | ERR223634 | 0.030 | 0.016 | 0.250 | 0.500 | 8.000 | 16.000 | 0.015 | 0.016 | 0.500 | 1.000 |
| USA | a9f08bec-7bac-471a-b1bf-89f91418c70c | ERR223640 | ERR223640 | 0.030 | 0.031 | 4.000 | 2.000 | 0.500 | 0.500 | 16.000 | 16.000 | 2.000 | 2.000 |
| USA | aa1bb7c1-73d0-406d-8ffc-8ff3c89dd2ac | ERR191783 | ERR191783 | 0.060 | 0.031 | 2.000 | 4.000 | 1.000 | 0.500 | 16.000 | 16.000 | 4.000 | 2.000 |
| USA | aa419780-2753-466c-9986-06089b638ff0 | ERR223632 | ERR223632 | 0.060 | 0.250 | 0.500 | 0.500 | 0.250 | 0.500 | 0.015 | 0.016 | 0.500 | 1.000 |
| USA | aebb8b9c-26e6-442e-84f9-c5faf95f952b | ERR223646 | ERR223646 | 0.060 | 0.031 | 4.000 | 2.000 | 1.000 | 0.500 | 32.000 | 16.000 | 4.000 | 2.000 |
| USA | b082f61a-3583-4a4d-a7a8-b8094901e48a | ERR223603 | ERR223603 | 0.500 | 0.250 | 2.000 | 2.000 | 0.500 | 0.500 | 16.000 | 16.000 | 4.000 | 2.000 |
| USA | b0cb1b76-73f5-4c64-8909-a7c910a5d7df | ERR191734 | ERR191734 | 0.500 | 0.250 | 4.000 | 2.000 | 1.000 | 0.500 | 16.000 | 16.000 | 4.000 | 2.000 |
| USA | b120fb73-80ce-4aae-96eb-ee8307a2a43d | ERR223648 | ERR223648 | 0.015 | 0.031 | 0.500 | 1.000 | 2.000 | 8.000 | 0.015 | 0.016 | 1.000 | 2.000 |
| USA | b132fd1e-8144-41c0-9e01-4f97b4cd5816 | ERR191769 | ERR191769 | 0.030 | 0.063 | 16.000 | 16.000 | 0.125 | 0.500 | 16.000 | 16.000 | 16.000 | 32.000 |
| USA | b2552657-c176-4777-9f3f-1aaf18dc5cc9 | ERR223637 | ERR223637 | 0.250 | 0.250 | 2.000 | 2.000 | 0.500 | 0.500 | 32.000 | 16.000 | 4.000 | 2.000 |
| USA | b2f2a7b3-a3d2-48ea-b185-cae4d6a6c687 | ERR191788 | ERR191788 | 0.250 | 0.250 | 4.000 | 2.000 | 1.000 | 0.500 | 16.000 | 16.000 | 4.000 | 2.000 |
| USA | b619abf6-ef29-4447-9061-47e85a67bbc1 | ERR191806 | ERR191806 | 0.250 | 0.250 | 4.000 | 2.000 | 1.000 | 0.500 | 16.000 | 16.000 | 2.000 | 2.000 |
| USA | b661fca0-a6c4-4636-bf3c-8984e738d94e | ERR223625 | ERR223625 | 0.250 | 0.250 | 1.000 | 2.000 | 0.250 | 0.500 | 16.000 | 16.000 | 2.000 | 2.000 |
| USA | b714fba2-6799-4fb4-b490-b3074dd5d7af | ERR223652 | ERR223652 | 0.030 | 0.016 | 4.000 | 4.000 | 0.250 | 0.250 | 1.000 | 16.000 | 16.000 | 16.000 |
| USA | b8689c28-4173-4659-a6df-5b177a0d5d9c | ERR223658 | ERR223658 | 0.060 | 0.031 | 2.000 | 2.000 | 0.500 | 0.500 | 16.000 | 16.000 | 2.000 | 2.000 |
| USA | b88fd52e-541d-4061-8631-865facf8ebcf | ERR223606 | ERR223606 | 0.060 | 0.125 | 2.000 | 1.000 | 0.250 | 0.500 | 0.015 | 0.016 | 1.000 | 1.000 |
| USA | b8fd78f7-2ad9-4691-88d8-2ebe9e491320 | ERR191752 | ERR191752 | 0.250 | 0.250 | 2.000 | 2.000 | 0.500 | 0.500 | 16.000 | 16.000 | 2.000 | 2.000 |
| USA | b9afcf04-f42f-4060-a2d4-414dabdaa3ea | ERR191795 | ERR191795 | 0.015 | 0.031 | 1.000 | 0.500 | 1.000 | 0.500 | 8.000 | 16.000 | 4.000 | 2.000 |
| USA | b9dec2af-198c-475f-8902-775d77d71bba | ERR191810 | ERR191810 | 0.250 | 0.250 | 2.000 | 2.000 | 0.125 | 0.500 | 16.000 | 16.000 | 1.000 | 2.000 |
| USA | bd9adafa-40ae-4b9c-919c-7a806e8e1bfb | ERR223684 | ERR223684 | 0.030 | 0.016 | 1.000 | 0.500 | 1.000 | 0.500 | 16.000 | 16.000 | 2.000 | 2.000 |
| USA | bdba6377-db79-4a49-b511-185b0c4a8f6a | ERR223662 | ERR223662 | 0.030 | 0.016 | 0.250 | 0.250 | 16.000 | 8.000 | 0.015 | 0.016 | 1.000 | 1.000 |
| USA | bdd26924-1712-407f-aba3-9769efc2aac6 | ERR191765 | ERR191765 | 0.030 | 0.031 | 2.000 | 2.000 | 0.500 | 0.500 | 16.000 | 16.000 | 1.000 | 2.000 |
| USA | bf61c3c1-22ff-4765-8009-bc3ac5bac5a7 | ERR191738 | ERR191738 | 0.250 | 0.031 | 1.000 | 0.500 | 0.250 | 0.500 | 0.015 | 0.016 | 2.000 | 1.000 |
| USA | bfe94fe7-a792-4abb-b5fc-e923ef2b7601 | ERR223675 | ERR223675 | 0.250 | 0.250 | 1.000 | 1.000 | 0.500 | 0.500 | 0.015 | 0.016 | 1.000 | 1.000 |
| USA | c214fc05-75a0-4793-aefe-1ab4e1b6b70b | ERR223685 | ERR223685 | 0.500 | 0.250 | 2.000 | 2.000 | 0.250 | 0.500 | 16.000 | 16.000 | 2.000 | 2.000 |
| USA | c229701c-4b87-4ce9-9a97-c75f6e50b9f6 | ERR223642 | ERR223642 | 0.030 | 0.016 | 0.250 | 0.500 | 8.000 | 16.000 | 0.015 | 0.016 | 1.000 | 1.000 |
| USA | c5254dad-944f-4821-a2c6-4a481d929fd3 | ERR191798 | ERR191798 | 0.250 | 0.250 | 4.000 | 2.000 | 1.000 | 0.500 | 16.000 | 16.000 | 4.000 | 2.000 |
| USA | c6e390af-3014-4b37-aa56-d5c70b669542 | ERR223676 | ERR223676 | 0.060 | 0.031 | 4.000 | 2.000 | 0.500 | 0.500 | 16.000 | 16.000 | 4.000 | 2.000 |
| USA | c7d58e83-eaea-489b-8e42-0ee194ef7278 | ERR191733 | ERR191733 | 0.015 | 0.031 | 2.000 | 4.000 | 1.000 | 0.500 | 16.000 | 16.000 | 4.000 | 2.000 |
| USA | cabae1e5-2bed-4d4b-9f47-9694b78fb055 | ERR191778 | ERR191778 | 0.250 | 0.250 | 2.000 | 2.000 | 1.000 | 0.500 | 16.000 | 16.000 | 4.000 | 2.000 |
| USA | cb8dacd6-c73f-4fc2-bb06-3080f44e30c4 | ERR191773 | ERR191773 | 0.060 | 0.031 | 4.000 | 2.000 | 0.500 | 0.500 | 16.000 | 16.000 | 4.000 | 2.000 |
| USA | cbc23230-7089-48e5-ae85-8201c514b415 | ERR191781 | ERR191781 | 0.030 | 0.031 | 2.000 | 4.000 | 0.500 | 0.500 | 8.000 | 16.000 | 2.000 | 2.000 |
| USA | cd8c922d-82da-4538-ae7b-56b42014c6da | ERR223659 | ERR223659 | 0.250 | 0.250 | 0.500 | 1.000 | 0.500 | 0.500 | 0.015 | 0.016 | 0.500 | 1.000 |
| USA | cdeae2c2-77b1-4fe3-b3ab-812e9ac7619a | ERR223668 | ERR223668 | 0.015 | 0.016 | 1.000 | 2.000 | 0.500 | 0.250 | 0.015 | 0.016 | 0.500 | 1.000 |
| USA | d6c9ddaa-d7c3-4f7d-ac57-f72342d74810 | ERR191739 | ERR191739 | 0.015 | 0.016 | 0.250 | 0.500 | 0.500 | 0.500 | 0.015 | 0.016 | 0.500 | 1.000 |
| USA | d73463fc-7b08-4cc5-8a7b-cd0910c5f621 | ERR191799 | ERR191799 | 0.030 | 0.031 | 1.000 | 2.000 | 0.500 | 0.500 | 4.000 | 16.000 | 1.000 | 1.000 |
| USA | d8504604-41ee-4252-b705-5c665911713b | ERR223641 | ERR223641 | 0.500 | 0.250 | 2.000 | 2.000 | 0.500 | 0.500 | 16.000 | 16.000 | 4.000 | 2.000 |
| USA | d92b7063-1490-4f29-b447-a8011b6f256f | ERR191805 | ERR191805 | 0.015 | 0.031 | 1.000 | 0.500 | 0.250 | 0.500 | 8.000 | 16.000 | 2.000 | 2.000 |
| USA | dbabeaa4-2a6d-4377-b57a-48148468de65 | ERR191737 | ERR191737 | 0.015 | 0.031 | 0.500 | 0.500 | 0.250 | 0.500 | 1.000 | 16.000 | 16.000 | 16.000 |
| USA | dc541e91-1fad-4711-b289-1cff003b9d4b | ERR191823 | ERR191823 | 0.030 | 0.031 | 2.000 | 2.000 | 1.000 | 0.500 | 8.000 | 16.000 | 2.000 | 1.000 |
| USA | dc7fc48c-740a-4be0-ac49-40c12d0ddb75 | ERR191748 | ERR191748 | 0.250 | 0.250 | 4.000 | 2.000 | 1.000 | 0.500 | 32.000 | 16.000 | 4.000 | 2.000 |
| USA | de45977a-e918-4358-9350-90fcfcd104fa | ERR223636 | ERR223636 | 0.030 | 0.031 | 2.000 | 2.000 | 0.500 | 0.500 | 16.000 | 16.000 | 2.000 | 2.000 |
| USA | e0d41e41-70e1-4070-b888-9059992a7d81 | ERR223670 | ERR223670 | 0.030 | 0.031 | 4.000 | 2.000 | 0.250 | 0.500 | 16.000 | 16.000 | 2.000 | 2.000 |
| USA | e228f26c-d9e6-4a44-b70e-4828c8c87583 | ERR191757 | ERR191757 | 0.015 | 0.031 | 0.500 | 0.500 | 1.000 | 0.500 | 8.000 | 16.000 | 2.000 | 2.000 |
| USA | e4501091-330f-4954-8866-da41f2c1376d | ERR191762 | ERR191762 | 0.250 | 0.250 | 4.000 | 2.000 | 0.250 | 0.500 | 16.000 | 16.000 | 4.000 | 2.000 |
| USA | e4d9e29b-a653-47d2-9908-02c168c0508a | ERR223645 | ERR223645 | 0.250 | 0.250 | 4.000 | 2.000 | 1.000 | 0.500 | 32.000 | 16.000 | 4.000 | 2.000 |
| USA | e59c0171-8dbd-4a98-baf8-b2103a9dd6f1 | ERR223677 | ERR223677 | 0.250 | 0.250 | 1.000 | 1.000 | 0.500 | 0.500 | 0.015 | 0.016 | 1.000 | 1.000 |
| USA | e64f6d26-20ea-4205-9e1d-a25c41f18279 | ERR223627 | ERR223627 | 0.500 | 0.250 | 2.000 | 2.000 | 0.500 | 0.500 | 16.000 | 16.000 | 2.000 | 2.000 |
| USA | e7a6678b-9383-4f5d-90ef-7884650e511b | ERR223626 | ERR223626 | 0.015 | 0.016 | 0.250 | 0.250 | 8.000 | 8.000 | 0.015 | 0.016 | 0.500 | 1.000 |
| USA | e9e58924-0e46-475d-b11b-f9813ae2abcc | ERR223655 | ERR223655 | 0.250 | 0.250 | 1.000 | 1.000 | 0.500 | 0.500 | 0.015 | 0.016 | 1.000 | 1.000 |
| USA | eaf98456-81d6-4273-b487-a4c7cefbbb10 | ERR223605 | ERR223605 | 0.500 | 0.250 | 4.000 | 2.000 | 1.000 | 0.500 | 16.000 | 16.000 | 4.000 | 2.000 |
| USA | ebb7b80d-6f6d-47a7-b33f-4c1ab1a49a19 | ERR191786 | ERR191786 | 0.250 | 0.250 | 2.000 | 2.000 | 0.500 | 0.500 | 16.000 | 16.000 | 2.000 | 2.000 |
| USA | ef5a7c8c-d903-4ec1-b470-2f2532190b61 | ERR223673 | ERR223673 | 0.250 | 0.250 | 1.000 | 1.000 | 0.500 | 0.500 | 0.015 | 0.016 | 1.000 | 1.000 |
| USA | f1146edb-57b4-4612-b875-a2a9a7c6536c | ERR223695 | ERR223695 | 0.250 | 0.250 | 4.000 | 2.000 | 1.000 | 0.500 | 16.000 | 16.000 | 2.000 | 2.000 |
| USA | f51b222d-5d96-44a4-ac14-5682332629ae | ERR223679 | ERR223679 | 0.250 | 0.250 | 1.000 | 1.000 | 1.000 | 0.500 | 0.015 | 0.016 | 1.000 | 1.000 |
| USA | f5d59c5e-a390-433d-81f9-9be6038e5f98 | ERR223661 | ERR223661 | 0.250 | 0.250 | 4.000 | 2.000 | 1.000 | 0.500 | 16.000 | 16.000 | 4.000 | 2.000 |
| USA | f6184481-5728-4d17-a616-5a23a6278f0b | ERR191791 | ERR191791 | 0.015 | 0.016 | 0.500 | 0.250 | 0.250 | 0.500 | 4.000 | 16.000 | 1.000 | 1.000 |
| USA | f72c53a9-25b7-4c9f-b926-ce66efefbc9f | ERR191770 | ERR191770 | 0.250 | 0.250 | 4.000 | 2.000 | 1.000 | 0.500 | 16.000 | 16.000 | 4.000 | 2.000 |
| USA | f7861967-ca37-4d13-ad34-7630b4bd4bb7 | ERR191794 | ERR191794 | 0.250 | 0.250 | 2.000 | 2.000 | 0.500 | 0.500 | 16.000 | 16.000 | 4.000 | 2.000 |
| USA | f7b03a20-da4a-4f5c-93df-4880e0178619 | ERR223628 | ERR223628 | 0.060 | 0.031 | 2.000 | 2.000 | 0.250 | 0.500 | 16.000 | 16.000 | 2.000 | 2.000 |
| USA | f9e706ad-4157-43d4-8e5d-f187676997f1 | ERR223614 | ERR223614 | 0.030 | 0.031 | 2.000 | 2.000 | 0.250 | 0.500 | 16.000 | 16.000 | 4.000 | 2.000 |
| USA | fb4e3606-6061-40fe-a14e-4c593d9dcd25 | ERR191803 | ERR191803 | 0.015 | 0.016 | 0.500 | 0.500 | 0.250 | 0.500 | 0.015 | 0.016 | 0.500 | 1.000 |
| USA | fe88e0f4-a2aa-4465-a138-70f17a609e58 | ERR223678 | ERR223678 | 0.015 | 0.031 | 1.000 | 4.000 | 2.000 | 0.500 | 0.015 | 0.016 | 1.000 | 1.000 |

**Table S2. Study samples**.
